# Selective intraoperative cholangiography should be considered over routine intraoperative cholangiography during cholecystectomy: a systematic review and meta-analysis

**DOI:** 10.1007/s00464-022-09267-x

**Published:** 2022-07-07

**Authors:** Norbert Kovács, Dávid Németh, Mária Földi, Bernadette Nagy, Stefania Bunduc, Péter Hegyi, Judit Bajor, Katalin Eszter Müller, Áron Vincze, Bálint Erőss, Szabolcs Ábrahám

**Affiliations:** 1 Doctoral School of Clinical Medicine, University of Szeged, Szeged, Hungary; 2 Institute for Translational Medicine, University of Pécs Medical School, Pécs, Hungary; 3 Institute of Bioanalysis, University of Pécs Medical School, Pécs, Hungary; 4 Institute for Translational Medicine, Szentágothai Research Centre, University of Pécs Medical School, Pécs, Hungary; 5 Gastroenterology, Hepatology and Liver Transplant Department, Fundeni Clinical Institute, Bucharest, Romania; 6 Doctoral School, Carol Davila University of Medicine and Pharmacy, Bucharest, Romania; 7 Centre for Translational Medicine, Semmelweis University, Budapest, Hungary; 8 Division of Pancreatic Diseases, Heart and Vascular Center, Semmelweis University, Budapest, Hungary; 9 Division of Gastroenterology, First Department of Medicine, University of Pécs Medical School, Pécs, Hungary; 10 Heim Pál National Institute of Pediatrics, Budapest, Hungary; 11 Department of Surgery, Szent-Györgyi Albert Medical and Pharmaceutical Centre, University of Szeged, Semmelweis u. 8, 6720 Szeged, Hungary

**Keywords:** Cholecystectomy, Laparoscopic cholecystectomy, Cholangiography, IOC, Bile duct injury, BDI

## Abstract

**Background:**

Decades of debate surround the use of intraoperative cholangiography (IOC) during cholecystectomy. To the present day, the role of IOC is controversial as regards decreasing the rate of bile duct injury (BDI). We aimed to review and analyse the available literature on the benefits of IOC during cholecystectomy.

**Methods:**

A systematic literature search was performed until 19 October 2020 in five databases using the following search keys: cholangiogra* and cholecystectomy. The primary outcomes were BDI and retained stone rate. To investigate the differences between the groups (routine IOC vs selective IOC and IOC vs no IOC), we calculated weighted mean differences (WMD) for continuous outcomes and relative risks (RR) for dichotomous outcomes, with 95% confidence intervals (CI).

**Results:**

Of the 19,863 articles, 38 were selected and 32 were included in the quantitative synthesis. Routine IOC showed no superiority compared to selective IOC in decreasing BDI (RR = 0.91, 95% CI 0.66; 1.24). Comparing IOC and no IOC, no statistically significant differences were found in the case of BDI, retained stone rate, readmission rate, and length of hospital stay. We found an increased risk of conversion rate to open surgery in the no IOC group (RR = 0.64, CI 0.51; 0.78). The operation time was significantly longer in the IOC group compared to the no IOC group (WMD = 11.25 min, 95% CI 6.57; 15.93).

**Conclusion:**

Our findings suggest that IOC may not be indicated in every case, however, the evidence is very uncertain. Further good quality research is required to address this question.

**Supplementary Information:**

The online version contains supplementary material available at 10.1007/s00464-022-09267-x.

Cholecystectomy is one of the most frequently performed surgical interventions. With the advent of laparoscopy, laparoscopic cholecystectomy (LC) became the “gold standard” for the treatment of cholecystolithiasis. It has undeniable advantages over open cholecystectomy: reduced postoperative morbidity, mortality and length of hospital stay, and lower rate of pneumonia and wound infection [1]. Like any surgical intervention, LC carries a risk of complications. Major complications include bile duct injury (BDI), bowel perforation, and vascular injury. Although rare, with an incidence of 0.3% to 0.5%, BDI is a very serious complication of LC [2, 3]. BDI is associated with higher postoperative mortality, morbidity, and decreased quality of life [4]. Several guidelines, meta-analyses, and systematic reviews have been published aiming to provide recommendations for prevention of BDI [4–13]. One of the most investigated methods is intraoperative cholangiography (IOC), but various alternatives (e.g. critical view of safety, laparoscopic ultrasound, and fluorescent cholangiography) have been examined. However, the quality of evidence is low in most of the cases. Most of the literature agrees that IOC has its place in daily practice. Still, due to the low level of evidence and several contradictory articles [14–18], experts have reached no solid consensus.

Advocates of IOC argue that its use during cholecystectomy may reduce the risk of BDI by delineating unclear or aberrant biliary anatomy, aid in intraoperative BDI detection [14, 19], and facilitate perioperative bile duct stone detection, thus decreasing the rate of readmission for retained common bile duct (CBD) stones [13]. Some studies have found that intraoperative detection and treatment of BDI have a beneficial effect on morbidity and mortality [10, 17, 20]. Advocates of IOC therefore suggest a routine combination of LC and IOC. Opponents of IOC argue that when performed routinely, it increases the intraoperative detection of previously asymptomatic bile duct stones. This may result in unnecessary management of CBD stones since only a small percentage of preoperatively asymptomatic and intraoperatively missed bile duct stones became symptomatic after surgery [21]. Further, IOC is a time-consuming procedure, and both staff and patients are exposed to radiation [14]. Hence opponents suggest the omission of IOC.

Some authors support the idea of selective IOC arguing that most CBD stones can be detected preoperatively, and the incidence of BDI is low. [22, 23] IOC may therefore not be necessary routinely, except when CBD stones are suspected or in patients at high risk of BDI.

This systematic review and meta-analysis aims to revise the available literature, thereby providing a comprehensive summary of the topic and giving a better understanding of the necessity of IOC. We identified relevant publications to examine the benefits of routine use, selective use, and omission of IOC during cholecystectomy, and to compare these strategies, especially in terms of BDI and prevention of CBD stone-related complications.

## Materials and methods

We report our systematic review and meta-analysis in line with the Preferred Reporting Items for Systematic Reviews and Meta‐Analyses (PRISMA) Statement [24]. Our systematic review and meta-analysis protocol was previously submitted to PROSPERO under the registration number CRD42021240405. Besides analyses declared in the protocol, we performed a subgroup analysis, including only randomized control trials (RCT) and prospective studies investigating bile duct injury (BDI).

### Search strategy

A systematic literature search was conducted up until 19 October 2020 in Embase, MEDLINE (via PubMed), the Cochrane Central Register of Controlled Trials (CENTRAL), Scopus, and Web of Science using the following search keys: cholangiogra* and cholecystectomy. We searched in all fields/all texts in every database, except in Scopus where the “Article title, Abstract, Keywords” fields were used. We did not apply any filters (e.g. language).

### Selection strategy and eligibility criteria

Software and manual duplicate removal were performed using Endnote X9 (Clarivate Analytics, Philadelphia, PA, USA). The selection process was carried out by two independent authors (BN and NK). The selection process was conducted in stages of selection by title, abstract, and full text. After each step of the selection process, Cohen’s kappa coefficient was calculated to determine the agreement between the two researchers. We removed all unrelated titles, abstracts, and full texts. No report was excluded based on the follow-up period; we only used studies where the follow-up periods were equal or similar for the quantitative synthesis. Grey literature was excluded from our review. Disagreements were resolved by consensus.

The PICO framework was used to define the eligibility criteria. We included articles where the population (P) consisted of laparoscopic cholecystectomy or a mixed population of open and laparoscopic cholecystectomies. Three intervention (I)/comparison (C) groups were formed based on the available literature: IOC vs no IOC, routine IOC vs selective IOC, and selective IOC vs no IOC. All the articles were selected and analysed in which IOC vs no IOC strategy was compared. In the routine IOC group, all the patients received a cholangiography during the cholecystectomy. We considered selective IOC if patients were selected for IOC based on prespecified criteria (clinical, laboratory, or imaging findings). As regards study type, only RCTs and observational studies were considered eligible.

### Outcomes

These groups were examined according to primary outcomes (rate of perioperative bile duct injury and retained stone rate) and secondary outcomes (readmission rate, rate of conversion from laparoscopic procedure to open surgery, the success rate of IOC, operation time (minutes), and length of hospital stay (days)).

BDI was defined as “any tissue damage to the biliary system as a result of surgery”. Retained stones were defined as missed bile duct stones during cholecystectomy that were discovered postoperatively.

### Subgroup analysis

We performed the following subgroup analyses [1]: studies comprising laparoscopic cholecystectomy cases exclusively [2], prospective studies reporting on BDI, and [3] studies involving major bile duct injury (MBDI). We defined MBDI as an injury of the CBD, common hepatic duct, left or right main hepatic duct, or BDI requiring surgical repair.

### Data extraction

The data extraction was performed by two independent authors (BN and NK). Disagreements were resolved by consensus. We used a standardized data collection sheet to collect all the necessary data: first author, publication year, study design, Digital Object Identifier (DOI), type of surgical intervention, nature of comparison (IOC vs no IOC, routine IOC vs selective IOC, and selective IOC vs no IOC), the definition of selective IOC, age and gender distribution in each group, number of patients in each comparison group, and number of events in each group in terms of primary and secondary outcomes.

### Publication bias and risk of bias assessment

A funnel plot and Egger’s test were used to assess the presence of publication bias, where the number of articles allowed it. A funnel plot was created when at least six studies were pooled. We used Egger’s test when at least ten were pooled.

The risk of bias assessment was performed independently by two authors using the ROBINS-I (Risk of Bias in Non-randomized Studies—of Interventions) [25] tool for non-randomized studies and the RoB 2 tool for randomized controlled trials recommended by the Cochrane collaboration [26]. Disagreements were resolved by consensus.

### Statistical analysis

We used the methods recommended by the Cochrane Collaboration working group for data synthesis [27]. A meta-analysis was performed; the calculated effect sizes were visualized on forest plots.

We calculated weighted mean differences (WMD) for continuous outcomes and relative risks (RR) for dichotomous outcomes, both with 95% confidence intervals (CI), to investigate the differences between the three groups (IOC vs no IOC, routine IOC vs selective IOC, and selective IOC vs no IOC).

Heterogeneity was tested both by performing Cochran’s Q test and calculating Higgins’ I^2^ indicator. The Q statistics were calculated as the squared deviations from the pooled effect of the weighted sum of individual study effects, with the weights being used as part of the pooling method; p-values were obtained by comparing the test statistics with a chi-square with k-1 degrees of freedom (where k was the number of studies). A *p*-value of less than 0.10 was considered suggestive of significant heterogeneity. The *I*^2^ index corresponds to the percentage of the total variability across studies that is due to heterogeneity. Based on Cochrane’s handbook, a rough classification of its value is the following: not important (0–40%), moderate (30–60%), substantial (50–90%), and considerable (75–100%) [28]. All the statistical analyses were performed using StataIC (version 16).

### Certainty of evidence

Certainty of evidence was evaluated according to the Grades of Recommendation, Assessment, Development, and Evaluation (GRADE) workgroup recommendations [29]. The outcome assessment was performed for each endpoint by two independent authors (BN and NK), with every disagreement resolved by consensus.

We developed several GRADE evidence profile tables using the GRADEpro GDT software [30] for each comparison group (routine vs selective IOC and IOC vs no IOC) separately. The first table comprises articles that involved a mixed population of open and laparoscopic cholecystectomies. The second table is a subgroup containing only the articles that investigated LC.

## Results

### Results of search and selection

**Fig. 1:**
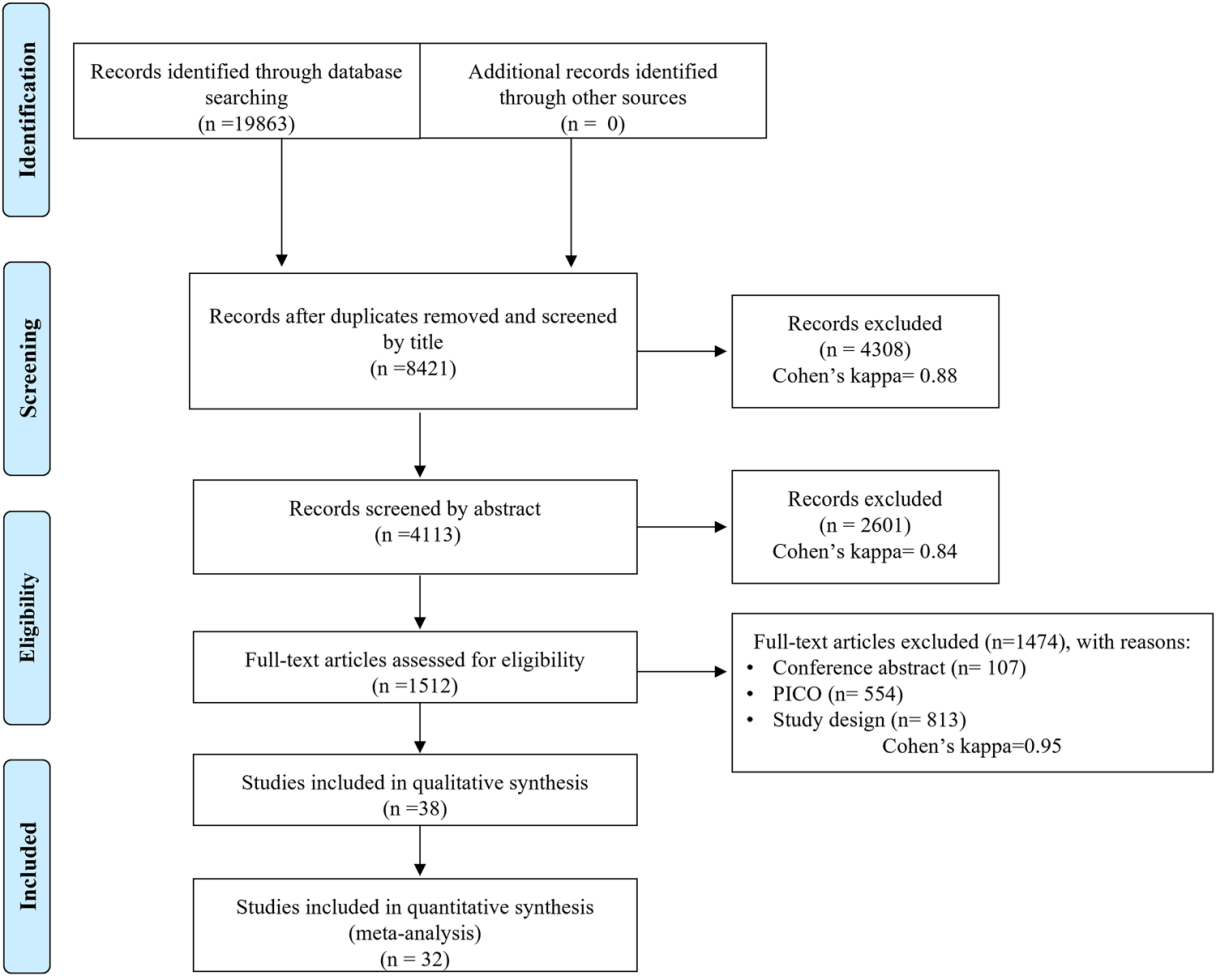
PRISMA flow diagram

A systematic literature search identified 19,863 articles. The results of the selection process and Cohen’s kappa coefficients are shown in detail in the PRISMA flowchart (Fig. 1). At the end of the selection process, we identified 38 eligible articles [14–19, 21, 31–61], of which 32 were included in the quantitative synthesis [14–19, 21, 31–35, 37, 39–44, 46, 47, 49, 50, 52–59, 61].

### Characteristics of the studies included

 The characteristics of the included studies are summarized in 3 tables, divided by the different approaches. Table 1, Supplementary Table 1 and 2 include routine IOC vs selective IOC, IOC vs no IOC, and selective IOC and no IOC, respectively. Eleven of the articles reported on both open and laparoscopic cholecystectomy [15–18, 21, 33–35, 37, 44, 54], whilst 27 of them covered laparoscopic cholecystectomy cases exclusively [14, 19, 31, 32, 36, 38–43, 45–53, 55–61]. We have summarized the different indications for selective IOC in Supplementary Table 3.

**Table 1:** Characteristics of included studies (routine IOC vs selective IOC)

Study	Study design	Centre(s)	Type of procedure	Comparison	Number of patients (female %, mean age ± SD)	Number of SIOC (n)	Outcomes	Follow-up
Alkhaffaf et al. 2011	Prospective cohort	Multicentric (4) in UK	LC	Routine IOC	463(80%, 47.8 ± 14.8)	-	BDI, conversion rate to open surgery, LOHS	N/A
				Selective IOC	1159(80%, 50.2 ± 15.7)	263		
Amott et al. 2005	Quasi-randomized trial	Single centre in Australia	LC	routine IOC	148	-	BDI, retained stone rate, success rate of IOC, operation time	N/A
				Selective IOC	155	45		
Buddingh et al. 2011	Retrospective cohort	Single centre in the Netherlands	Cholecystectomy	Routine IOC	435 (63.9%, 53 ± 17)	-	BDI, conversion rate to open surgery, success rate of IOC, operation time	N/A
				Selective IOC	421(64.4%, 53 ± 16)	25		
Carlson et al. 1993	Prospective cohort	Multicentric (2) in USA	LC	Routine IOC	164	-	BDI, retained stone rate	A inst: 9–28 months, B inst: 16–31 months
				Selective IOC	155	21		
Guerra-Filho et al. 2007	Prospective cohort	Single centre in Brazil	LC	Routine IOC	127(73.2%, 48.8)	-	Success rate of IOC	N/A
				Selective IOC	127(74%, 47.9)	71		
Nickkholgh et al. 2006	Retrospective cohort	Single centre in Iran	LC	Routine IOC	1133		BDI, retained stone rate, success rate of IOC	N/A
				Selective IOC	800	159		
Pham et al. 2016	Retrospective cohort	Multicentric (2) in China	LC	Routine IOC	246 (81%, 40, range: 33–57)		Retained stone rate, readmission rate, operation time	30-day
				Selective IOC	274 (76%, 44, range: 31–53)	15		
Ragulin-Coyne et al. 2013	Retrospective cohort	Multicentric (NIS) in USA	Cholecystectomy	Routine IOC	13,025 (66.9%, 53.5)	-	BDI, LOHS	N/A
				Selective IOC	98,790 (66%, 52.5)	N/A		
Snow et al. 2001	Retrospective cohort	Multicentric (4) in USA	LC	Routine IOC	1522	-	BDI, retained stone rate, success rate of IOC	11 year
				Selective IOC	487	139		

*IOC * intraoperative cholangiography,*LC * laparoscopic cholecystectomy, *BDI* bile duct injury, *LOHS * length of hospital stay

### Primary outcome(s)

#### Bile duct injury (BDI)

**Fig. 2:**
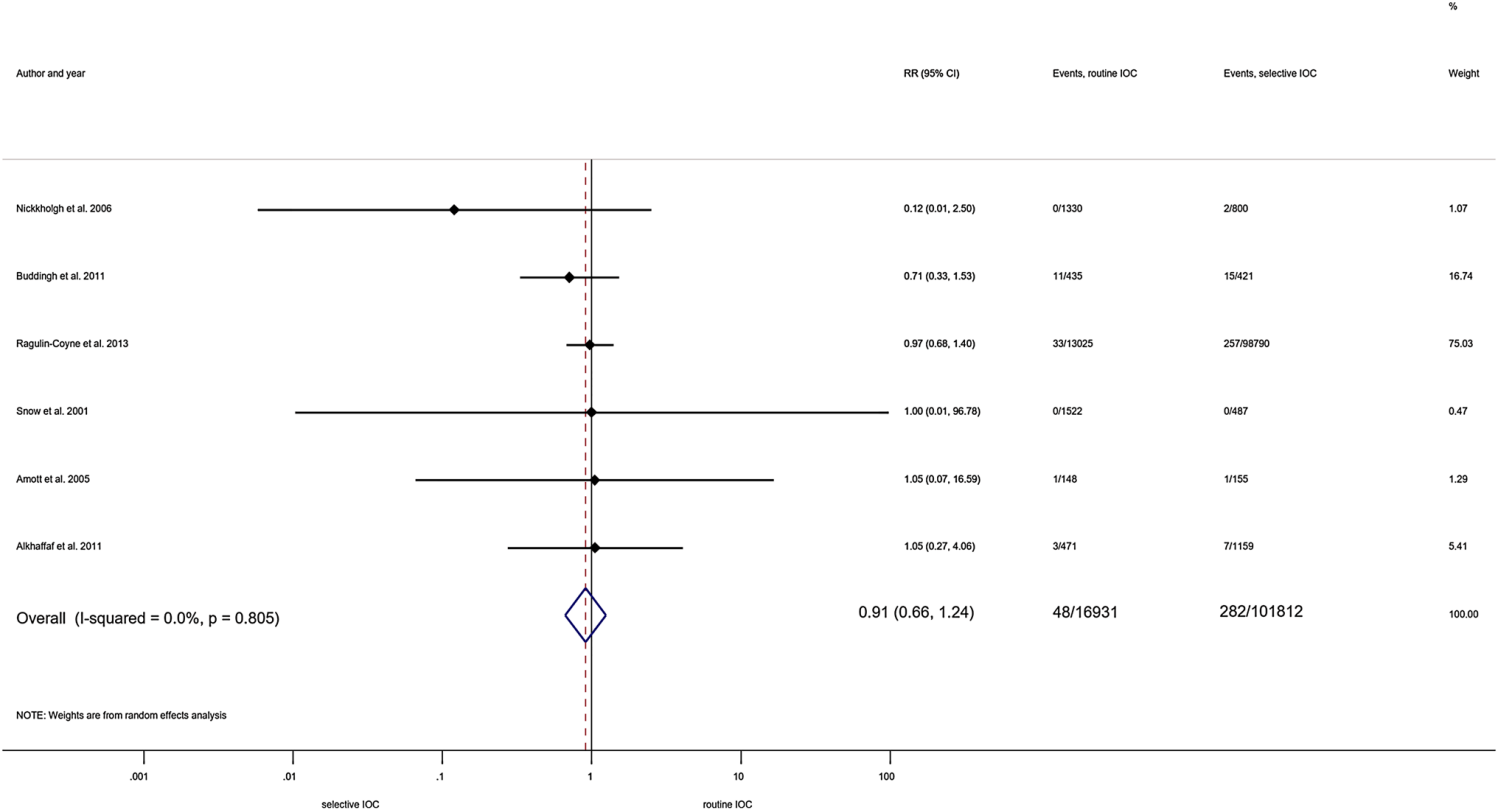
Forest plot comparing risk of BDI between routine IOC and selective IOC groups (population: both types of cholecystectomy). RR: relative risk; *p*: P value; CI confidence interval; I-squared: *I*^2^

*Routine IOC vs selective IOC.* We pooled six articles with 118,742 patients for this comparison [21, 31, 32, 35, 49, 55]. We detected no protective effect against BDI in either group (RR = 0.91, 95% CI 0.66; 1.24) along with statistically not significant heterogeneity (I^2^ = 0.0%, *p* = 0.805) (Fig. 2).

**Fig. 3:**
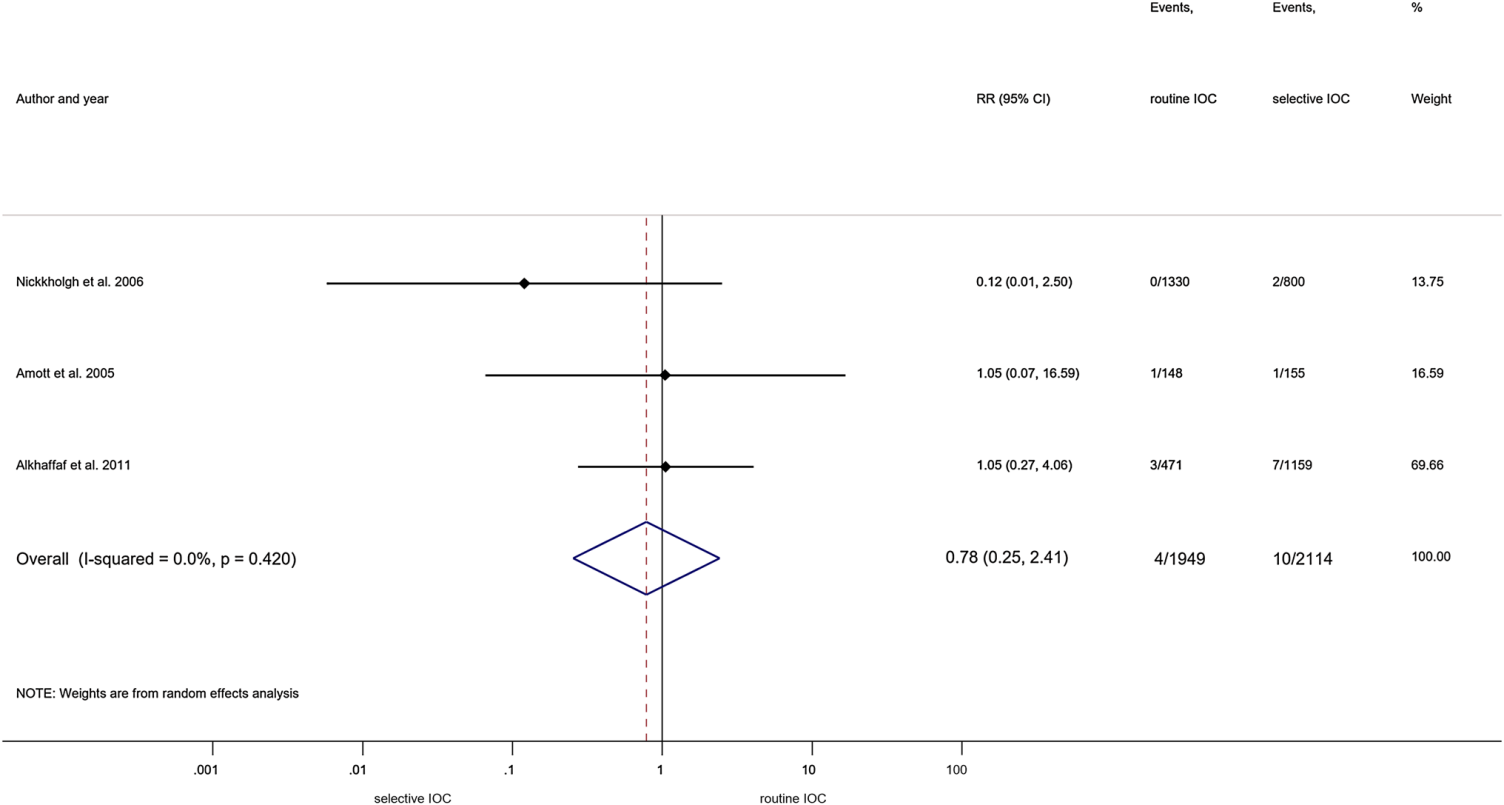
Forest plot comparing risk of BDI between routine IOC and selective IOC groups (population: laparoscopic cholecystectomy). RR: relative risk; *p*: P value; CI confidence interval; I-squared: *I*^2^

The lack of protective effect against BDI still remained after excluding the articles reporting on open cholecystectomy (RR = 0.78, 95% CI 0.25; 2.41) [31, 32, 49]. This analysis was carried out amongst articles with insignificant statistical heterogeneity (*I*^2^ = 0.0%, *p* = 0.420) (Fig. 3).

**Fig. 4:**
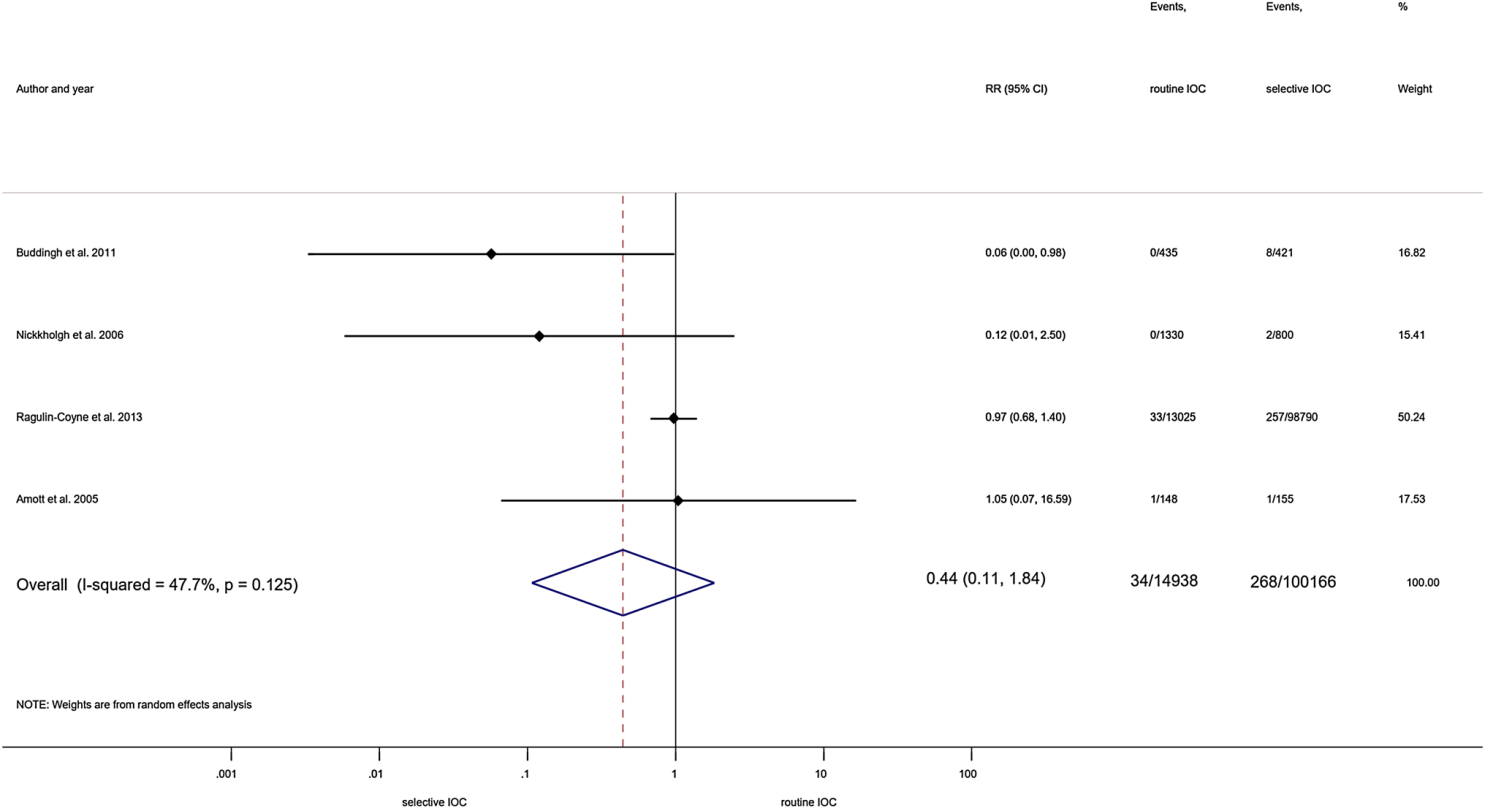
Forest plot comparing risk of MBDI between routine IOC and selective IOC groups (population: both types of cholecystectomy). RR: relative risk; p: P value; CI confidence interval; I-squared: I^2^

**Fig. 5:**
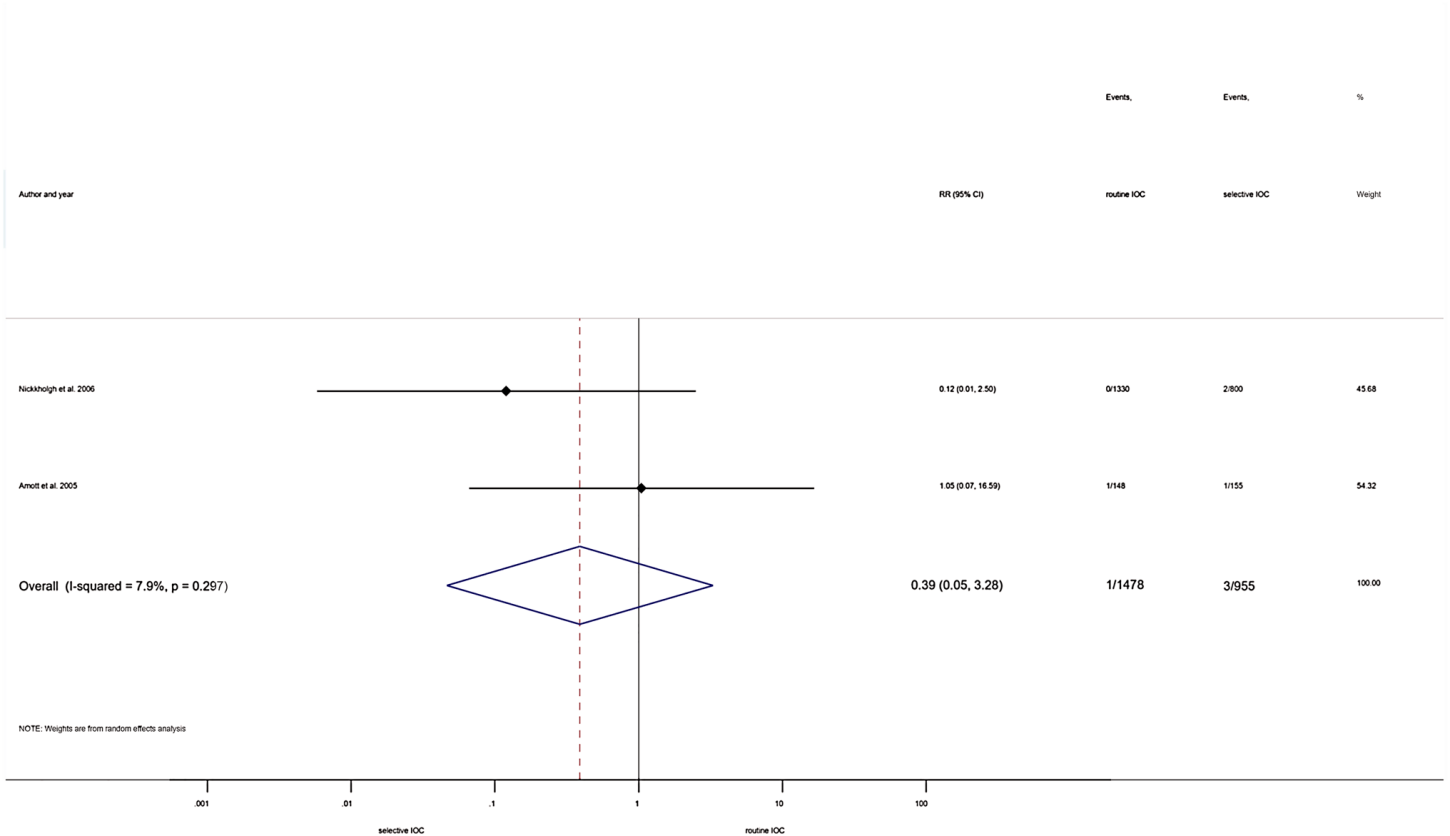
Forest plot comparing risk of MBDI between routine IOC and selective IOC groups (population: laparoscopic cholecystectomy). RR: relative risk; *p*: P value; CI confidence interval; I-squared: *I*^2^

In the same comparison, additional subgroup analyses were performed investigating MBDI. Investigating open and laparoscopic cholecystectomy cases, we found no differences between groups (RR = 0.44, 95% CI 0.11; 1.84; heterogeneity: *I*^2^ = 47.7%, *p* = 0.125) (Fig. 4) [21, 32, 35, 49]. Similarly, no difference was detected when including only laparoscopic cholecystectomy cases (RR = 0.39, 95% CI 0.05; 3.28; heterogeneity: *I*^2^ = 7.9%, *p* = 0.297) (Fig. 5) [32, 49].

*IOC vs no IOC.* Based on our analysis of 14 articles with 3,155,940 patients, the use of IOC was not associated with a reduced risk of BDI (RR = 1.03, 95% CI 0.77; 1.37) in substantially heterogeneous publications (*I*^2^ = 96.5%, *p* = 0.000) (Supplementary Fig. 1) [14–19, 34, 37, 40, 46, 47, 53, 56, 61].

The subgroup analysis of ten studies reporting on laparoscopic cholecystectomy exclusively found no difference between the two strategies with 706,336 patients included (RR = 1.19, 95% CI 0.79; 1.79); however, significant heterogeneity was identified (*I*^2^ = 82.4%, *p* = 0.000) (Supplementary Fig. 2) [14, 19, 37, 39, 40, 46, 47, 53, 56, 61].

We performed three additional subgroup analyses with only the prospective studies (RR = 1.09, 95% CI 0.77; 1.54; heterogeneity: *I*^2^ = 0.0%, *p* = 0.965) (Supplementary Fig. 3) [19, 40, 46, 56, 61] and all the studies reported on MBDI (RR = 1.01, 95% CI 0.70; 1.45; heterogeneity: *I*^2^ = 96.7%, *p* = 0.000) (Supplementary Fig. 4) [15, 16, 18, 19, 34, 46, 53, 56, 61], and then we pooled the studies with MBDI in LC only (RR = 1.09, 95% CI 0.35; 3.34; heterogeneity: *I*^2^ = 74.8%, *p* = 0.003) (Supplementary Fig. 5). [19, 39, 46, 53, 56] Neither of them found significant differences between the groups under investigation.

### Retained biliary stones after cholecystectomy

Comparing IOC and no IOC, five pooled studies with 2,069 cases found no difference (RR = 0.51, 95% CI 0.12; 2.11) within a one-year follow-up period, nor was statistically significant heterogeneity found (*I*^2^ = 13.7%, *p* = 0.327) (Supplementary Fig. 6) [19, 33, 56–58].

Despite our initial question, we could not examine the routine IOC vs selective IOC groups because follow-up periods were too variable. The results of these articles can be found in the supplementary material (Supplementary Table 4).

### Secondary outcome(s)

#### Routine vs selective IOC

When analysing the success rate of IOC during laparoscopic cholecystectomy in the four studies involved comparing routine IOC and selective IOC, we were not able to identify any statistically significant difference (RR = 0.96, 95% CI 0.86; 1.06; *I*^2^ = 88.2%, *p* < 0.001) (Supplementary Fig. 7) [32, 41, 49, 55].

Comparing the routine and selective approach by operation time, the results did not show us a statistically significant difference (WMD = 14.02, 95% CI –6.96; 35.00, *I*^2^ = 98.2%, *p* < 0.001), including three studies with 2445 patients. These studies only investigated patients who had undergone laparoscopic cholecystectomy (Supplementary Fig. 8) [31, 32, 50].

### IOC vs no IOC

The meta-analysis of three studies with 10,735 patients identified a significant difference (RR = 0.64, 95% CI 0.51; 0.78) favouring IOC with a lower risk of conversion to open surgery compared to no IOC group without significant heterogeneity (*I*^2^ = 0.4%, *p* = 0.336) (Supplementary Fig. 9) [19, 46, 61]. The operation time was significantly longer during cholecystectomy in the IOC group compared to the no IOC group (WMD = 11.25 min, 95% CI 6.57; 15.93; heterogeneity *I*^2^ = 95.9%, *p* = 0.000) (Supplementary Fig. 10) [19, 33, 42, 46, 49, 56, 59] .

The meta-analysis comparing the readmission rate after laparoscopic cholecystectomy between IOC and no IOC groups within a follow-up period of 30 days found no statistically significant difference (RR = 0.92, 95% CI 0.79; 1.06, *I*^2^ = 86.9%, *p* < 0.001) (Supplementary Fig. 11) [14, 42, 52, 56]. Comparing IOC and no IOC by length of hospital stay, no statistically significant differences were found (WMD = -0.03, 95% CI –0.26; 0.20; heterogeneity: *I*^2^ = 98.3%, *p* < 0.001) (Supplementary Fig. 12) [14, 19, 33, 43, 44, 54, 56, 59]. The results were the same when we analysed studies reporting exclusively on LC cases (WMD = 0.04, 95% CI –0.12; 0.19; heterogeneity: *I*^2^ = 90.0%, *p* < 0.001) (Supplementary Fig. 13) [19, 43, 56, 59].

### Qualitative synthesis

We included the following endpoints in our qualitative synthesis: BDI, MBDI (routine IOC vs selective IOC: one publication [36]; IOC vs no IOC: one publication) [45], retained stone rate (routine IOC vs selective IOC: five studies [32, 36, 49, 50, 55]; IOC vs no IOC: one publication [38]; selective IOC vs no IOC: three studies) [48, 51, 60], readmission rate (IOC vs no IOC: four studies) [33, 46, 57, 58], conversion rate to open surgery (routine IOC vs selective IOC: two studies) [31, 35], success rate of IOC (routine IOC vs selective IOC: one study) [35], operation time (routine IOC vs selective IOC: one study [35]; IOC vs no IOC: one study) [38] and length of hospital stay (IOC vs no IOC: one study [57]; routine IOC vs selective IOC: three studies) [21, 31, 50]. A summary of studies only included in the qualitative synthesis can be found in the supplementary material (Supplementary Tables 4 and 5).

### Publication bias and risk of bias assessment

Based on a visual assessment of funnel plots, there is a high risk of publication bias in the case of MBDI when the population consisted of both types of cholecystectomy and when only LCs were performed, retained biliary stones after cholecystectomy, operation time (population consisted of LC; comparison: IOC vs no IOC), operation time (population consisted of LC, comparison: IOC vs no IOC), and length of hospital stay (population consisted of both types of cholecystectomy; comparison: IOC vs no IOC). The results of publication bias, funnel plots, and Egger’s tests can be found in the supplementary data.

Most of the publications investigated were deemed to have a serious risk of bias because of the presence of uncontrolled confounding factors. We excluded three articles from the quantitative synthesis due to critical risk of bias [36, 38, 45]. A summary of the risk of bias assessment can be found in the supplementary data.

### Certainty of evidence

All examined outcomes were assessed as having a very low level of evidence. The design of the included studies, the potential presence of uncontrolled confounding factors, the significant level of heterogeneity greatly influenced the quality of evidence. The GRADE evidence profile tables are shown in Tables 2 and 3 summarizing the comparison of routine IOC vs selective IOC and in Supplementary Table 6 and 7 concerning about IOC vs no IOC.

**Table 2:** GRADE evidence profile – Comparison: Routine vs selective IOC. Population: both type of cholecystectomy

Certainty assessment							№ of patients		Effect		Certainty	Importance
№ of studies	Study design	Risk of bias	Inconsistency	Indirectness	Imprecision	Other considerations	Routine IOC	selective IOC	Relative (95% CI)	Absolute (95% CI)		
*Bile duct injury (both type of cholecystectomy) (assessed with: RR)*												
6	observational studies	very serious^a^	not serious	serious^b^	not serious	none	48/16930 (0.3%)	282/101812 (0.3%)	RR 0.91 (0.66 to 1.24)	0 fewer per 1 000 (from 1 fewer to 1 more)	⨁◯◯◯ Very low	CRITICAL
*Major bile duct injury (both type of cholecystectomy) (assessed with: RR)*												
*4*	observational studies	very serious^a^	not serious	serious^b^	not serious	publication bias strongly suspected^c^	34/14938 (0.2%)	268/100166 (0.3%)	RR 0.44 (0.11 to 1.84)	1 fewer per 1 000 (from 2 fewer to 2 more)	⨁◯◯◯ Very low	CRITICAL

*CI* confidence interval, *RR* risk ratio

^a^Bias is likely due to the presence of confounding factors

^b^Most patients had acute biliary disease

^c^Due to the low number of articles publication bias was not assessed

**Table 3:** GRADE evidence profile – Comparison: Routine vs selective IOC. Population: laparoscopic cholecystectomy

Certainty assessment							№ of patients		Effect		Certainty	Importance
№ of studies	Study design	Risk of bias	Inconsistency	Indirectness	Imprecision	Other considerations	Routine IOC	selective IOC	Relative (95% CI)	Absolute (95% CI)		
*Bile duct injury (laparoscopic cholecystectomy) (assessed with: RR)*												
3	observational studies	very serious^a^	not serious	not serious	not serious	publication bias strongly suspected^b^	4/1949 (0.2%)	10/2114 (0.5%)	RR 0.78 (0.25 to 2.41)	1 fewer per 1 000 (from 4 fewer to 7 more)	⨁◯◯◯ Very low	CRITICAL
*Major bile duct injury (laparoscopic cholecystectomy) (assessed with: RR)*												
2	observational studies	very serious^a^	not serious	not serious	not serious	publication bias strongly suspected^b^	1/1478 (0.1%)	3/955 (0.3%)	RR 0.39 (0.05 to 3.28)	2 fewer per 1 000 (from 3 fewer to 7 more)	⨁◯◯◯ Very low	CRITICAL
*Success rate of IOC (laparoscopic cholecystectomy) (assessed with: RR)*												
4	observational studies	very serious^a^	very serious^c^	not serious	very serious^d^	publication bias strongly suspected^e^	2846/3127 (91.0%)	372/414 (89.9%)	RR 0.96 (0.86 to 1.06)	36 fewer per 1 000 (from 126 fewer to 54 more)	⨁◯◯◯ Very low	IMPORTANT
*Operation time (laparoscopic cholecystectomy) (assessed with: WMD)*												
3	observational studies	very serious^a^	very serious^c^	serious^f^	serious^g^	publication bias strongly suspected^h^	857	1588	-	WMD 14.02 min. more (6.96 fewer to 35 more)	⨁◯◯◯ Very low	IMPORTANT

*CI* confidence interval, *RR* risk ratio

^a^Bias is likely due to the presence of confounding factors

^b^Due to the low number of articles publication bias was not assessed

^c^Inconsistency is likely due to the presence of statistically significant heterogeneity

^d^Confidence intervals cross the benefit/harm line and 0−effect line

^e^Publication bias is likely due to funnel plot asymmetry

^f^Indirect population is likely due to the variable inclusion and exclusion criteria

^g^Imprecision is likely due to the overall effect estimate lies between benefit and harm

^h^The risk of publication bias was not assessed due to the low number of studies included

### Discussion

Our results suggest that selective IOC may not be inferior to routine IOC in the prevention of BDI. The success rate of IOC and the operation time were also similar between these two groups.

Carrying out IOC did not result in significant difference in any of the endpoints under examination compared to the omission of IOC, apart from a lower conversion rate and longer operation time. A significantly higher conversion rate to open surgery appeared in the no IOC group, and a significantly longer operation time was characteristic in the IOC group.

There is a consensus that IOC has its place in surgical practice due to its role in detecting CBD stones and diagnosing BDI. However, the recommendations are not unanimous because there are still doubts about the extent to which IOC can prevent BDI [4–13]. According to the latest meta-analysis published in 2021, IOC should be performed routinely, it has a protective effect against BDI over the selective approach, and it is a cost-effective intervention as well [12]. In contrast, others suggest liberal [9] or selective [5] use of IOC to mitigate the risk of BDI.

### Primary outcome(s)

#### Bile duct injury (BDI)

Our findings do not support the higher protective value of routine IOC compared to selective cholangiography. In addition, our results indicate that IOC has no clear benefit over omission of IOC; a selective policy might be more reasonable rather than the omission of IOC.

Based on our results, the role of intraoperative cholangiography in preventing BDI can be questioned. However, it still plays an important diagnostic role for BDI and CBD stones [40, 54]. From our standpoint, the main question is not whether an IOC should be performed but in whom: whether it should be done in all cases or only when the situation requires it.

Articles concentrating on routine IOC vs selective IOC draw different conclusions. The latest one was published in 2013 by Ragulin-Coyne et al.; it involved 111,815 patients and found that routine IOC does not reduce the rate of BDI but incurs a high cost [21]. In contrast, Buddingh et al., concluded that implementation of routine IOC had a protective effect against major BDI [35]. As mentioned earlier, in a meta-analysis published in 2021 the authors claim that routine IOC decreases the risk and prevents BDI, and is also more cost-effective [12]. However, these results should be handled with caution because the population defined as selective IOC in the meta-analysis appears as patients “without IOC” in the majority of the pooled studies [15, 37, 39, 40, 54, 62].

Sheffield et al. have shown that the link between IOC and common BDI may be due to unmeasured confounding factors [54] and differences in baseline characteristics between the comparator groups. They claim that the relation between IOC and common BDI is sensitive to the statistical method applied [54]. They used the standard risk adjustment method to determine that omission of IOC is linked to BDI, even after controlling for potential confounding factors. At the same time, when instrumental variable methods were applied, the association was no longer significant.

In comparing IOC and no IOC, several publications used data obtained from large databases [14–16, 18, 39, 47, 53]. They frequently used a proxy definition of BDI because these databases have no precise definition of BDI. Lilley et al. claim that the use of a proxy definition for common BDI is not an appropriate method to identify it because this may introduce confounding by indication [16].

### Retained biliary stones after cholecystectomy

Our results suggest that IOC does not significantly reduce the rate of postoperatively detected residual CBD stones.

Hope et al. state that IOC should be used freely to detect CBD stones as decided by the surgeon due to its high sensitivity, specificity, positive and negative predictive value, and accuracy [9]. Another meta-analysis suggests that IOC should be used more widely in the diagnosis and management of CBD stones [7]. A previous meta-analysis published in 2012 states that routine IOC can reduce the readmission for retained CBD stones; however, they do not suggest routine IOC in patients without suspicion of CBD stones (based on clinical, biochemical, or radiological findings) [13]. A recent retrospective study found that asymptomatic untreated bile duct stones have a cumulative incidence of biliary complications (6.1% at one year, 11% at three years and 17% at five years). Still, they concluded that doing follow-ups for patients with in situ stones (a “wait-and-see strategy”) is more beneficial than early endoscopic management of asymptomatic stones [63]. A study published in 2013 points out that routine IOC may detect more CBD stones intraoperatively than ideal [21]. The number of endoscopic retrograde cholangiopancreatographies (ERCP) and CBD explorations is thus higher in the case of routine IOC users. They also found a link between the use of routine IOC and an increased overall complication rate. They support the idea of selective IOC because it can reduce the number of unnecessary interventions. Sheffield et al. confirm this; they also found relationship between routine IOC and higher use of ERCP and common duct exploration [54]. According to the ASGE (American Society of Gastrointestinal Endoscopy) guideline, the number of diagnostic ERCPs should be reduced because of their high risk and lack of benefit [64].

The ASGE and ESGE (European Society of Gastrointestinal Endoscopy) recommend two similar pre-operative risk stratification methods to assess the probability of CBD stones [64, 65]. These guidelines might be helpful in terms of indexing patients who need further investigation and possible management of CBD stones. These algorithms allow the surgeon to consider whether an IOC is required or not.

### Secondary outcome(s)

When we compared routine to selective IOC, we did not find a significant difference between them as regards the success rate of IOC and operation time.

A significant difference was found between IOC and no IOC in conversion rate to open surgery and operation time. The results indicate a higher conversion rate to open surgery amongst patients who did not receive IOC. Conversion to open surgery frequently happens during a difficult laparoscopic dissection or when BDI is suspected [47]. A previous publication reports that a higher risk of BDI appears with the conversion from a laparoscopic to an open procedure [66]. This could reflect the surgeon’s lack of experience with open cholecystectomy [67] or the overall difficulty of the case [47].

The data suggest that patients who underwent LC and IOC had a significantly longer operation time by almost 13 min. This result contradicts the theory put forth by opponents of IOC: IOC adds a significant amount of time to LC.

Investigating readmission rate and length of hospital stay between IOC and no IOC, we found no significant differences.

### Strengths and limitations

Our meta-analysis is characterized by comprehensiveness and a high number of included patients. Compared to the recently published meta-analyses [7, 12] we placed great emphasis on the study of routine IOC vs selective IOC approaches and identified several additional articles [34, 36, 37, 45, 47, 53, 55, 56, 61]. We performed several subgroup analyses (only LC cases exclusively, MBDI and prospective studies) to provide a better quality of evidence and a more thorough review.

Our study has some limitations. The majority of the pooled articles are retrospective cohort studies. They provide data from large-scale databases with potential sources of bias and are not controlled or partially adjusted for confounding variables. Our results should therefore be handled with caution.

Our results continue to lose strength due to the presence of statistical heterogeneity for some endpoints. This phenomenon might be the result of study design, not only laparoscopic cholecystectomy included, variable use of IOC, different definitions of BDI, and varying degrees of confounding factor control. In addition, IOC is used as a diagnostic tool for detecting BDI in some cases, which has a potential distortive effect.

### Implications for practice

A selective approach to IOC may be appropriate. Selective IOC combined with measures that aid prevention of BDI (e.g. critical view of safety, fundus-first approach, multi-port laparoscopic technique, and low threshold for conversion to open cholecystectomy) [5, 68] and together with investigations that detect bile duct stone perioperatively (e.g. abdominal ultrasound, laparoscopic ultrasound, endoscopic ultrasound, and magnetic resonance cholangiopancreatography) [5, 64] should be considered.

### Implications for research

No uniform indication system for selective IOC has been developed. Such an indication system should consider the risk factors for BDI (e.g. sex, age, experience of surgeons, prolonged laparoscopic cholecystectomy, and indication for cholecystectomy) and the possible presence (based on clinical, laboratory, and imaging findings) and available treatment of biliary stones. Future studies are needed to establish a standard indication system based on which surgeons perform IOC.

Several authors state that BDI cannot be examined with randomized trials due to its low incidence [8, 15]; high-quality prospective studies which also consider the presence of potential biases and confounding factors are therefore needed.

In conclusion, selective IOC may not be inferior to routine IOC in preventing BDI. IOC might not be indicated in every case, and its selective use may stand as an alternative to routine policy, however, the evidence is very uncertain. Further good quality research is required to address this question and to determine the exact selection criteria for IOC.

## Supplementary Information

Below is the link to the electronic supplementary material.

Supplementary file1 (TIF 975 KB)

Supplementary file2 (TIF 765 KB)

Supplementary file3 (TIF 590 KB)

Supplementary file4 (TIF 638 KB)

Supplementary file5 (TIF 656 KB)

Supplementary file6 (TIF 648 KB)

Supplementary file7 (TIF 750 KB)

Supplementary file8 (TIF 636 KB)

Supplementary file9 (TIF 664 KB)

Supplementary file10 (TIF 671 KB)

Supplementary file11 (TIF 750 KB)

Supplementary file12 (TIF 1102 KB)

Supplementary file13 (TIF 731 KB)

Supplementary file14 (TIF 2165 KB)

Supplementary file15 (TIF 2549 KB)

Supplementary file16 (TIF 2512 KB)

Supplementary file17 (TIF 2049 KB)

Supplementary file18 (TIF 1519 KB)

Supplementary file19 (TIF 2199 KB)

Supplementary file20 (TIF 1543 KB)

Supplementary file21 (TIF 1129 KB)

Supplementary file22 (TIF 503 KB)

Supplementary file23 (TIF 724 KB)

Supplementary file24 (TIF 478 KB)

Supplementary file25 (TIF 700 KB)

Supplementary file26 (TIF 1075 KB)

Supplementary file27 (TIF 477 KB)

Supplementary file28 (TIF 2700 KB)

Supplementary file29 (TIF 503 KB)

Supplementary file30 (TIF 1020 KB)

Supplementary file31 (TIF 419 KB)

Supplementary file32 (TIF 1147 KB)

Supplementary file33 (TIF 490 KB)

Supplementary file34 (TIF 856 KB)

Supplementary file35 (TIF 411 KB)

Supplementary file36 (TIF 1180 KB)

Supplementary file37 (TIF 482 KB)

Supplementary file38 (TIF 695 KB)

Supplementary file39 (TIF 964 KB)

Supplementary file40 (TIF 868 KB)

Supplementary file41 (TIF 416 KB)

Supplementary file42 (TIF 1138 KB)

Supplementary file43 (TIF 483 KB)

Supplementary file44 (TIF 1016 KB)

Supplementary file45 (TIF 419 KB)

Supplementary file46 (TIF 1162 KB)

Supplementary file47 (TIF 482 KB)

Supplementary file48 (TIF 856 KB)

Supplementary file49 (TIF 411 KB)

Supplementary file50 (DOCX 26 KB)

Supplementary file51 (DOCX 17 KB)

Supplementary file52 (DOCX 18 KB)

Supplementary file53 (DOCX 21 KB)

Supplementary file54 (DOCX 22 KB)

Supplementary file55 (DOCX 16 KB)

Supplementary file56 (DOCX 20 KB)

Supplementary file57 (PDF 89 KB)

## Abbreviations

ASGE: American Society of Gastrointestinal Endoscopy
BDI: Bile duct injury
CBD: Common bile duct
CI: 95% Confidence interval
ERCP: Endoscopic retrograde cholangiopancreatography
ESGE: European Society of Gastrointestinal Endoscopy
IOC: Intraoperative cholangiography
LC: Laparoscopic cholecystectomy
MBDI: Major bile duct injury
RCT: Randomized clinical trial
RR: Relative risk
WMD: Weighted mean difference
