# Supplementary figures and images for: Selective intraoperative cholangiography should be considered over routine intraoperative cholangiography during cholecystectomy: a systematic review and meta-analysis
Source: Surg Endosc. 2022 Jul 7;36(10):7126–39. doi: 10.1007/s00464-022-09267-x (PMC9485186; doi:10.1007/s00464-022-09267-x)

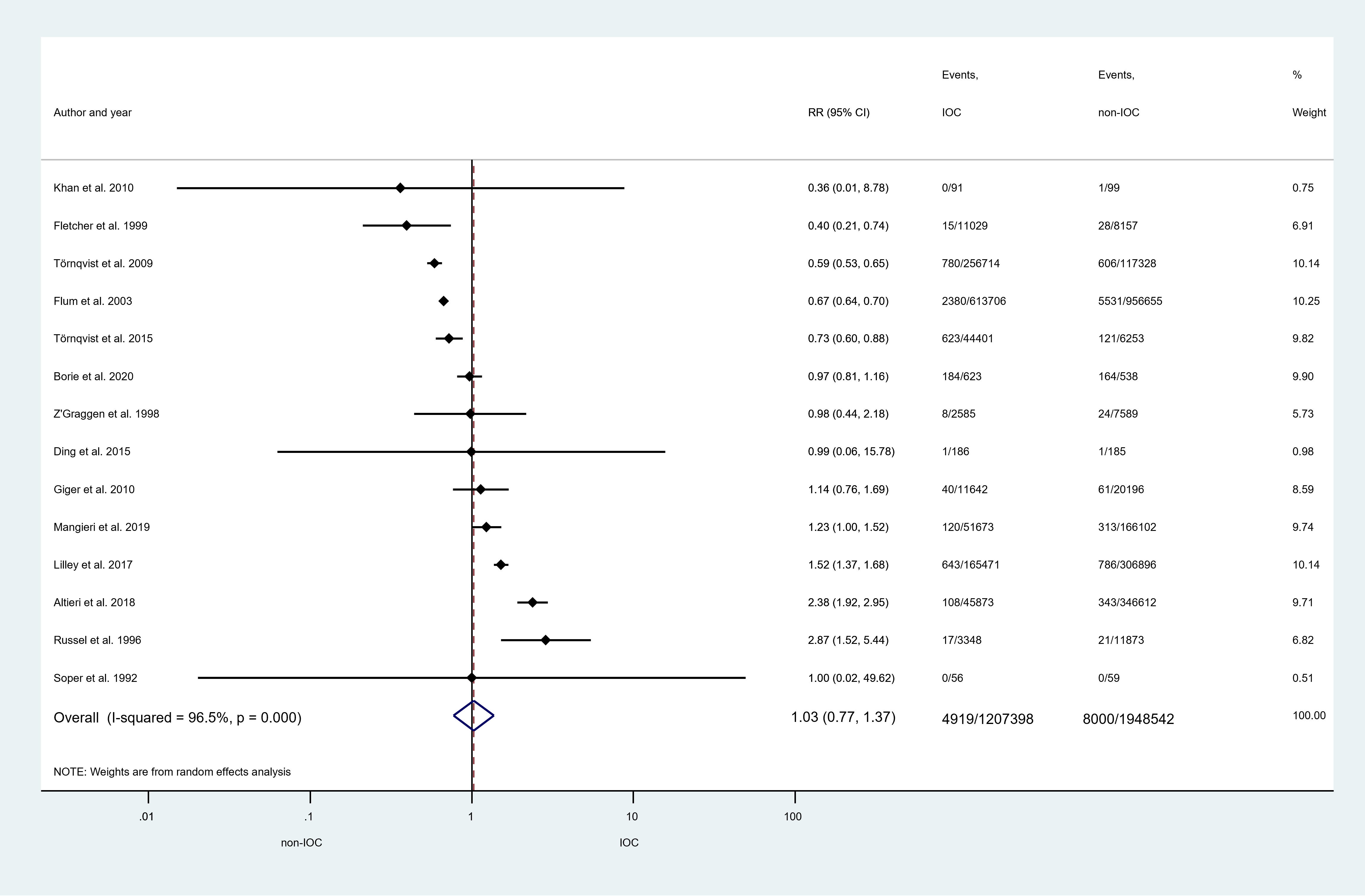

Supplement: Supplementary file 1 — Supplementary file1 (TIF 975 KB) [file 464_2022_9267_MOESM1_ESM.tif]

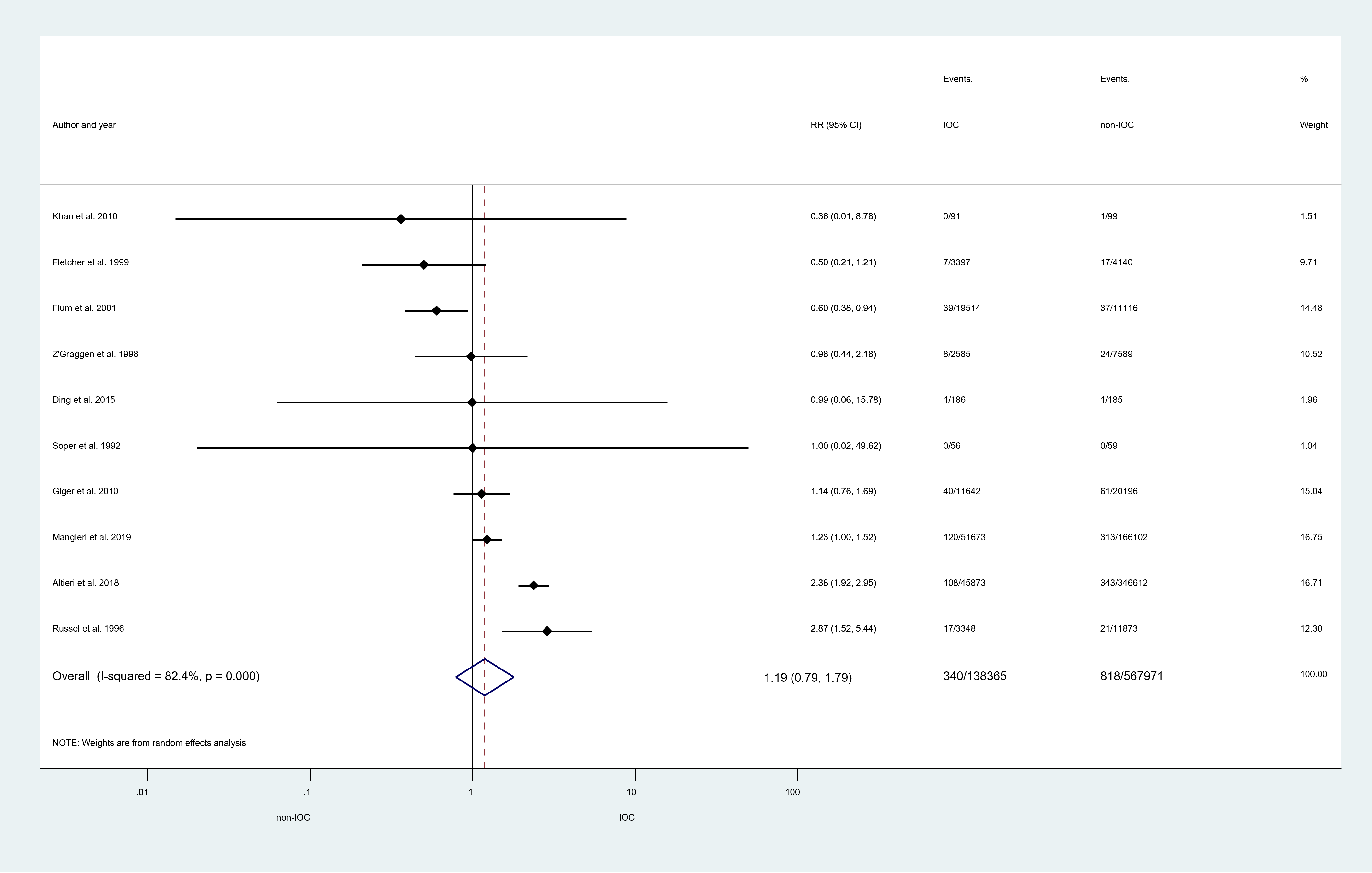

Supplement: Supplementary file 2 — Supplementary file2 (TIF 765 KB) [file 464_2022_9267_MOESM2_ESM.tif]

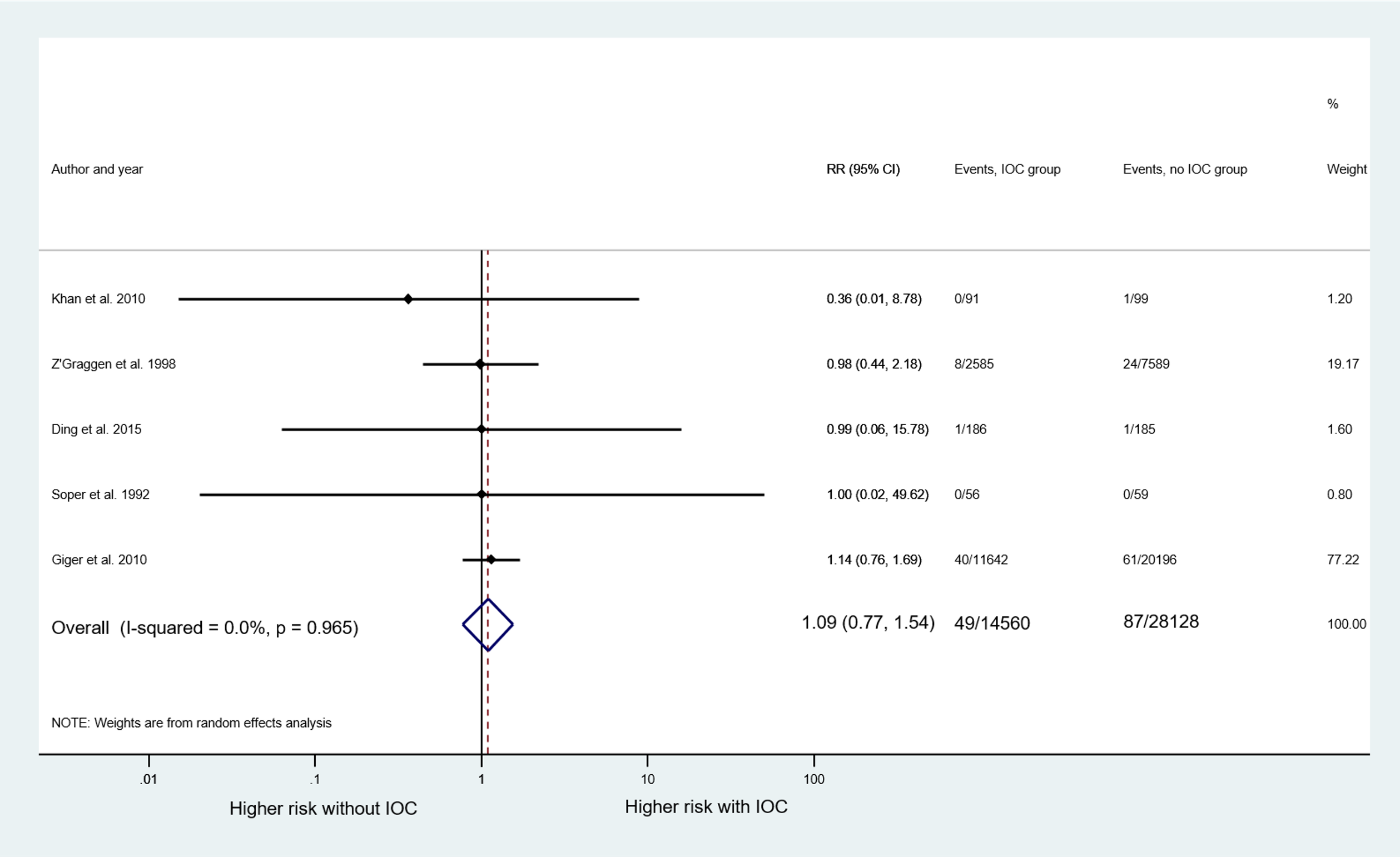

Supplement: Supplementary file 3 — Supplementary file3 (TIF 590 KB) [file 464_2022_9267_MOESM3_ESM.tif]

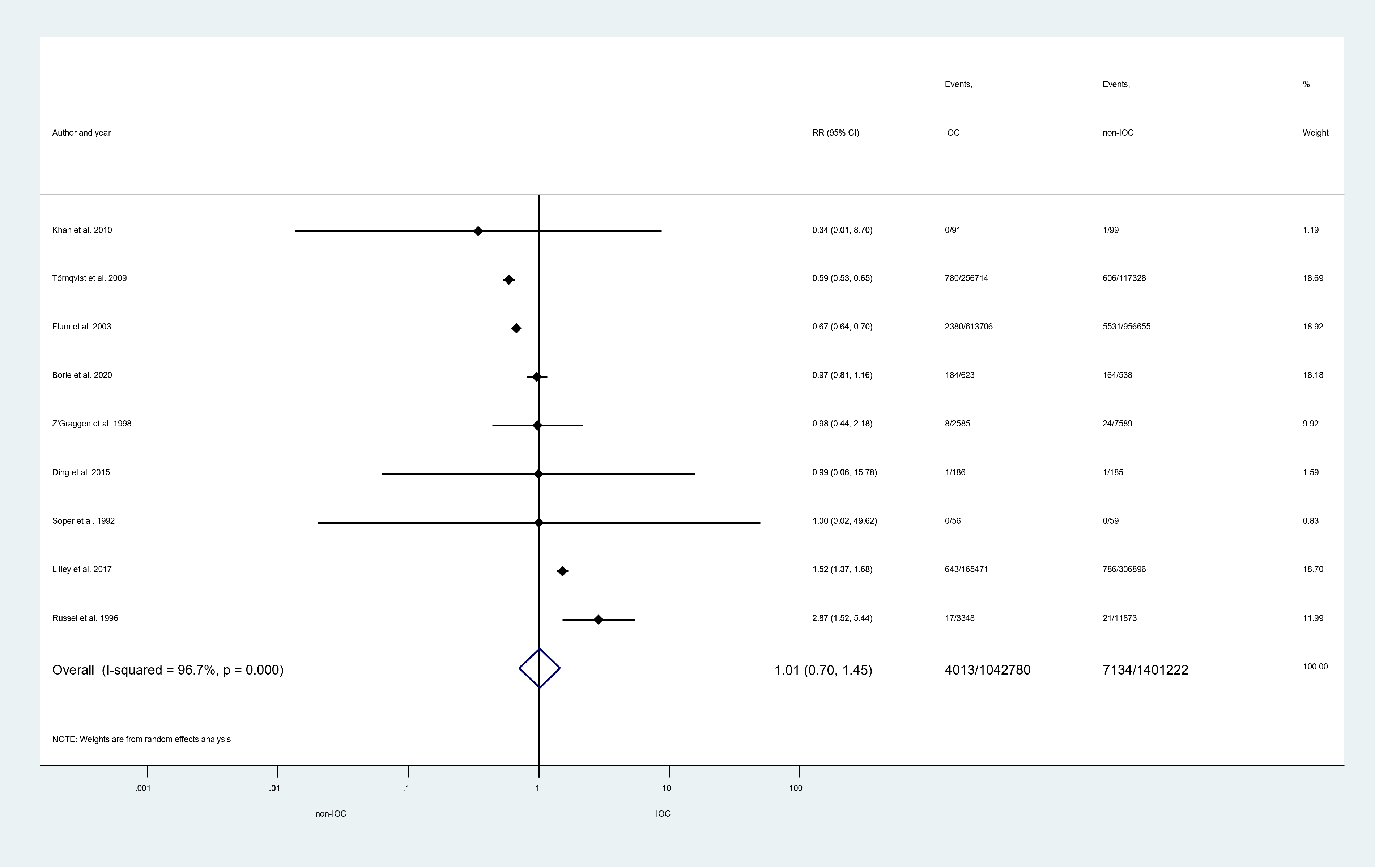

Supplement: Supplementary file 4 — Supplementary file4 (TIF 638 KB) [file 464_2022_9267_MOESM4_ESM.tif]

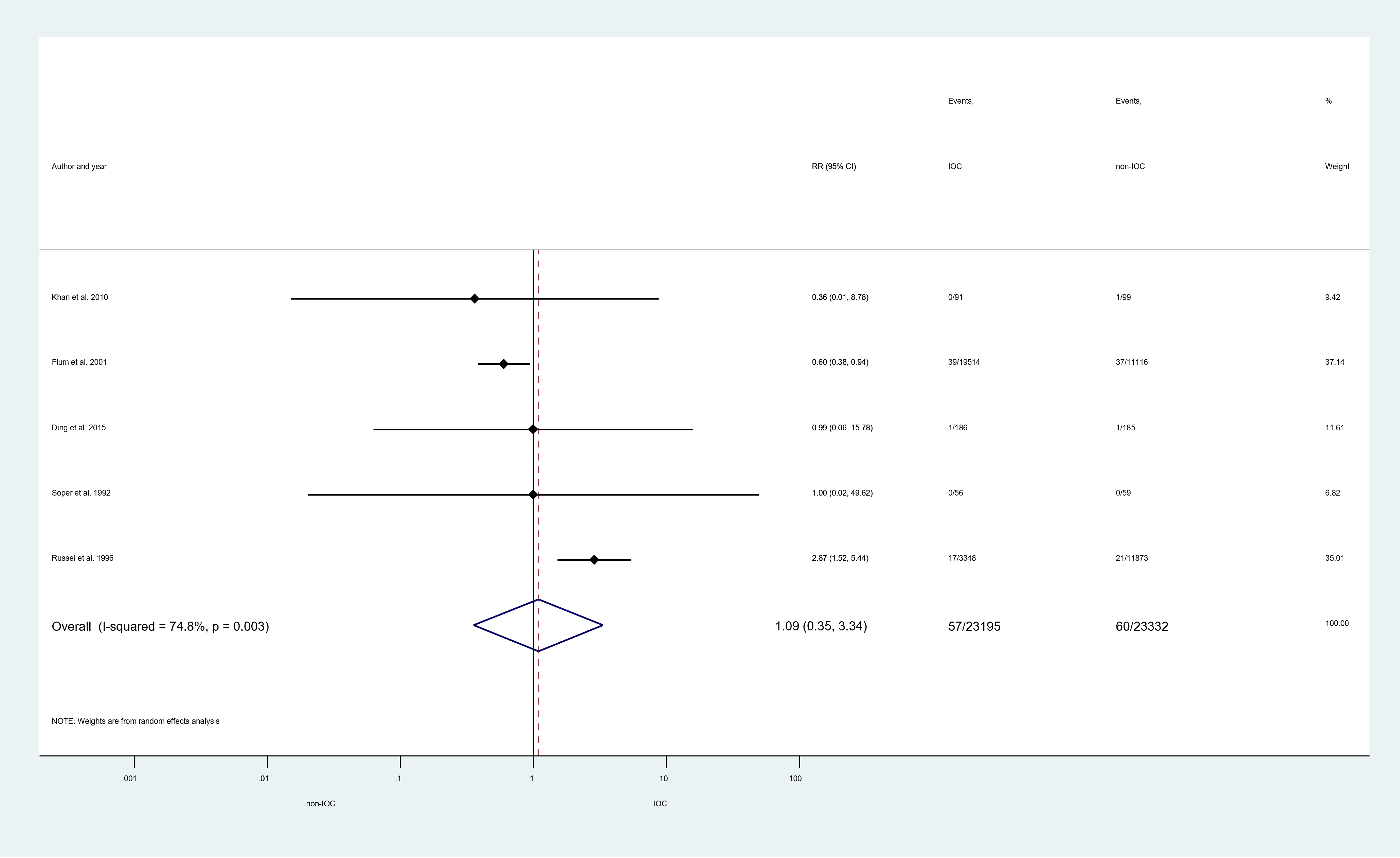

Supplement: Supplementary file 5 — Supplementary file5 (TIF 656 KB) [file 464_2022_9267_MOESM5_ESM.tif]

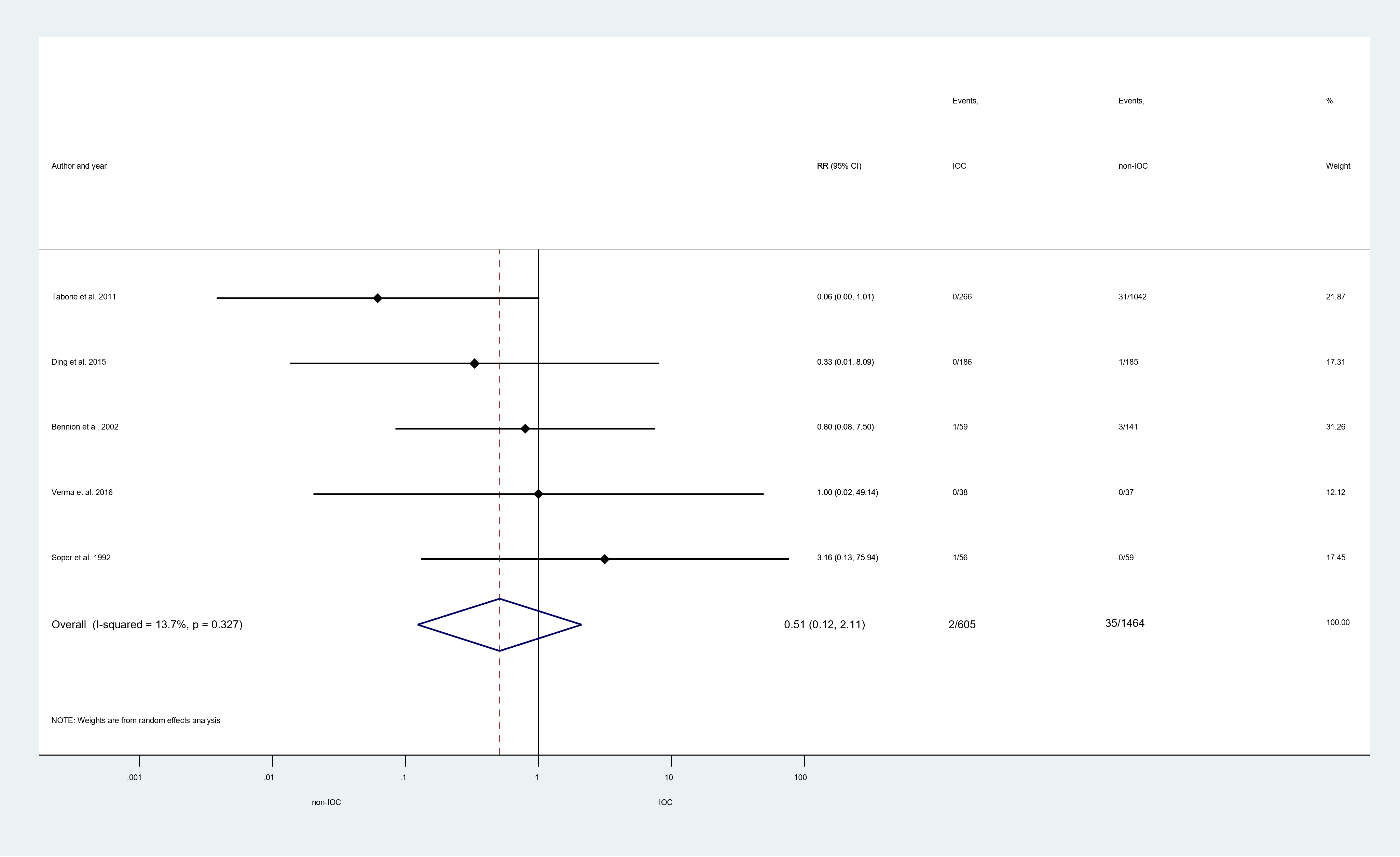

Supplement: Supplementary file 6 — Supplementary file6 (TIF 648 KB) [file 464_2022_9267_MOESM6_ESM.tif]

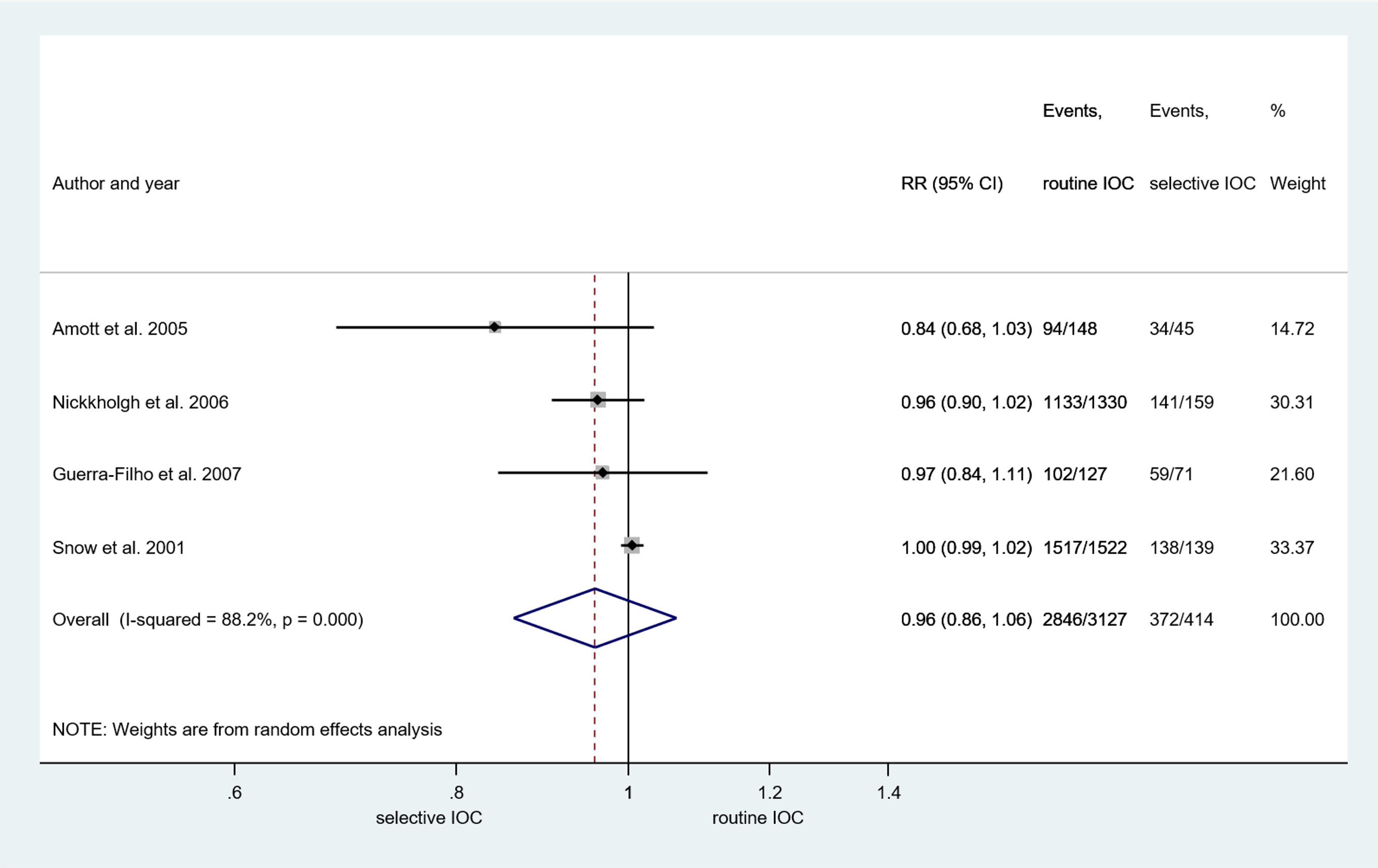

Supplement: Supplementary file 7 — Supplementary file7 (TIF 750 KB) [file 464_2022_9267_MOESM7_ESM.tif]

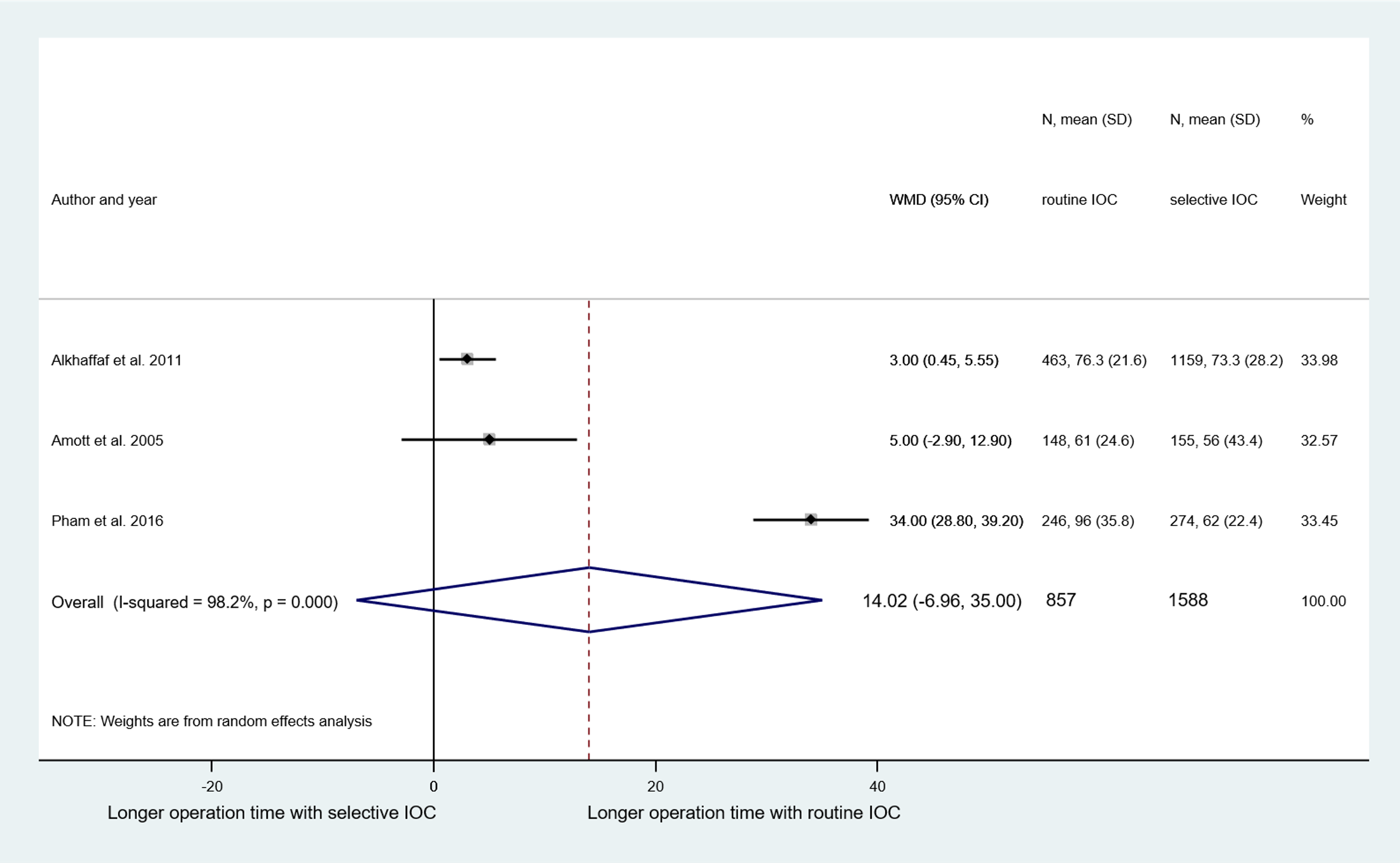

Supplement: Supplementary file 8 — Supplementary file8 (TIF 636 KB) [file 464_2022_9267_MOESM8_ESM.tif]

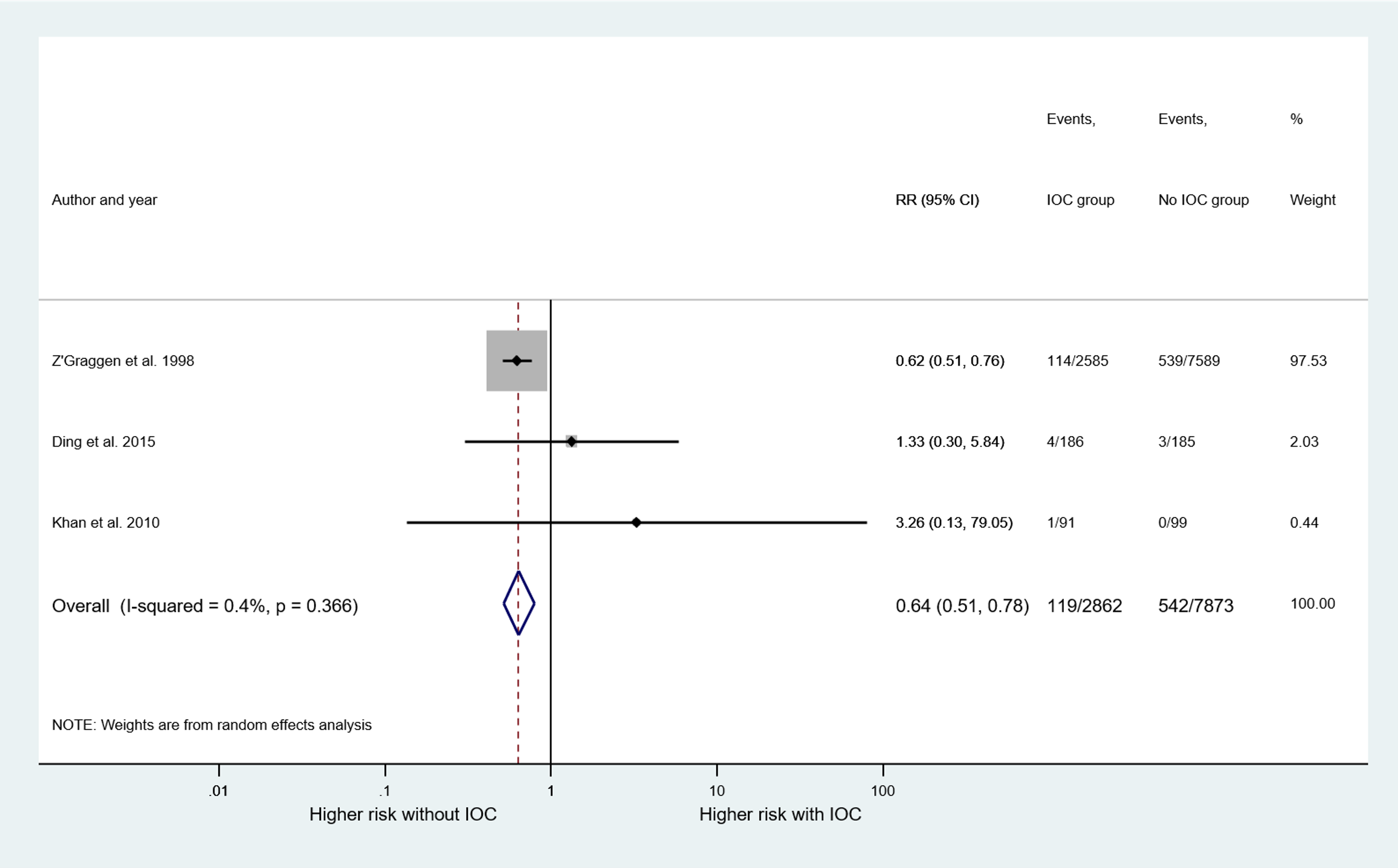

Supplement: Supplementary file 9 — Supplementary file9 (TIF 664 KB) [file 464_2022_9267_MOESM9_ESM.tif]

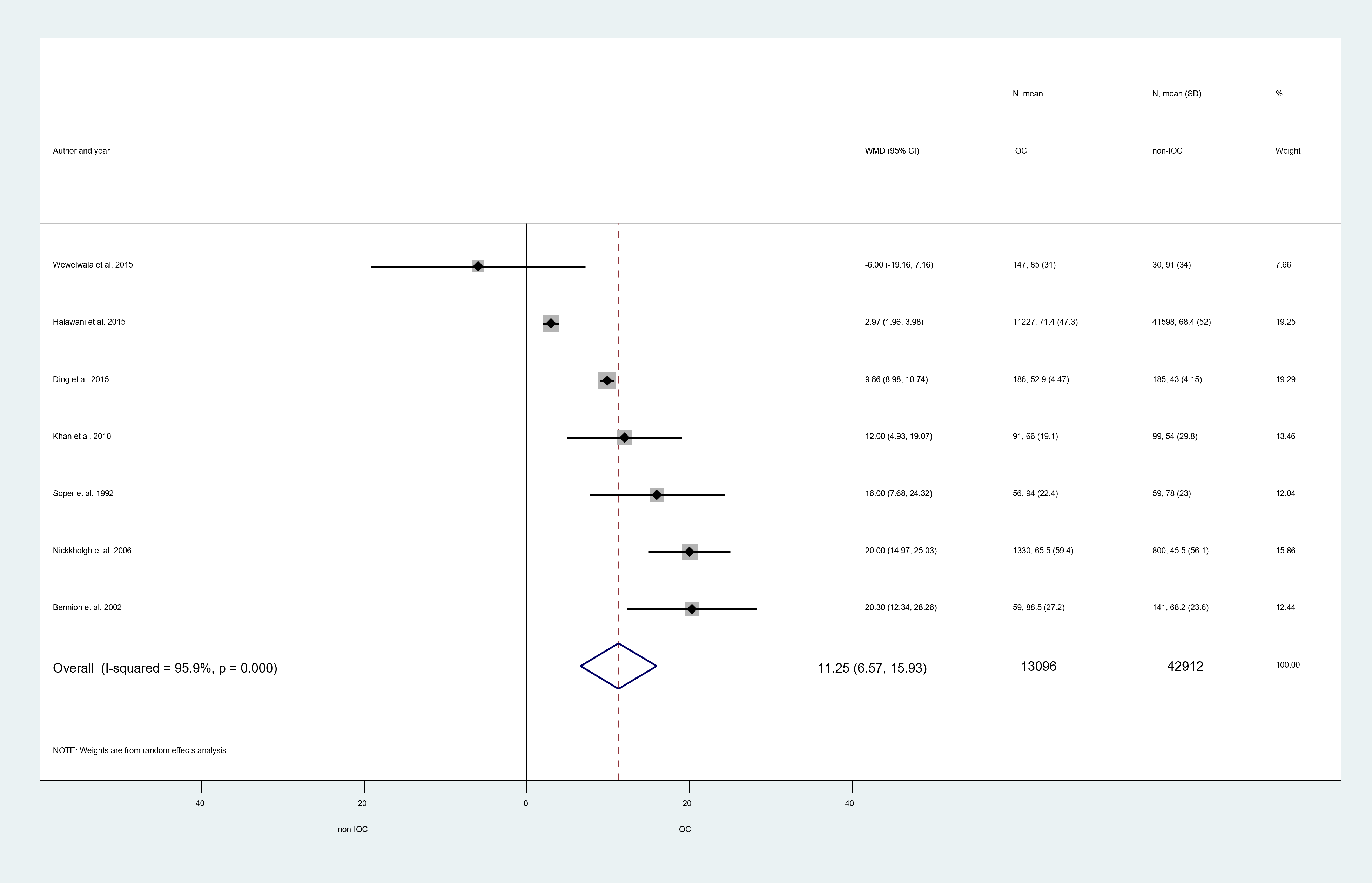

Supplement: Supplementary file 10 — Supplementary file10 (TIF 671 KB) [file 464_2022_9267_MOESM10_ESM.tif]

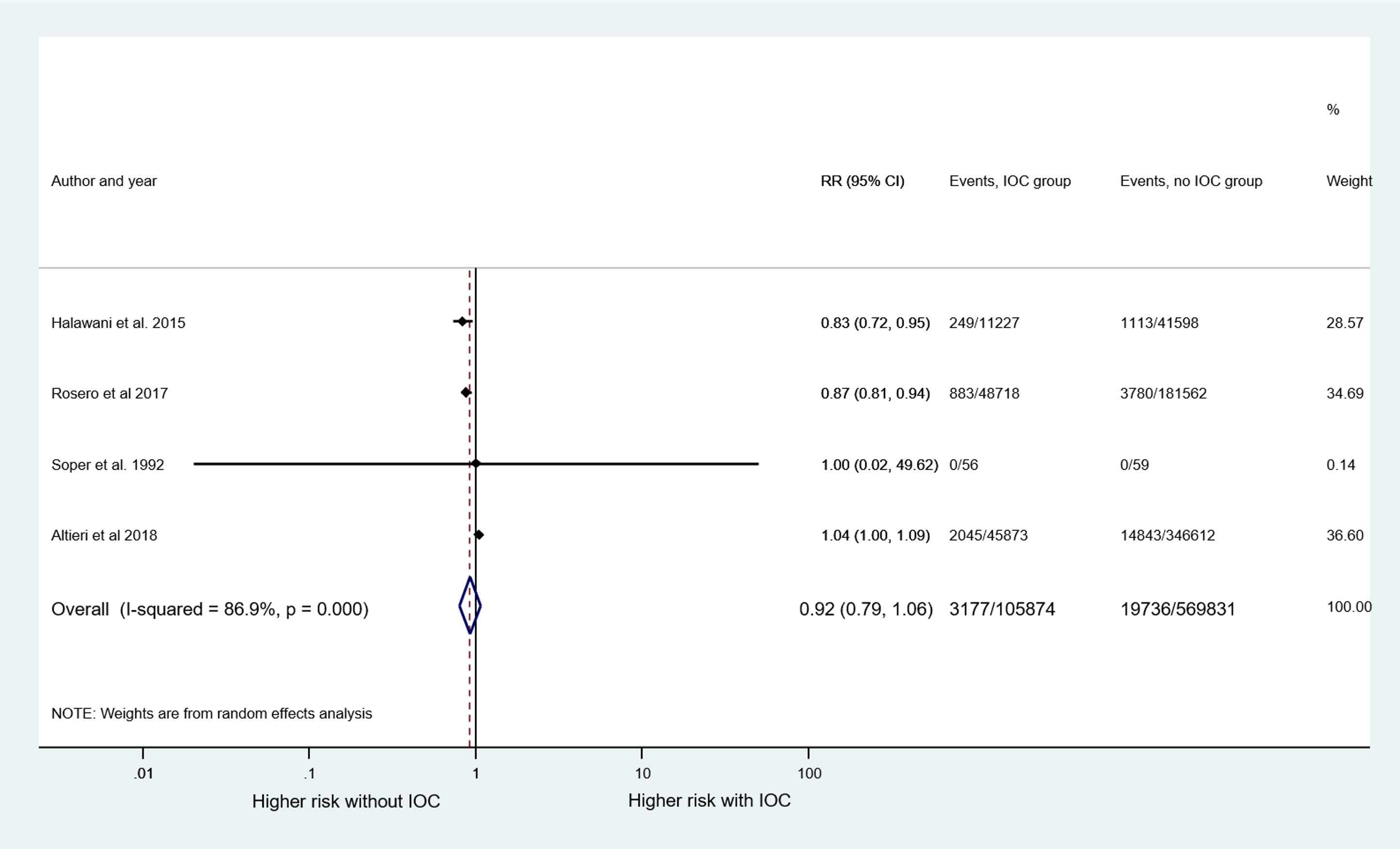

Supplement: Supplementary file 11 — Supplementary file11 (TIF 750 KB) [file 464_2022_9267_MOESM11_ESM.tif]

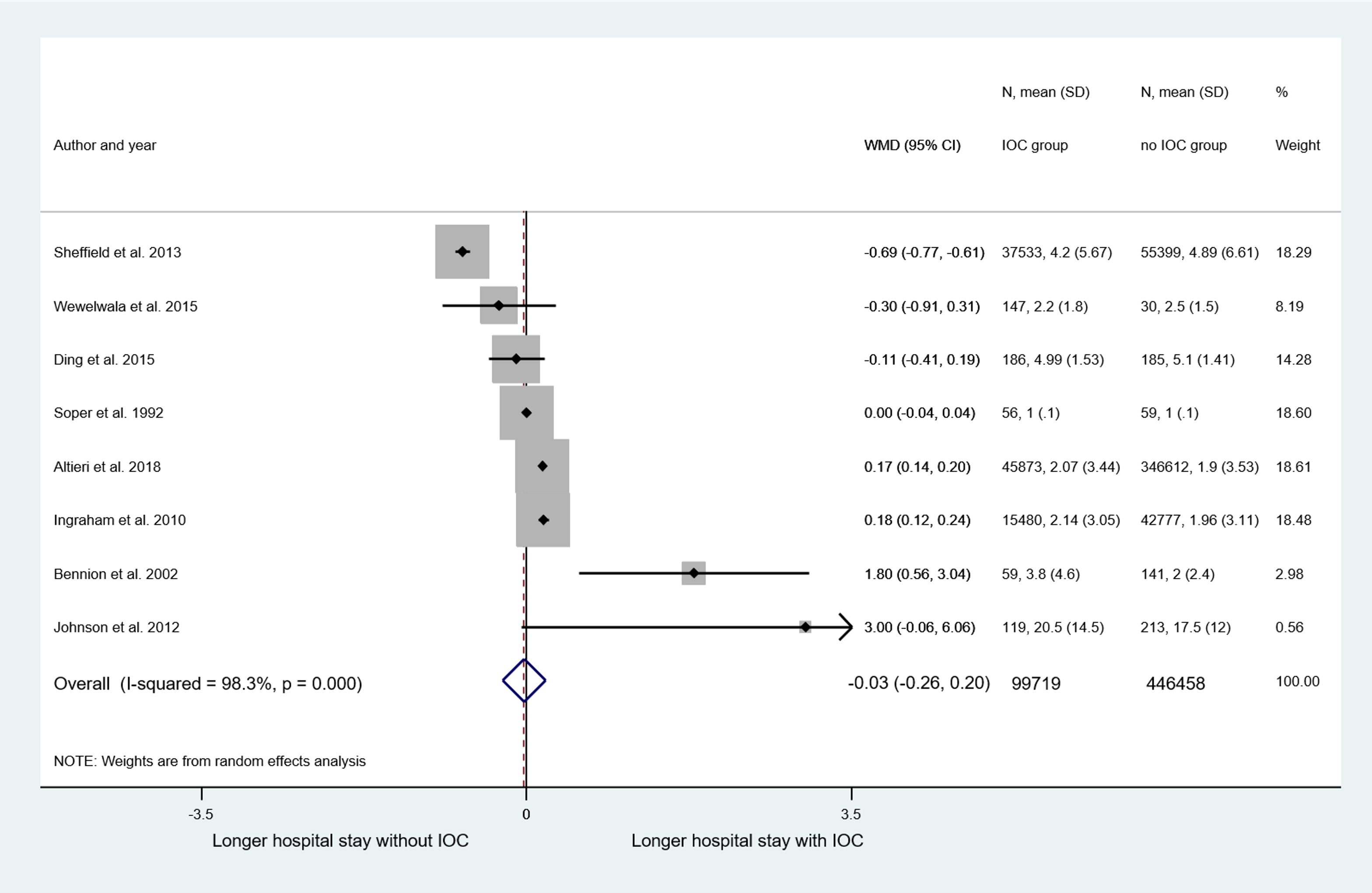

Supplement: Supplementary file 12 — Supplementary file12 (TIF 1102 KB) [file 464_2022_9267_MOESM12_ESM.tif]

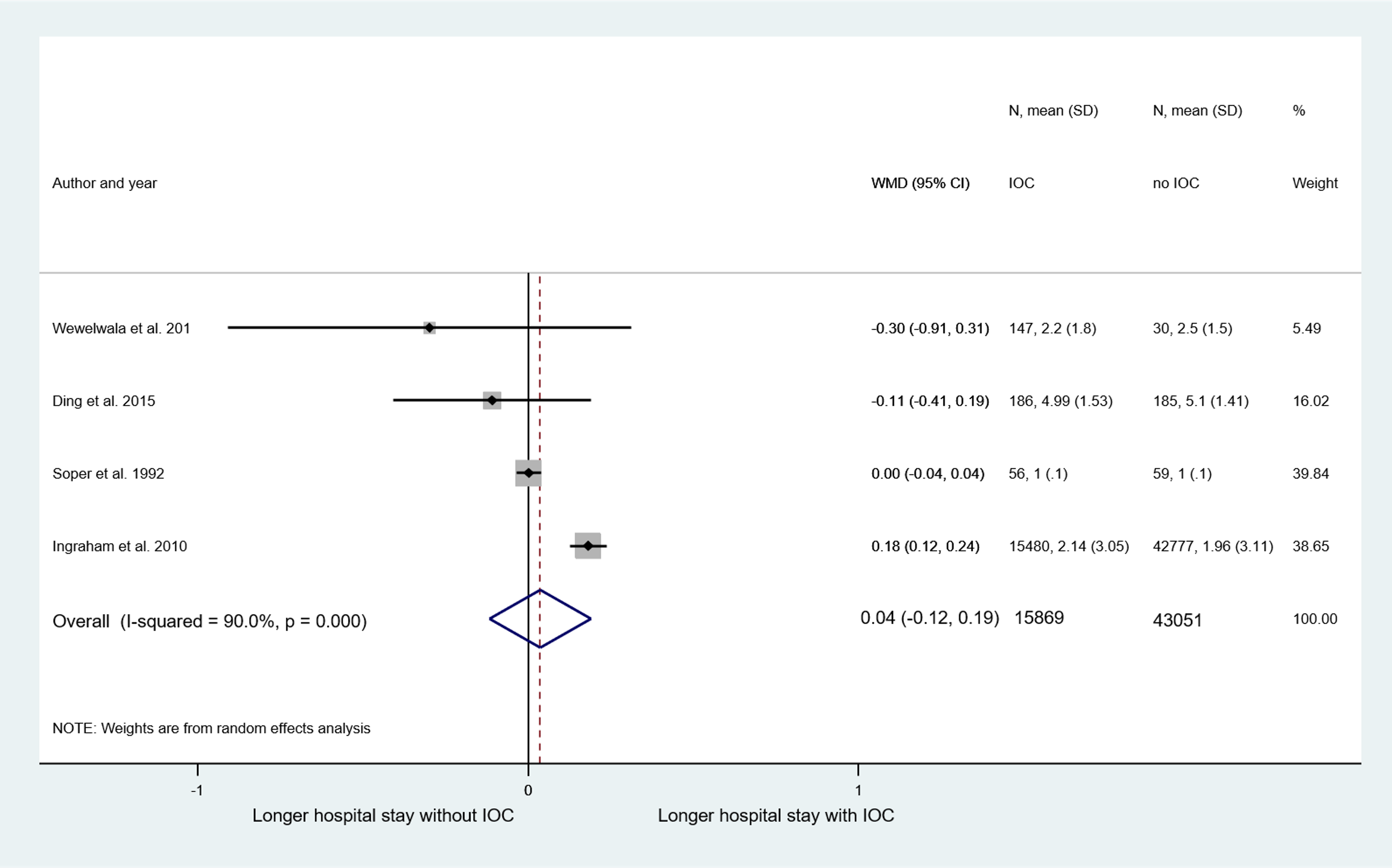

Supplement: Supplementary file 13 — Supplementary file13 (TIF 731 KB) [file 464_2022_9267_MOESM13_ESM.tif]

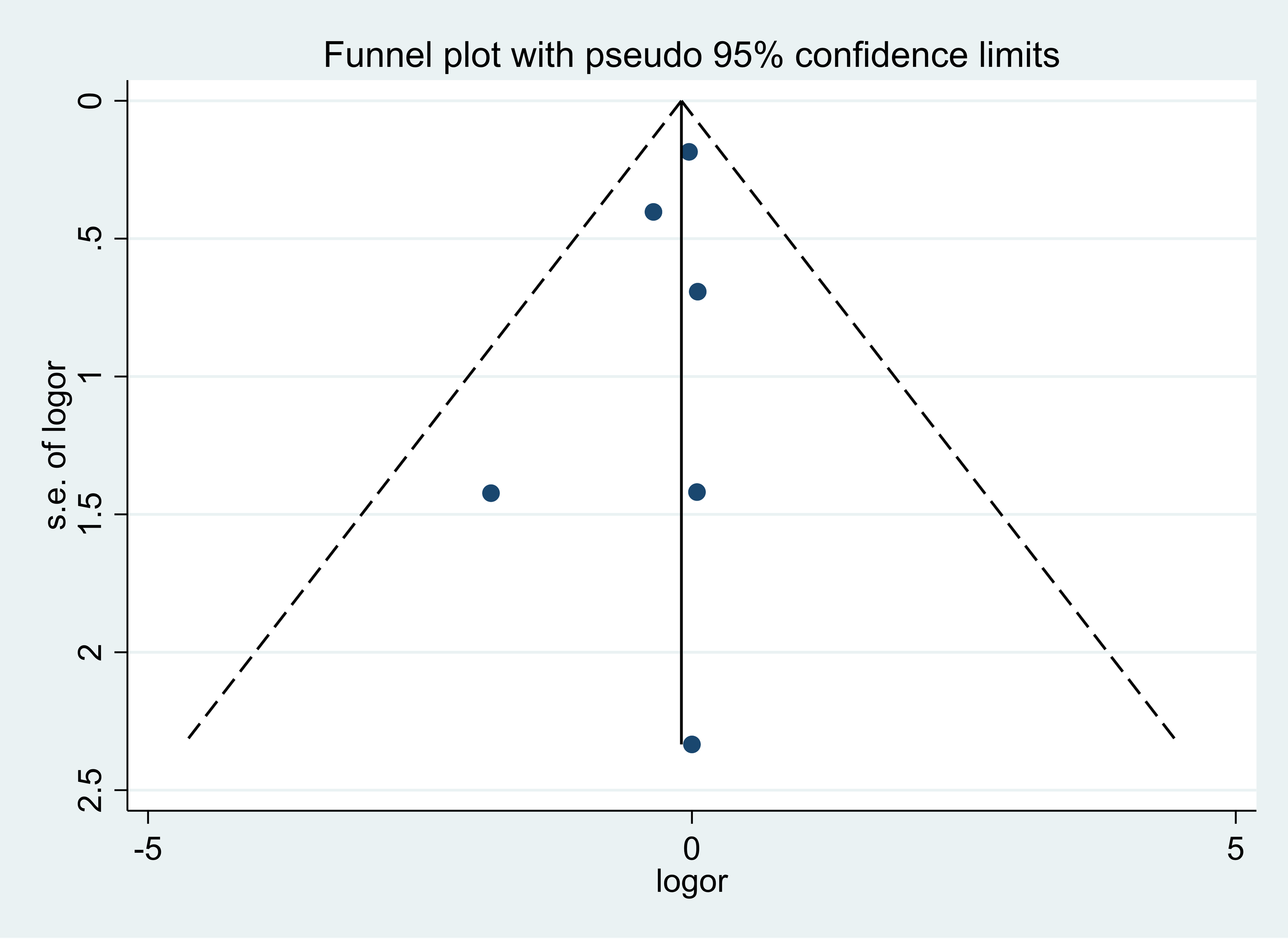

Supplement: Supplementary file 14 — Supplementary file14 (TIF 2165 KB) [file 464_2022_9267_MOESM14_ESM.tif]

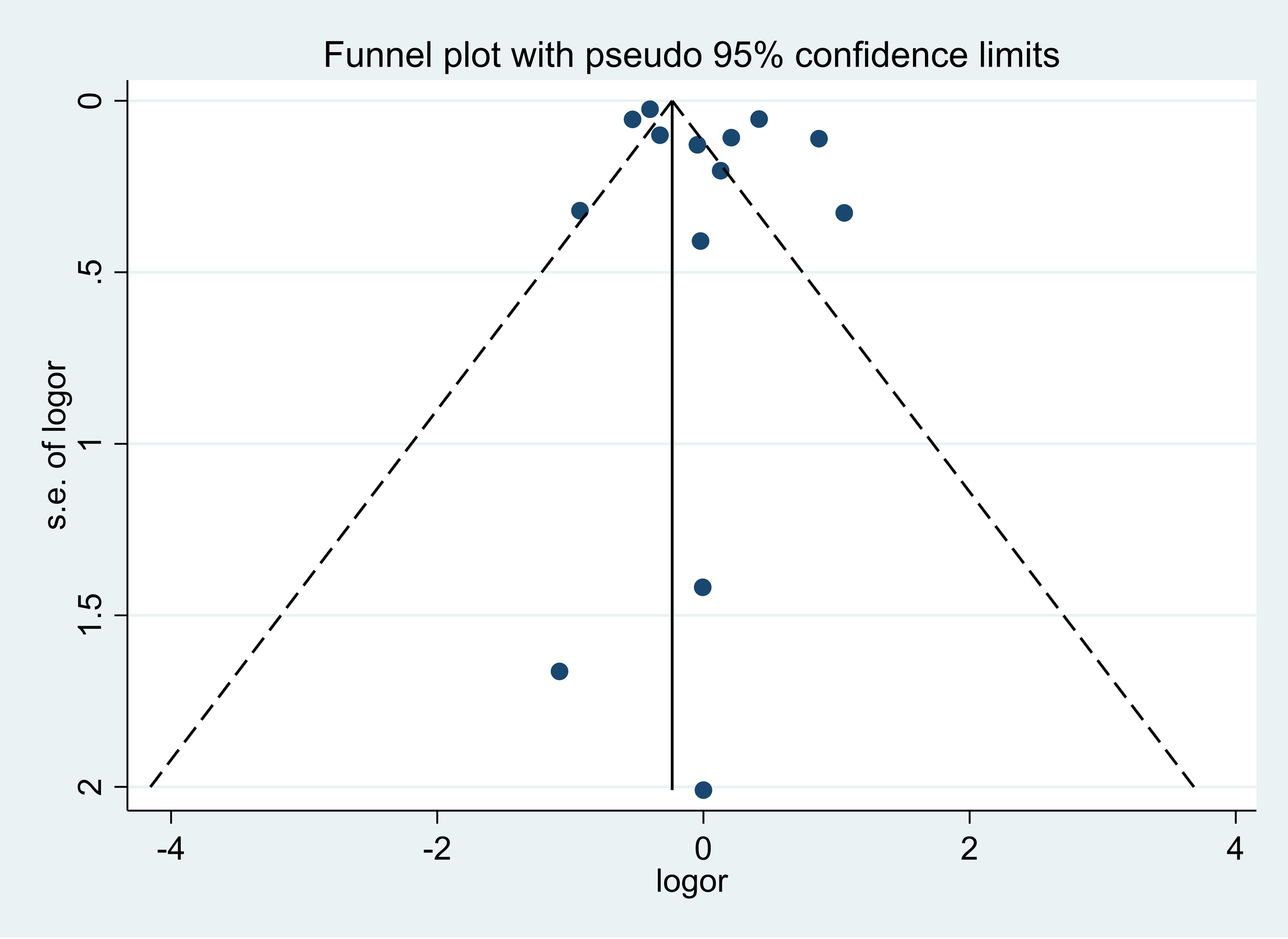

Supplement: Supplementary file 15 — Supplementary file15 (TIF 2549 KB) [file 464_2022_9267_MOESM15_ESM.tif]

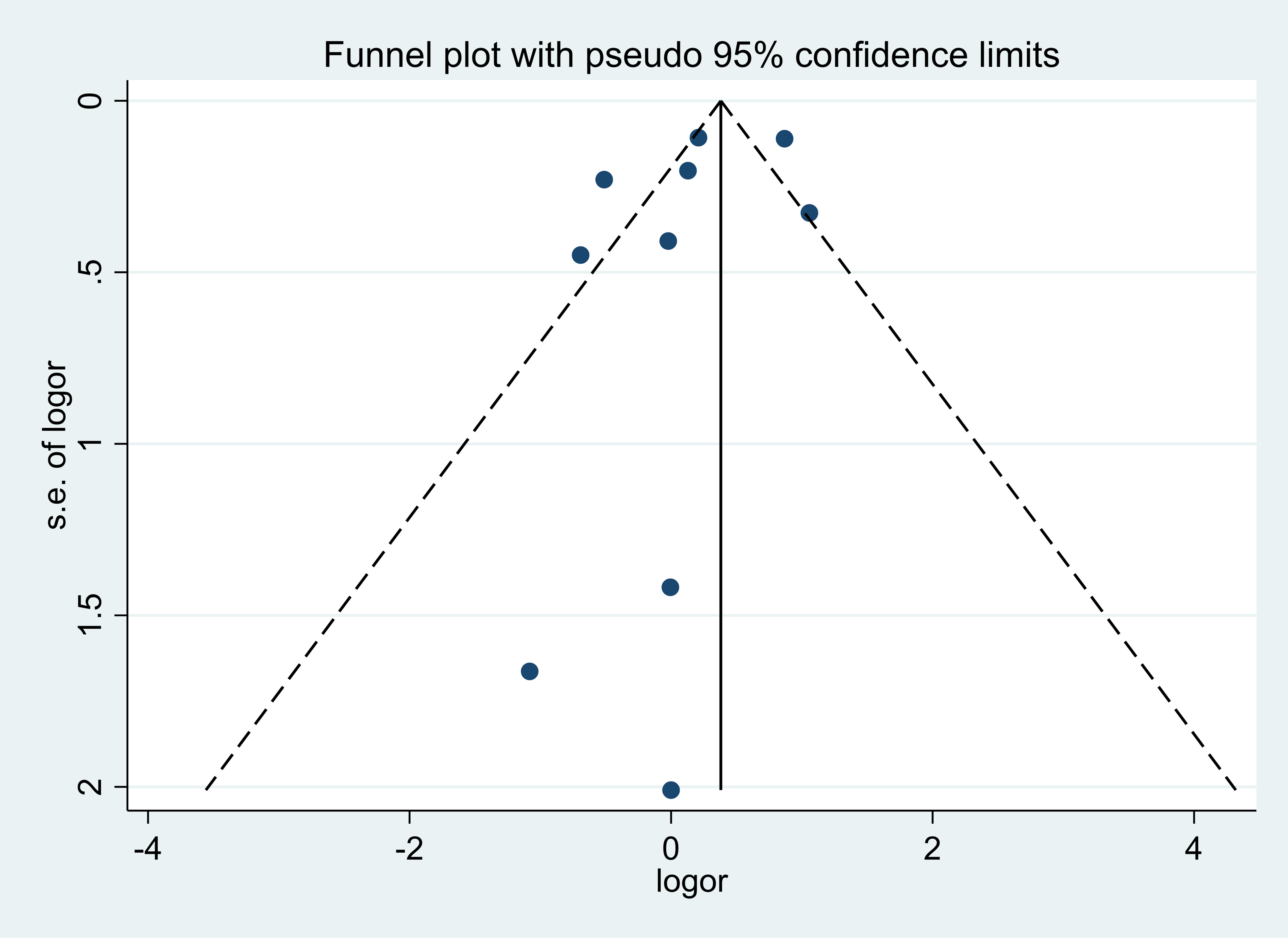

Supplement: Supplementary file 16 — Supplementary file16 (TIF 2512 KB) [file 464_2022_9267_MOESM16_ESM.tif]

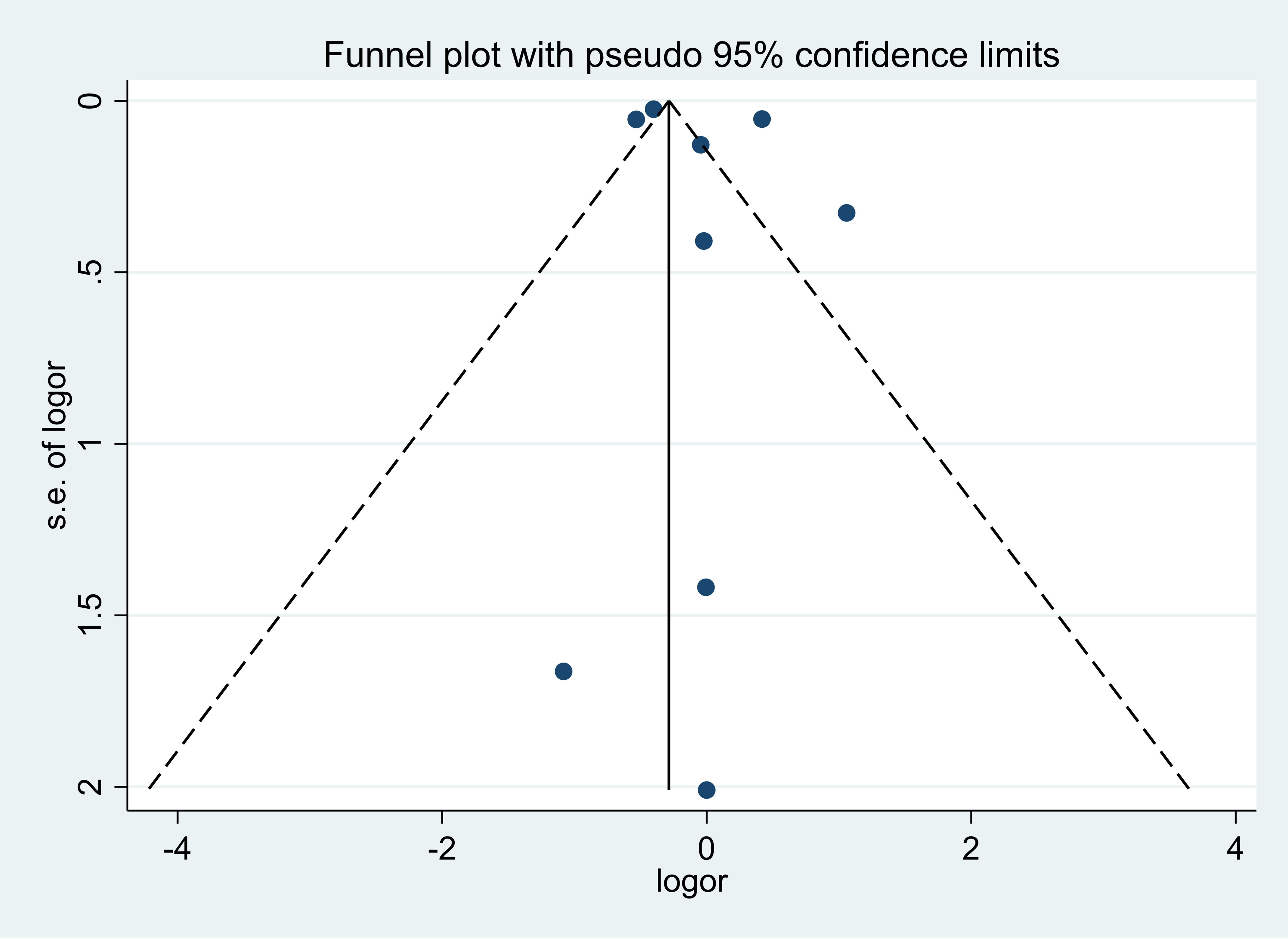

Supplement: Supplementary file 17 — Supplementary file17 (TIF 2049 KB) [file 464_2022_9267_MOESM17_ESM.tif]

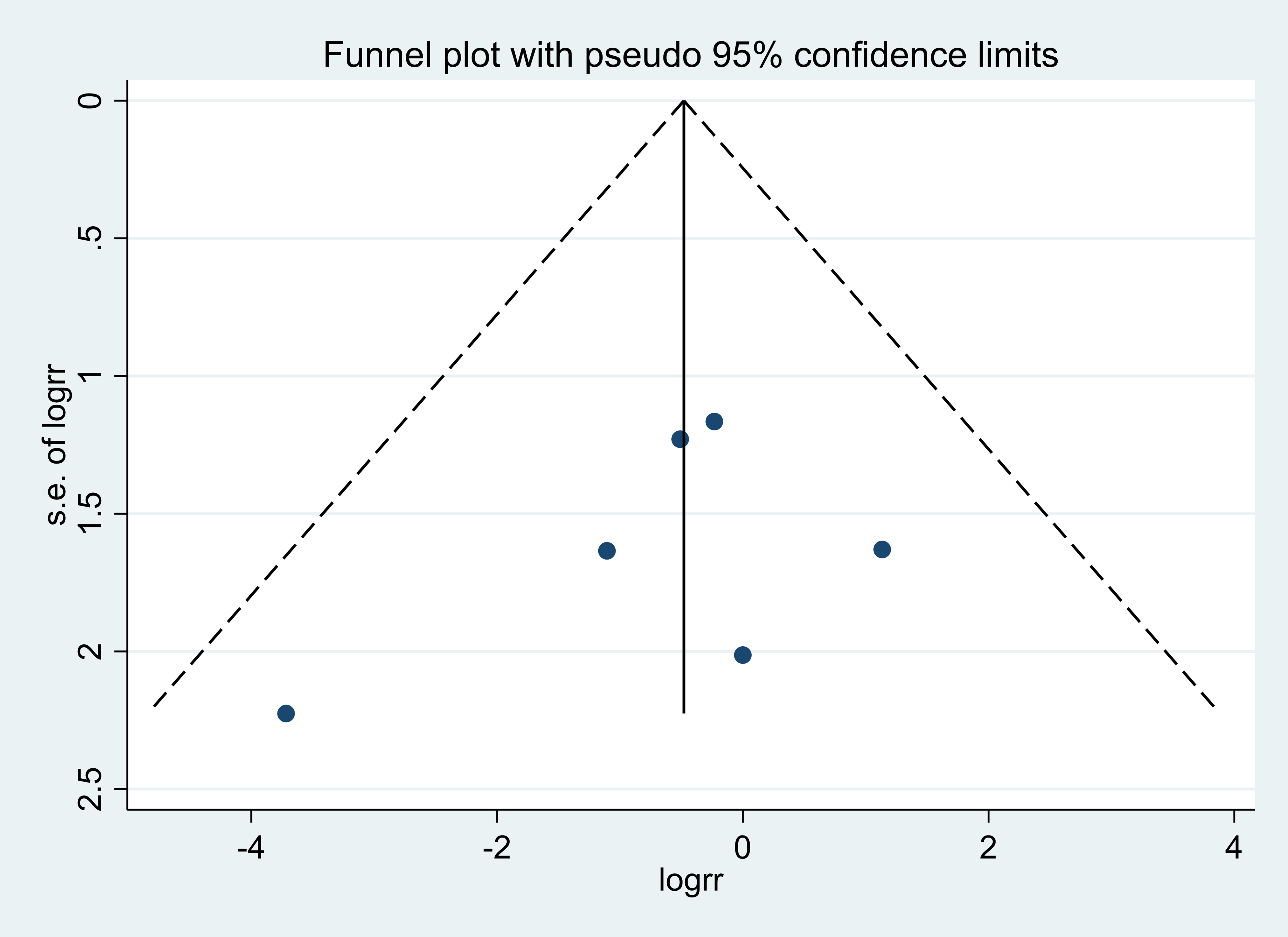

Supplement: Supplementary file 18 — Supplementary file18 (TIF 1519 KB) [file 464_2022_9267_MOESM18_ESM.tif]

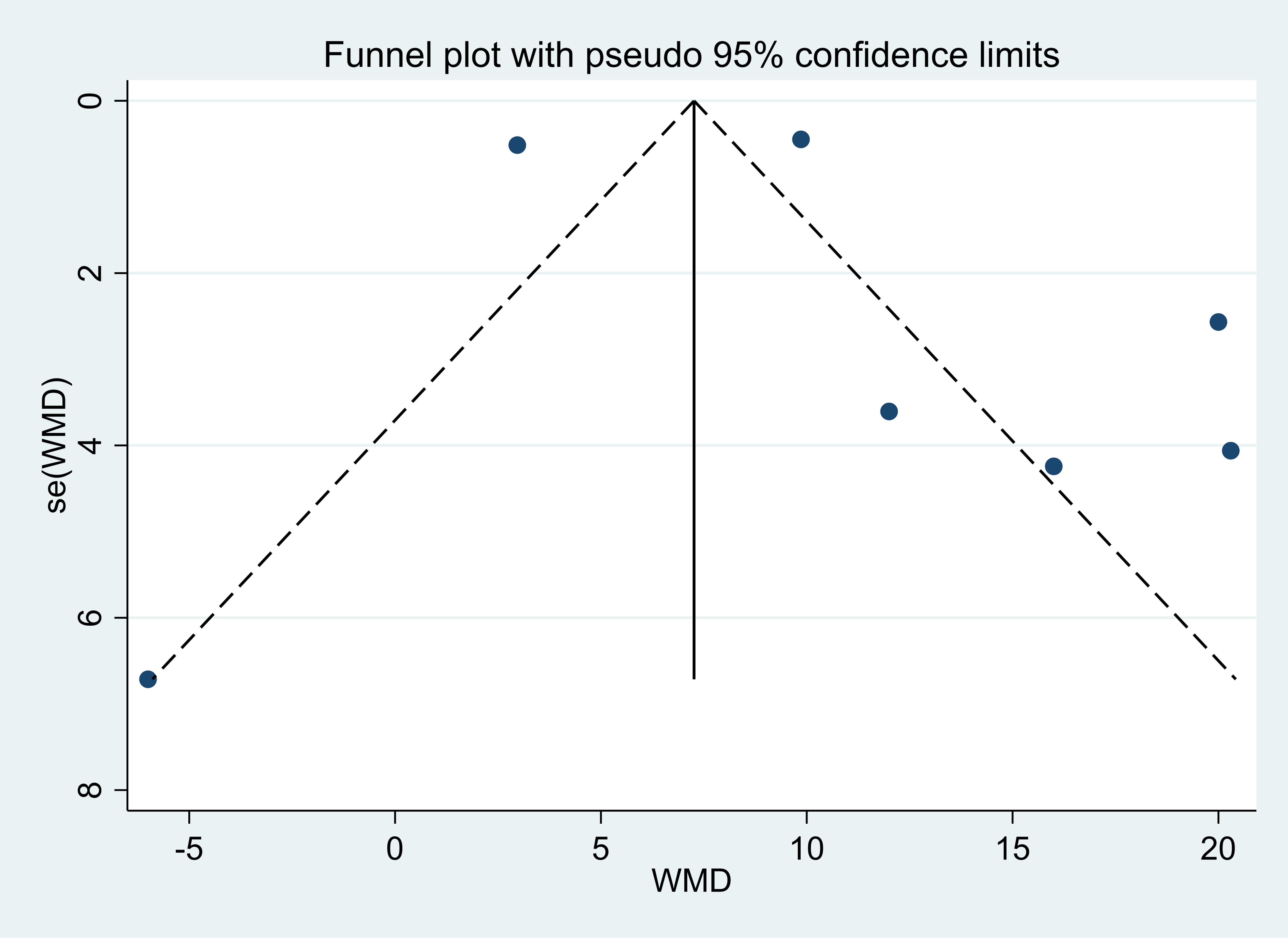

Supplement: Supplementary file 19 — Supplementary file19 (TIF 2199 KB) [file 464_2022_9267_MOESM19_ESM.tif]

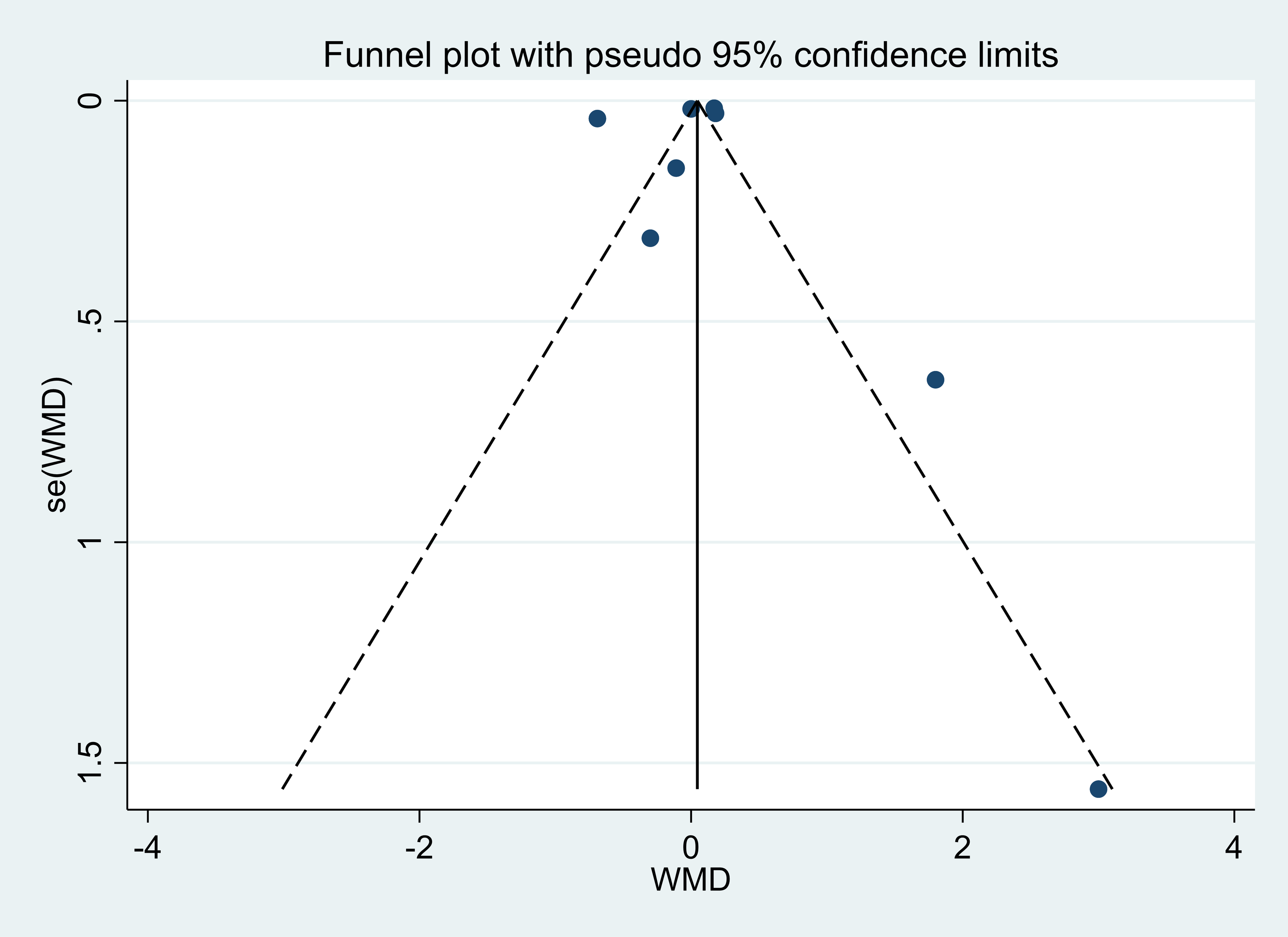

Supplement: Supplementary file 20 — Supplementary file20 (TIF 1543 KB) [file 464_2022_9267_MOESM20_ESM.tif]

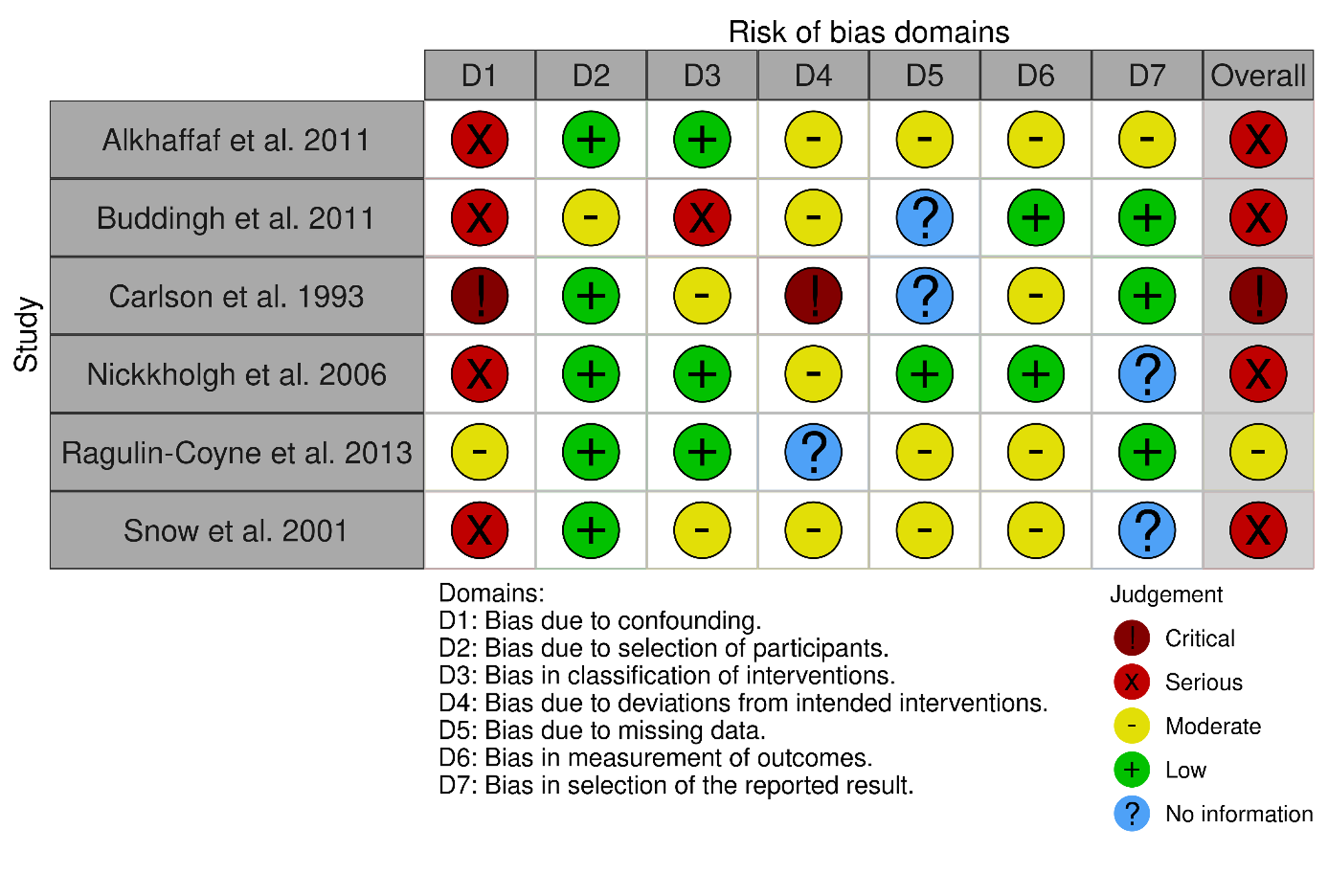

Supplement: Supplementary file 21 — Supplementary file21 (TIF 1129 KB) [file 464_2022_9267_MOESM21_ESM.tif]

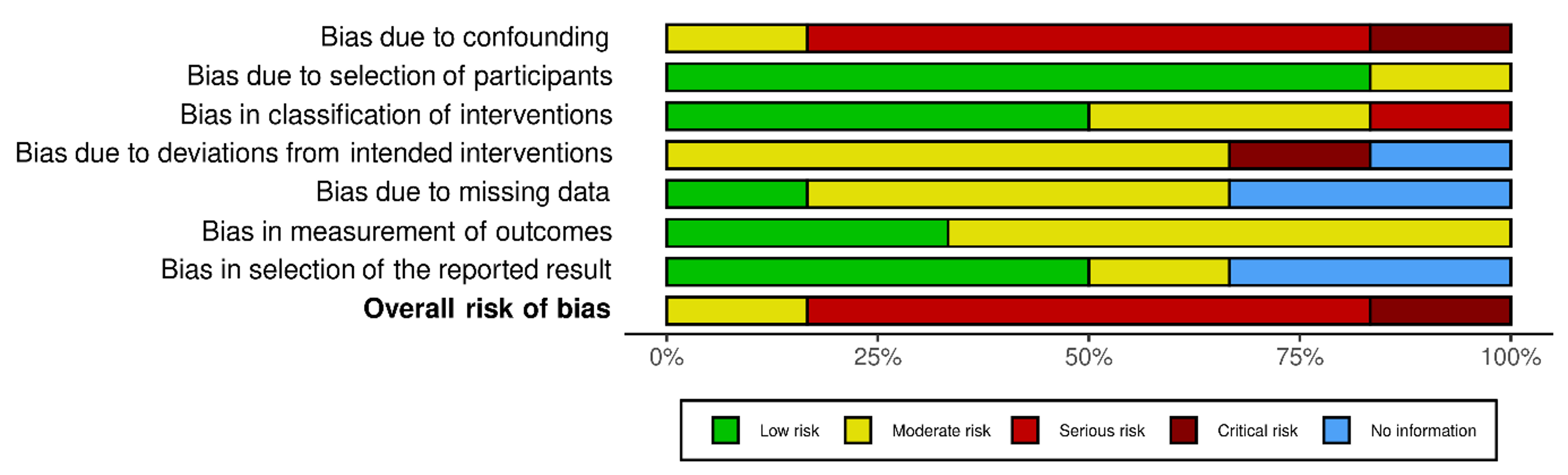

Supplement: Supplementary file 22 — Supplementary file22 (TIF 503 KB) [file 464_2022_9267_MOESM22_ESM.tif]

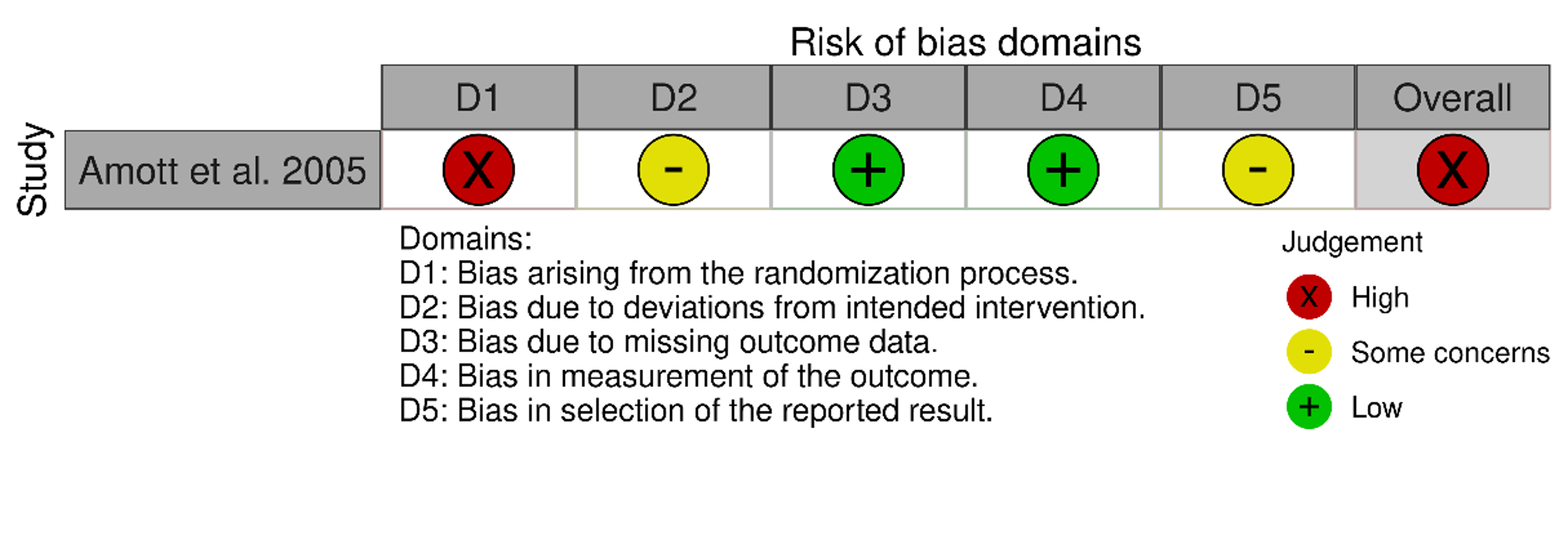

Supplement: Supplementary file 23 — Supplementary file23 (TIF 724 KB) [file 464_2022_9267_MOESM23_ESM.tif]

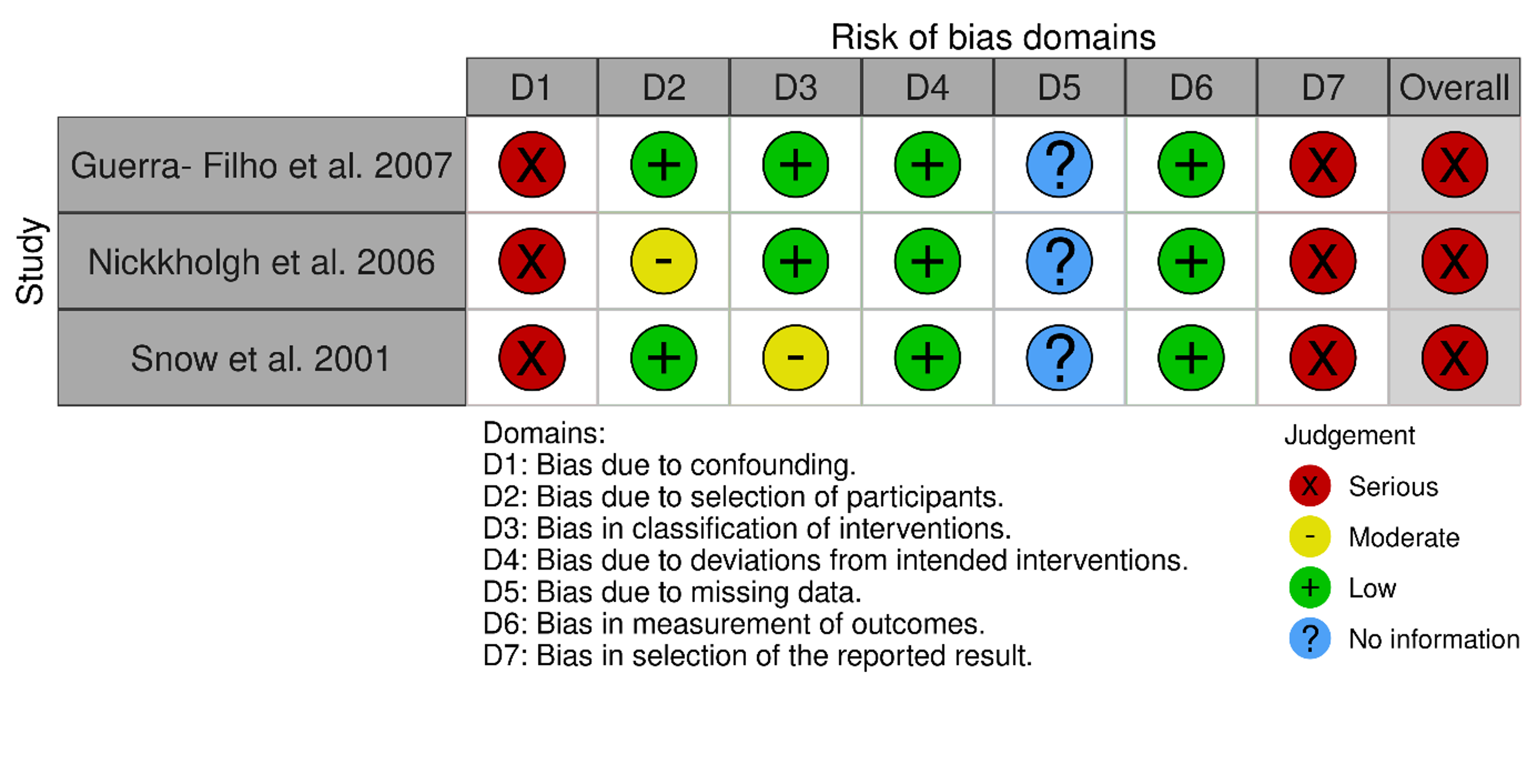

Supplement: Supplementary file 24 — Supplementary file24 (TIF 478 KB) [file 464_2022_9267_MOESM24_ESM.tif]

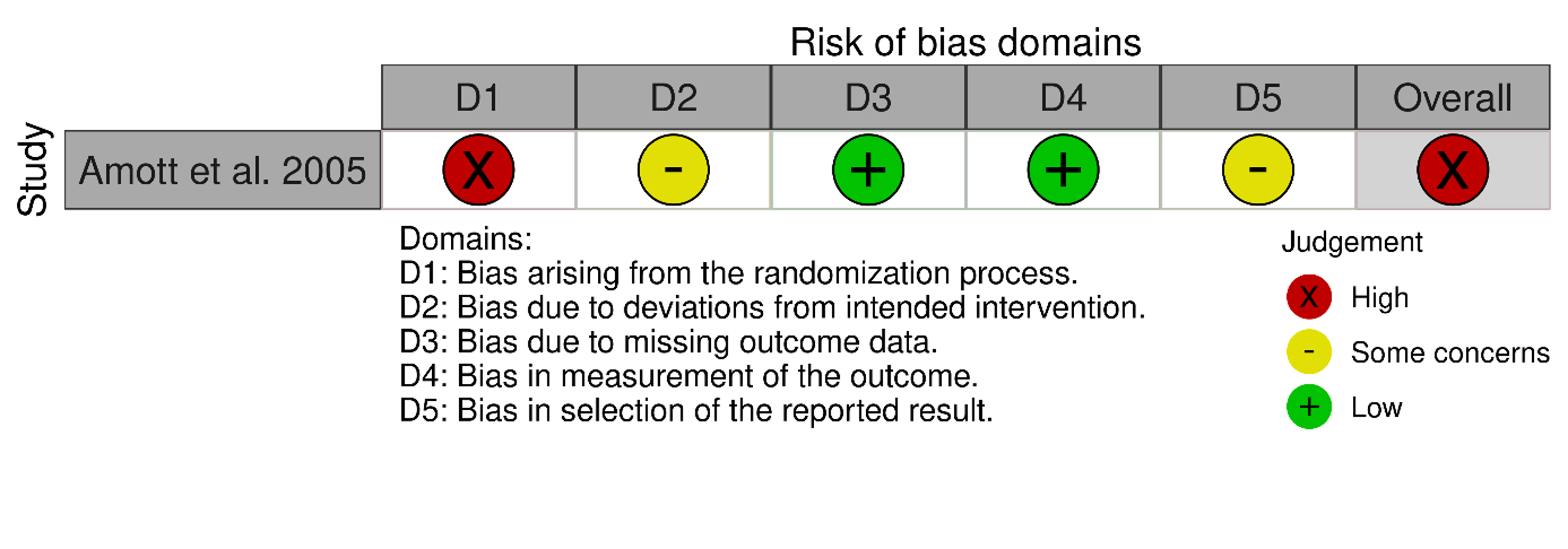

Supplement: Supplementary file 25 — Supplementary file25 (TIF 700 KB) [file 464_2022_9267_MOESM25_ESM.tif]

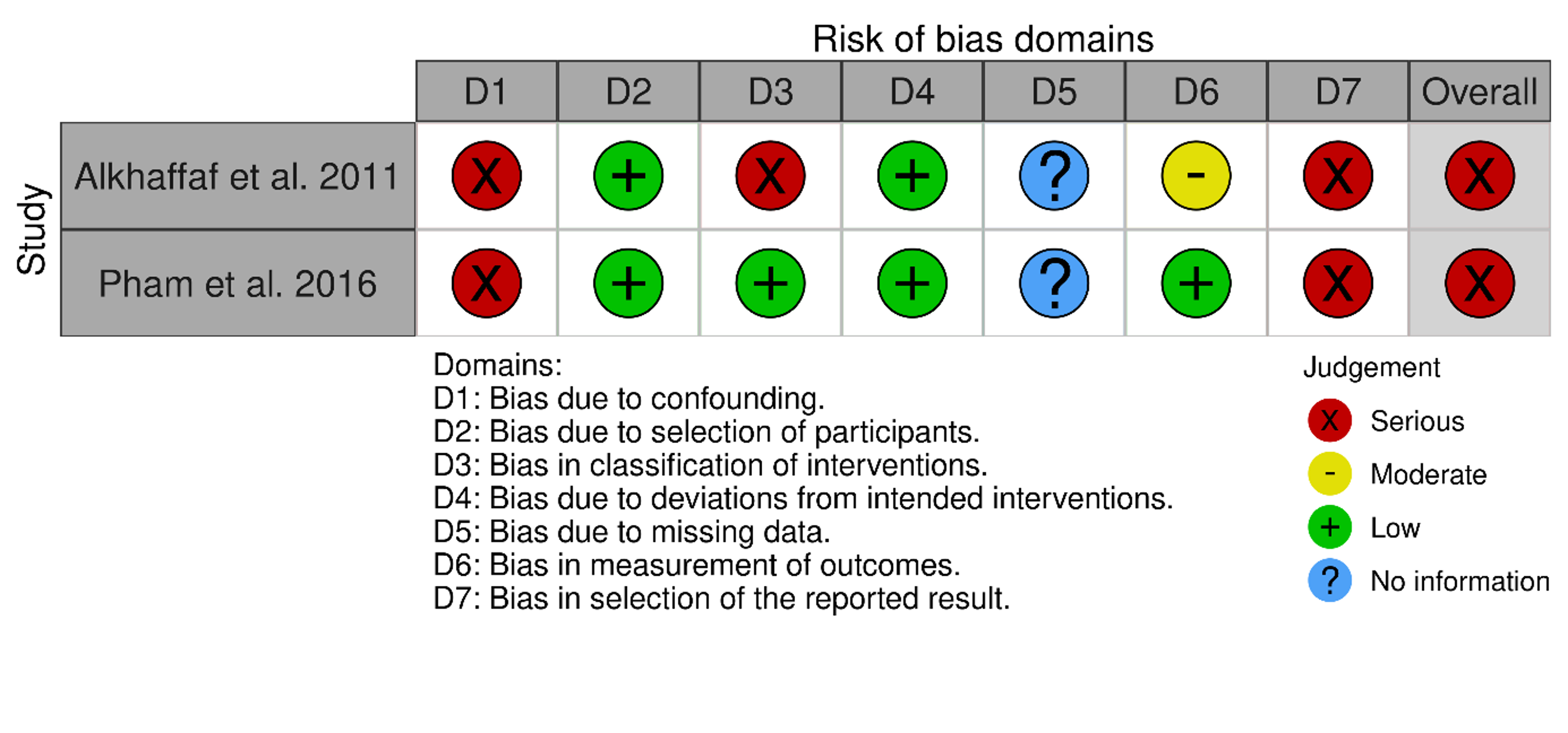

Supplement: Supplementary file 26 — Supplementary file26 (TIF 1075 KB) [file 464_2022_9267_MOESM26_ESM.tif]

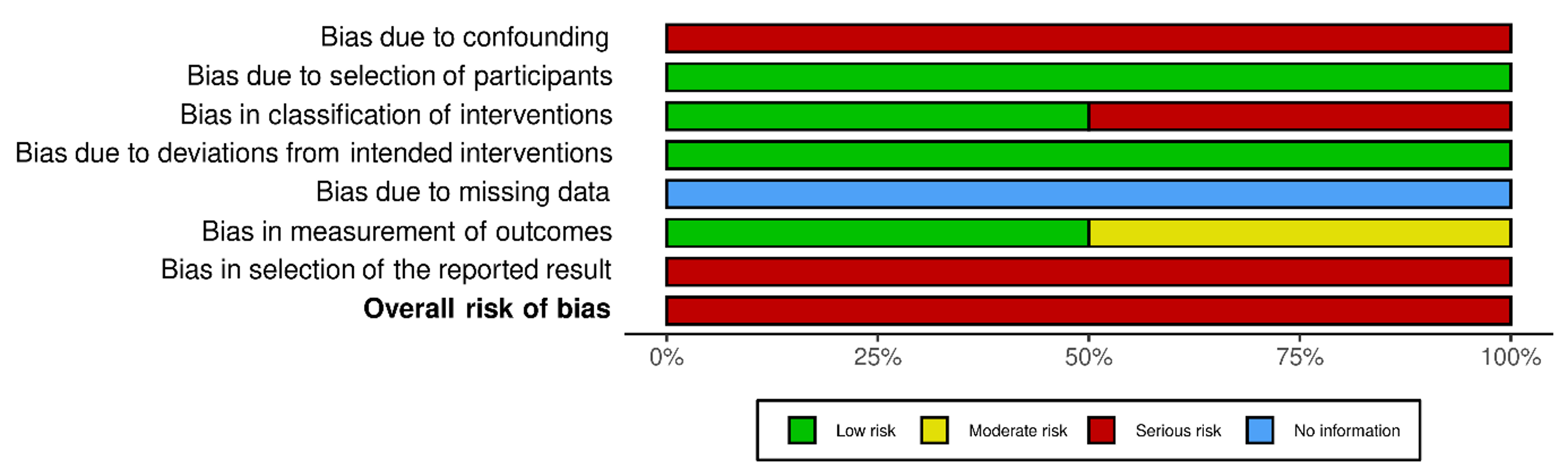

Supplement: Supplementary file 27 — Supplementary file27 (TIF 477 KB) [file 464_2022_9267_MOESM27_ESM.tif]

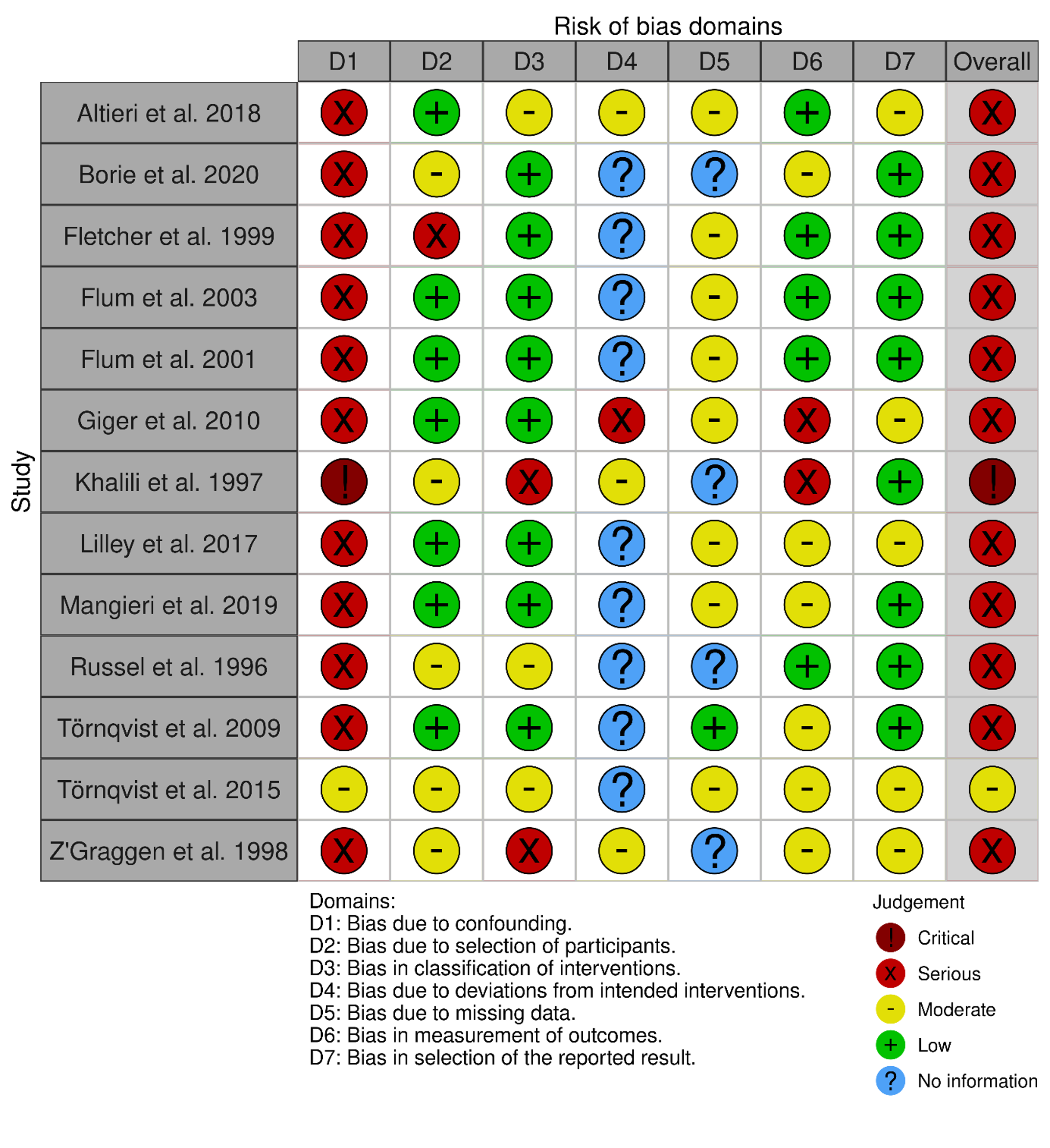

Supplement: Supplementary file 28 — Supplementary file28 (TIF 2700 KB) [file 464_2022_9267_MOESM28_ESM.tif]

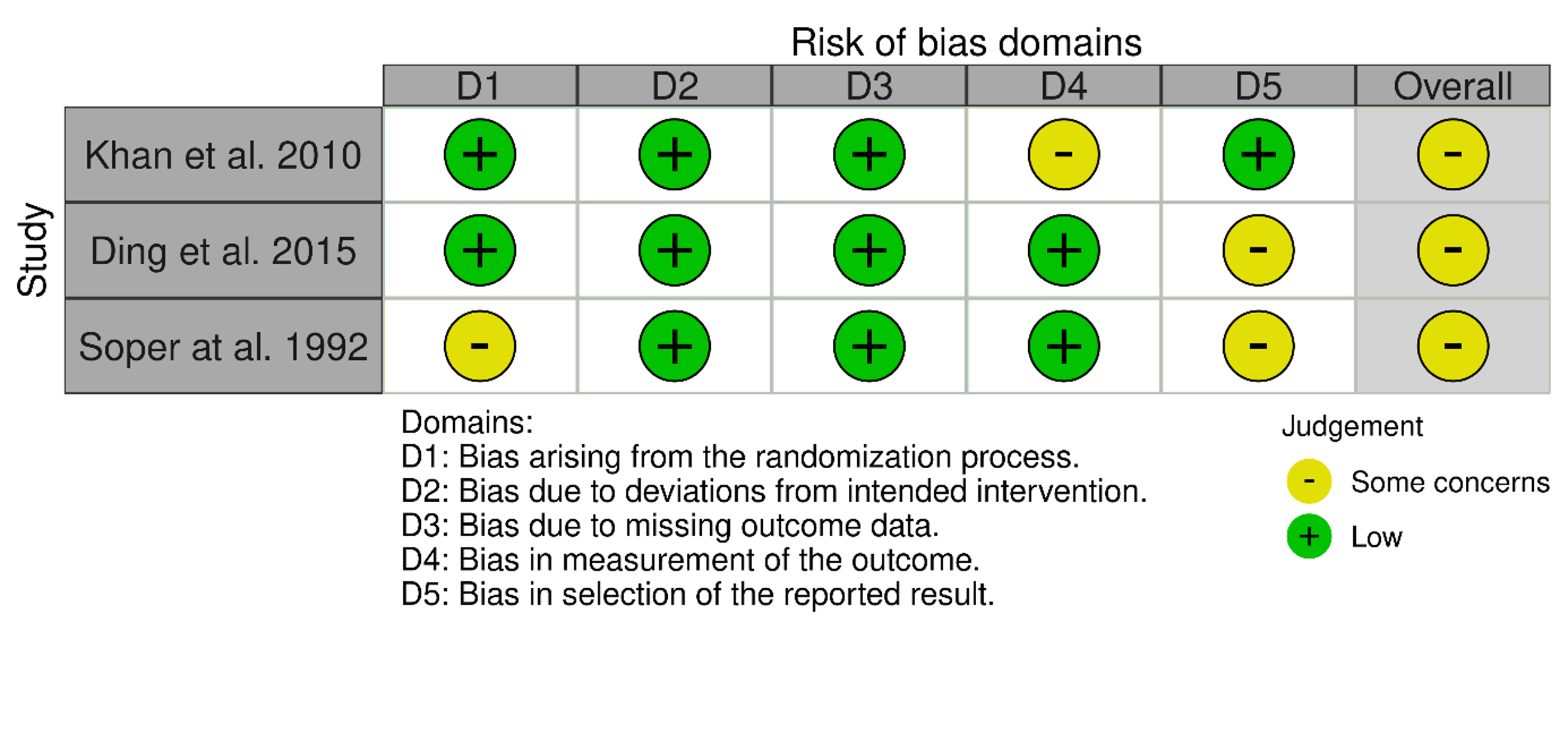

Supplement: Supplementary file 30 — Supplementary file30 (TIF 1020 KB) [file 464_2022_9267_MOESM30_ESM.tif]

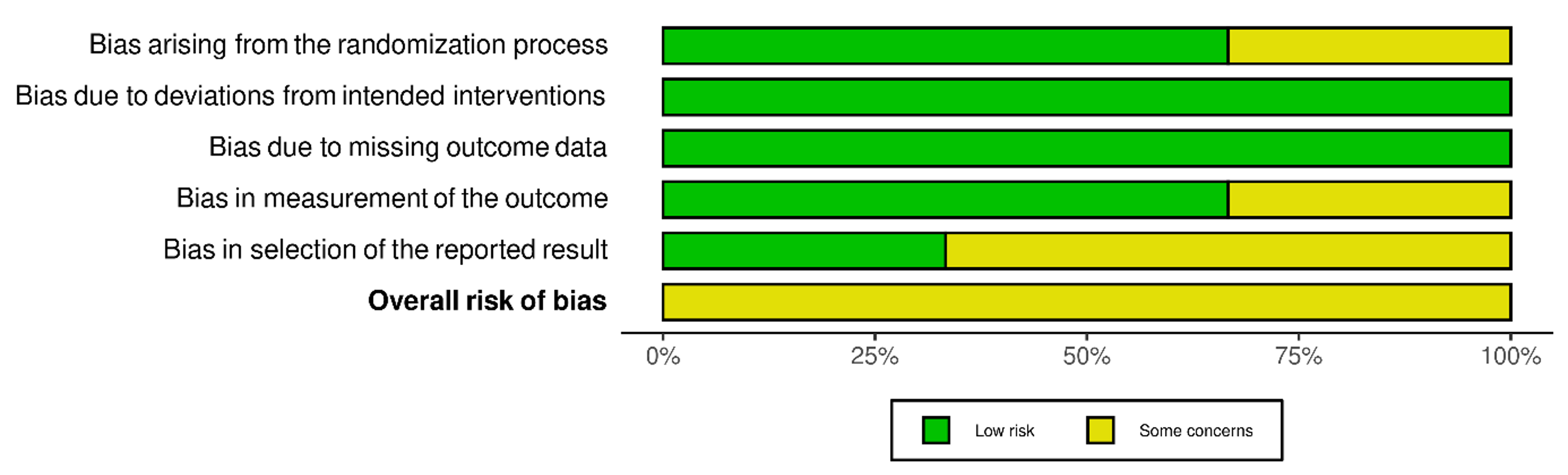

Supplement: Supplementary file 31 — Supplementary file31 (TIF 419 KB) [file 464_2022_9267_MOESM31_ESM.tif]

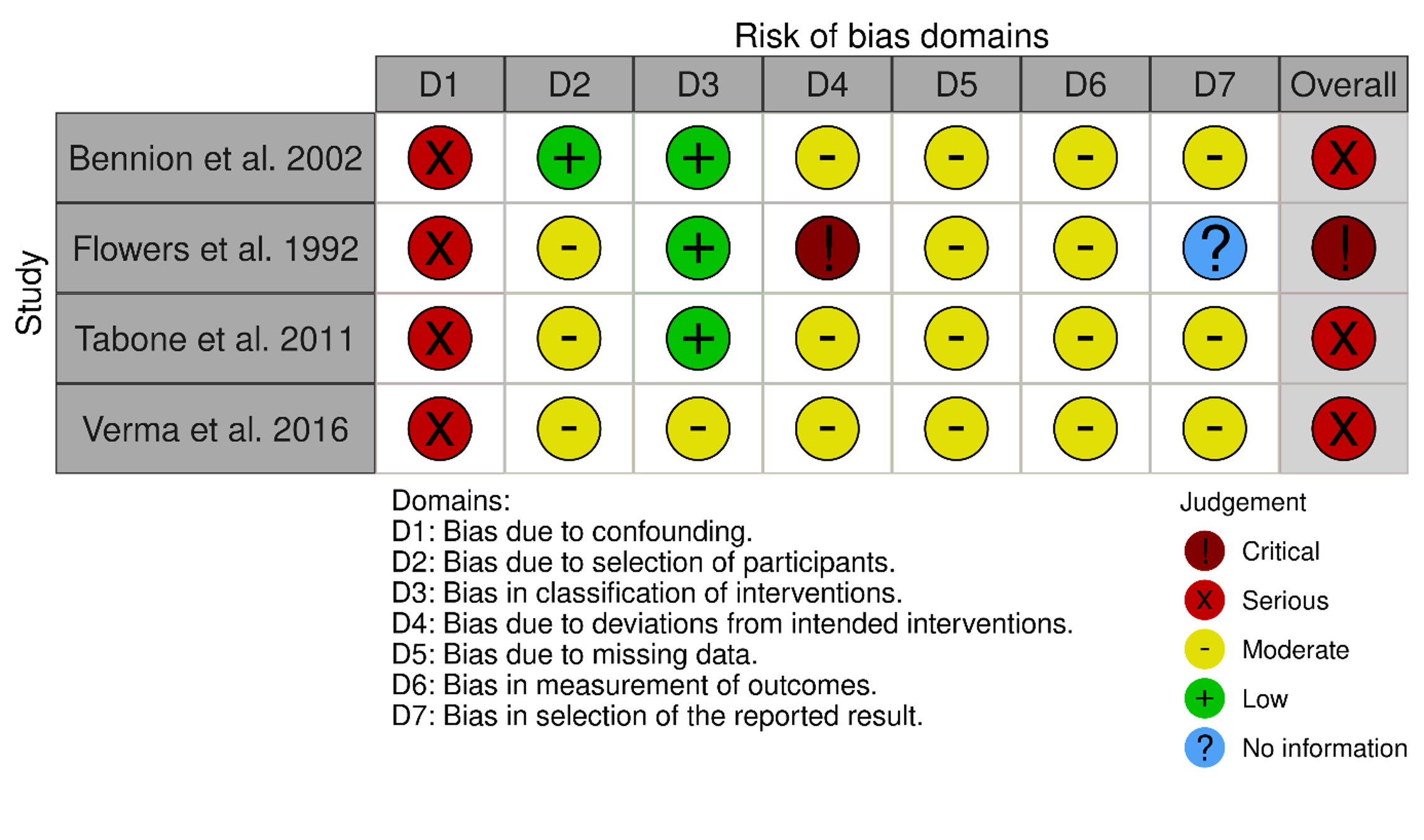

Supplement: Supplementary file 32 — Supplementary file32 (TIF 1147 KB) [file 464_2022_9267_MOESM32_ESM.tif]

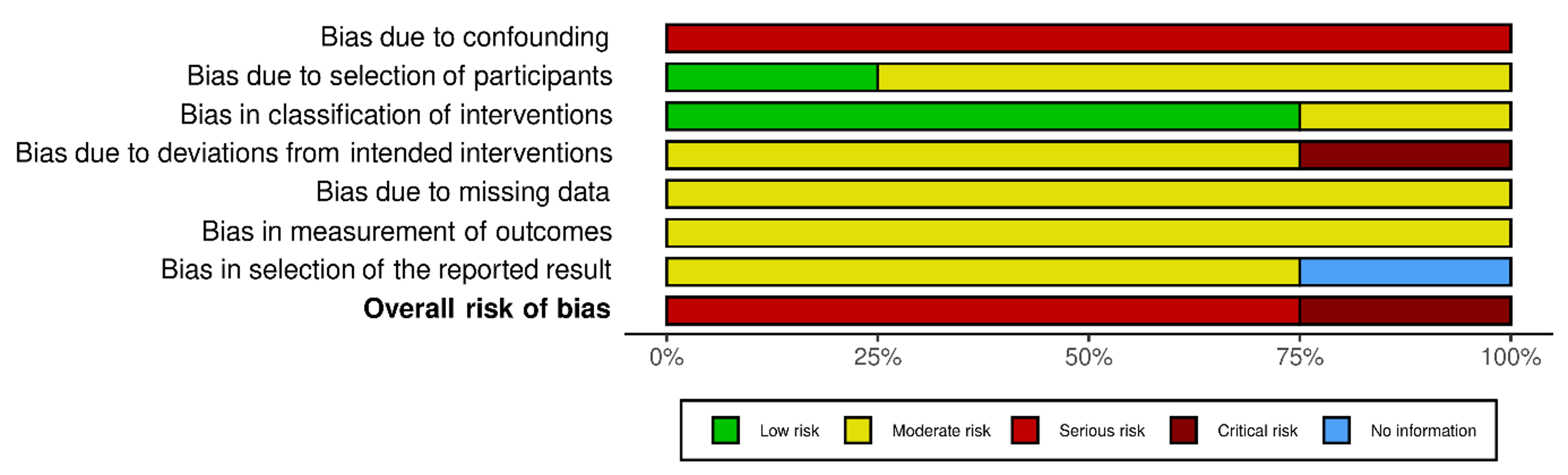

Supplement: Supplementary file 33 — Supplementary file33 (TIF 490 KB) [file 464_2022_9267_MOESM33_ESM.tif]

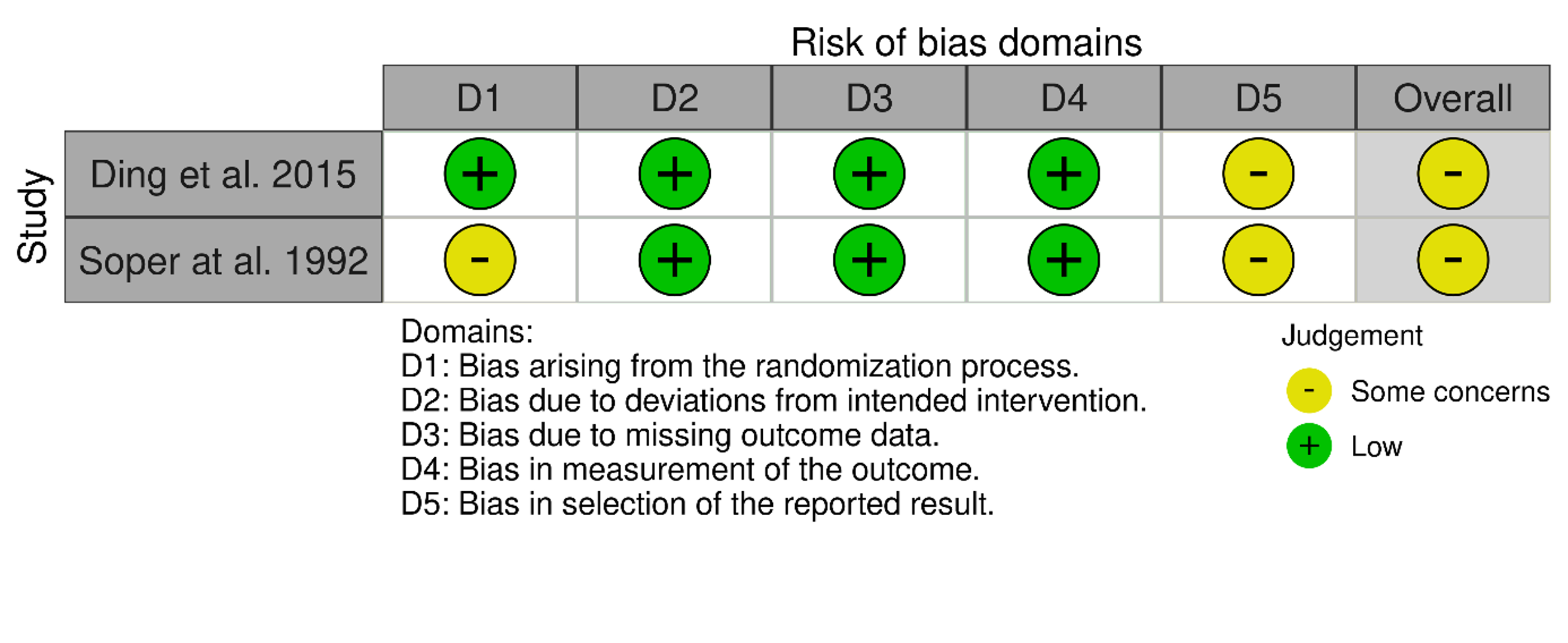

Supplement: Supplementary file 34 — Supplementary file34 (TIF 856 KB) [file 464_2022_9267_MOESM34_ESM.tif]

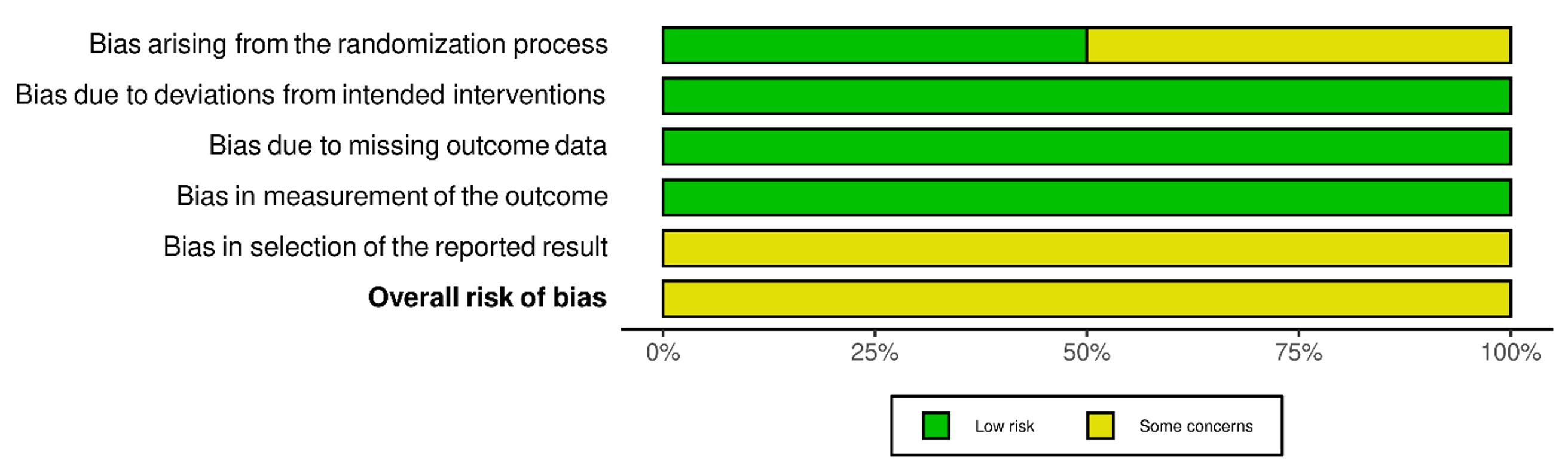

Supplement: Supplementary file 35 — Supplementary file35 (TIF 411 KB) [file 464_2022_9267_MOESM35_ESM.tif]

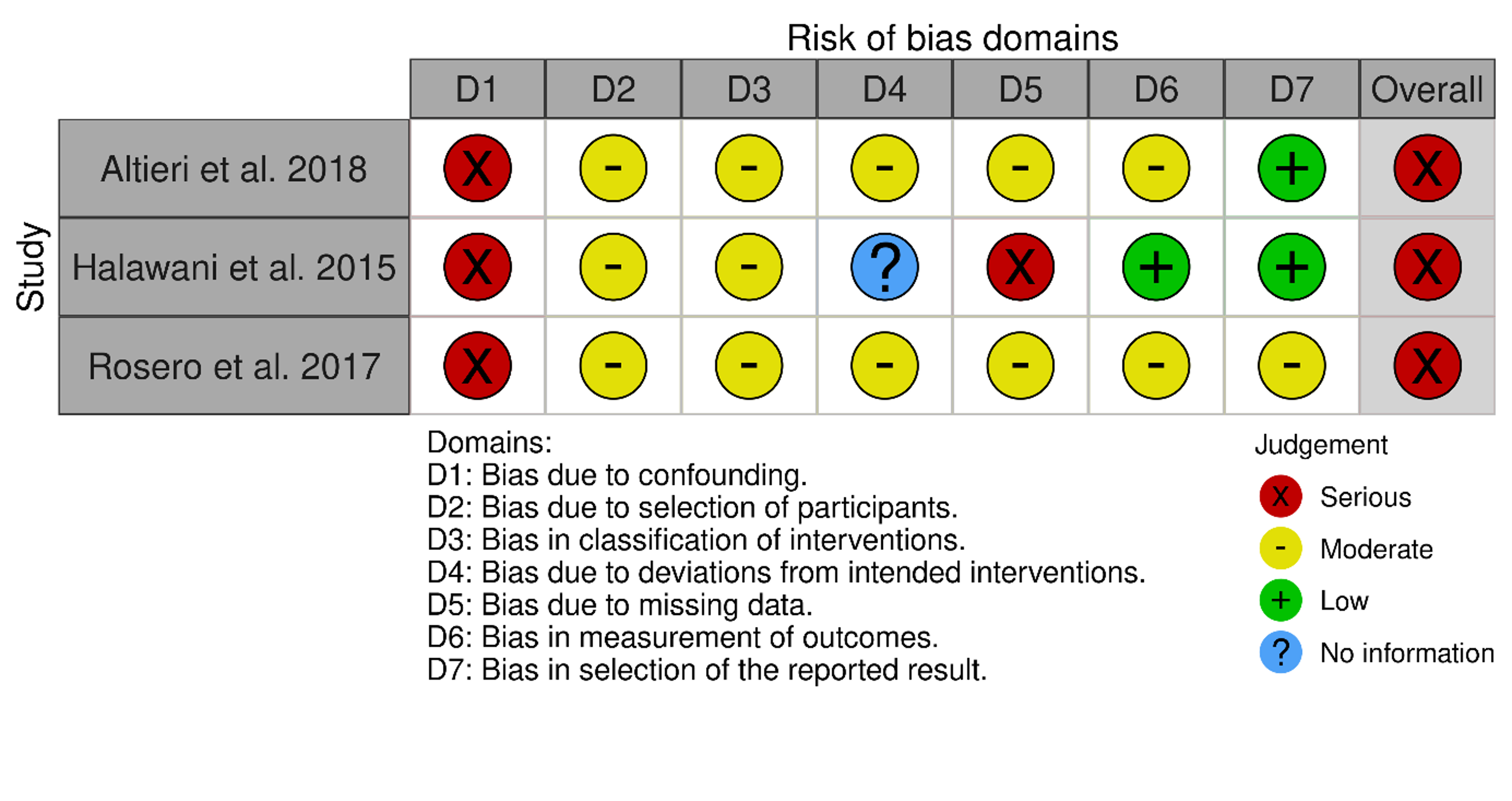

Supplement: Supplementary file 36 — Supplementary file36 (TIF 1180 KB) [file 464_2022_9267_MOESM36_ESM.tif]

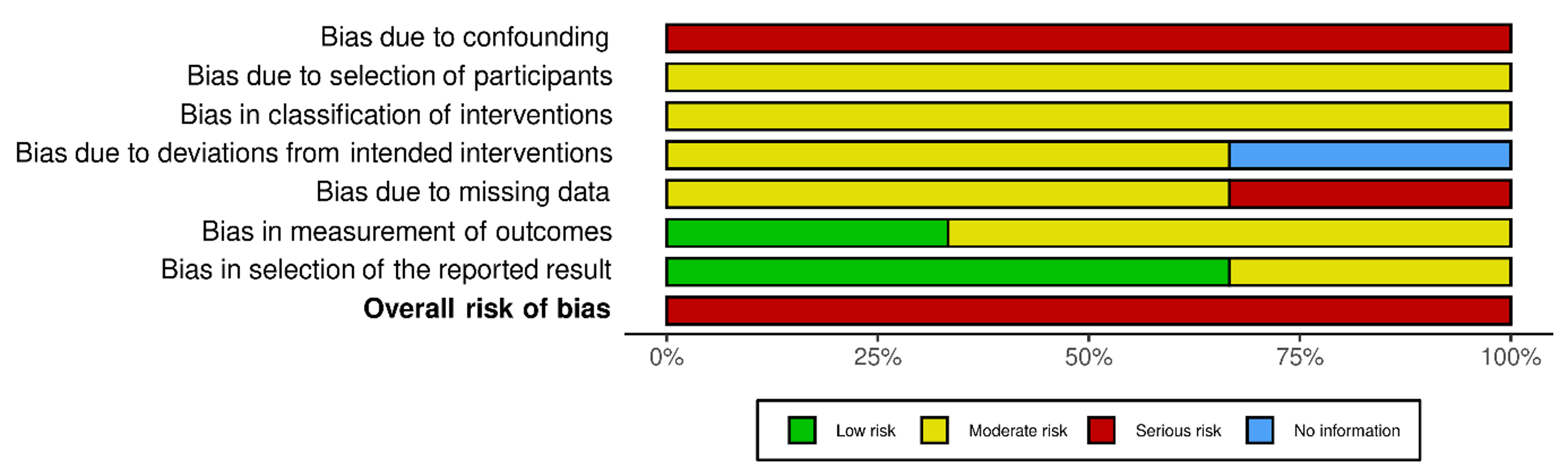

Supplement: Supplementary file 37 — Supplementary file37 (TIF 482 KB) [file 464_2022_9267_MOESM37_ESM.tif]

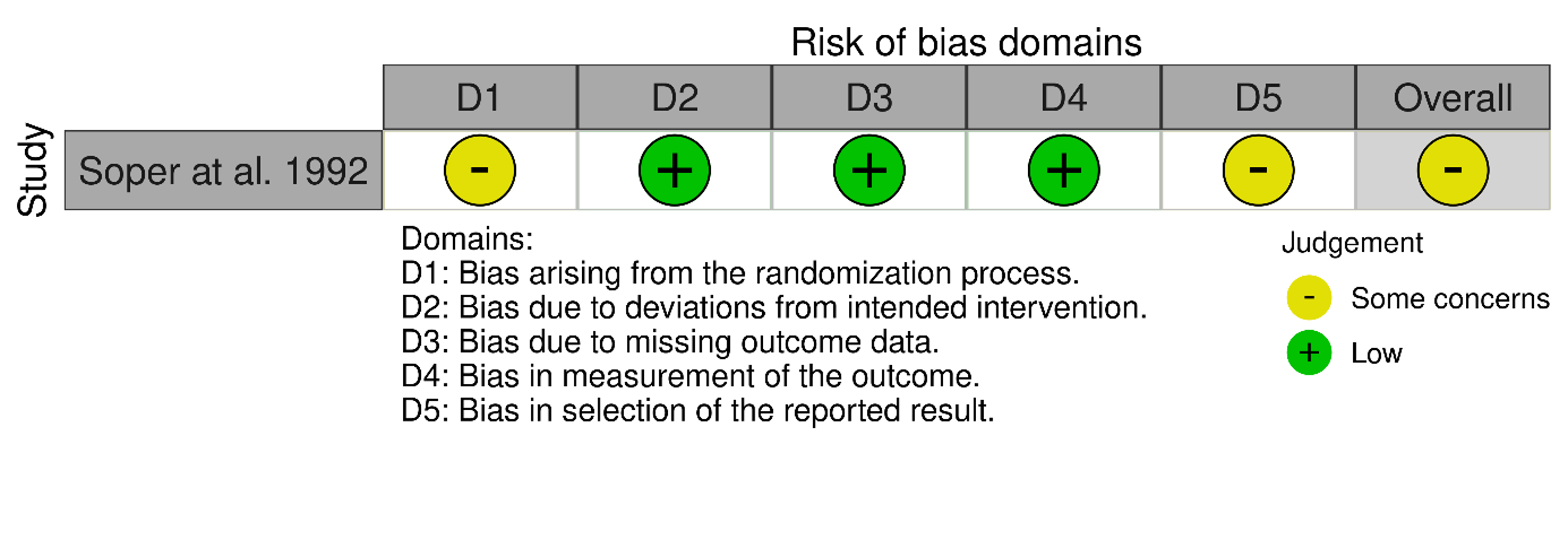

Supplement: Supplementary file 38 — Supplementary file38 (TIF 695 KB) [file 464_2022_9267_MOESM38_ESM.tif]

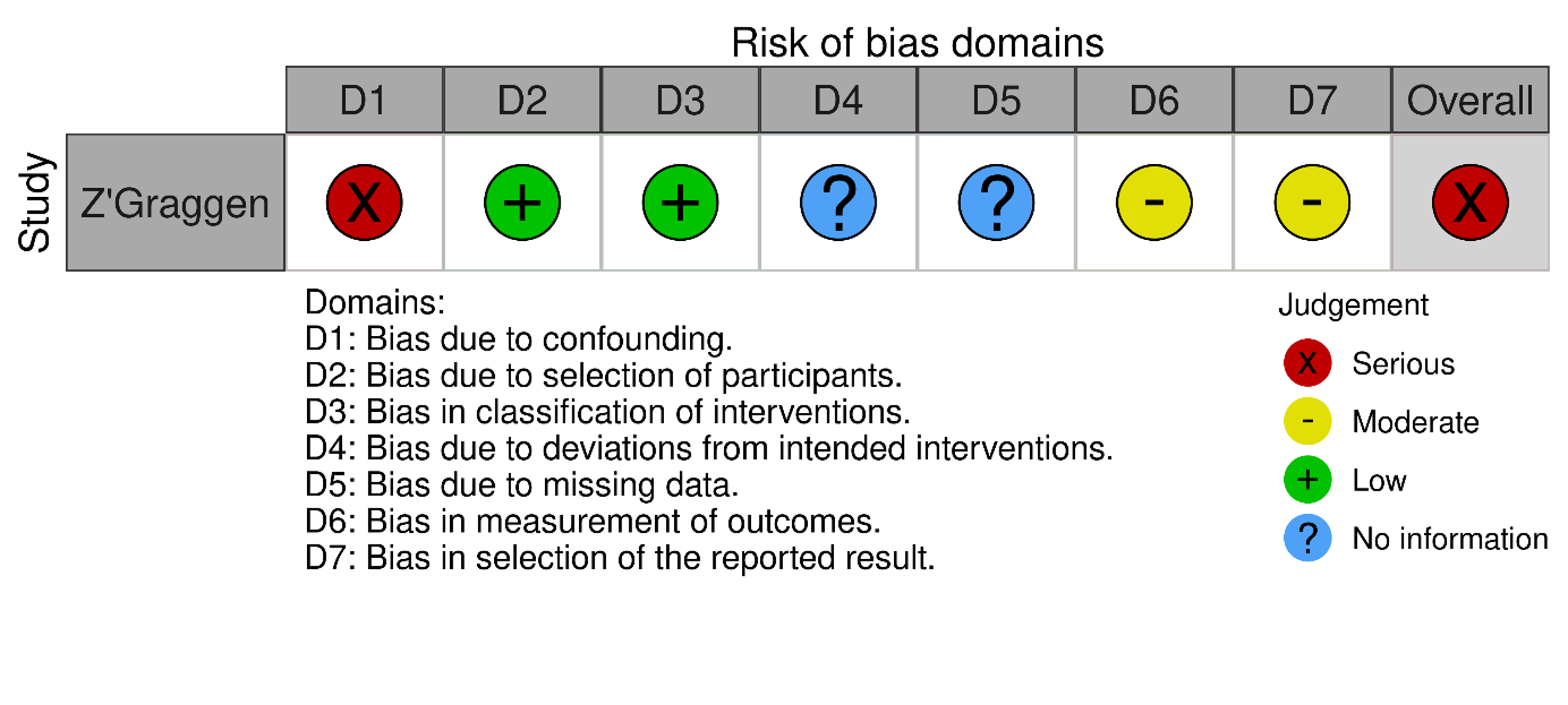

Supplement: Supplementary file 39 — Supplementary file39 (TIF 964 KB) [file 464_2022_9267_MOESM39_ESM.tif]

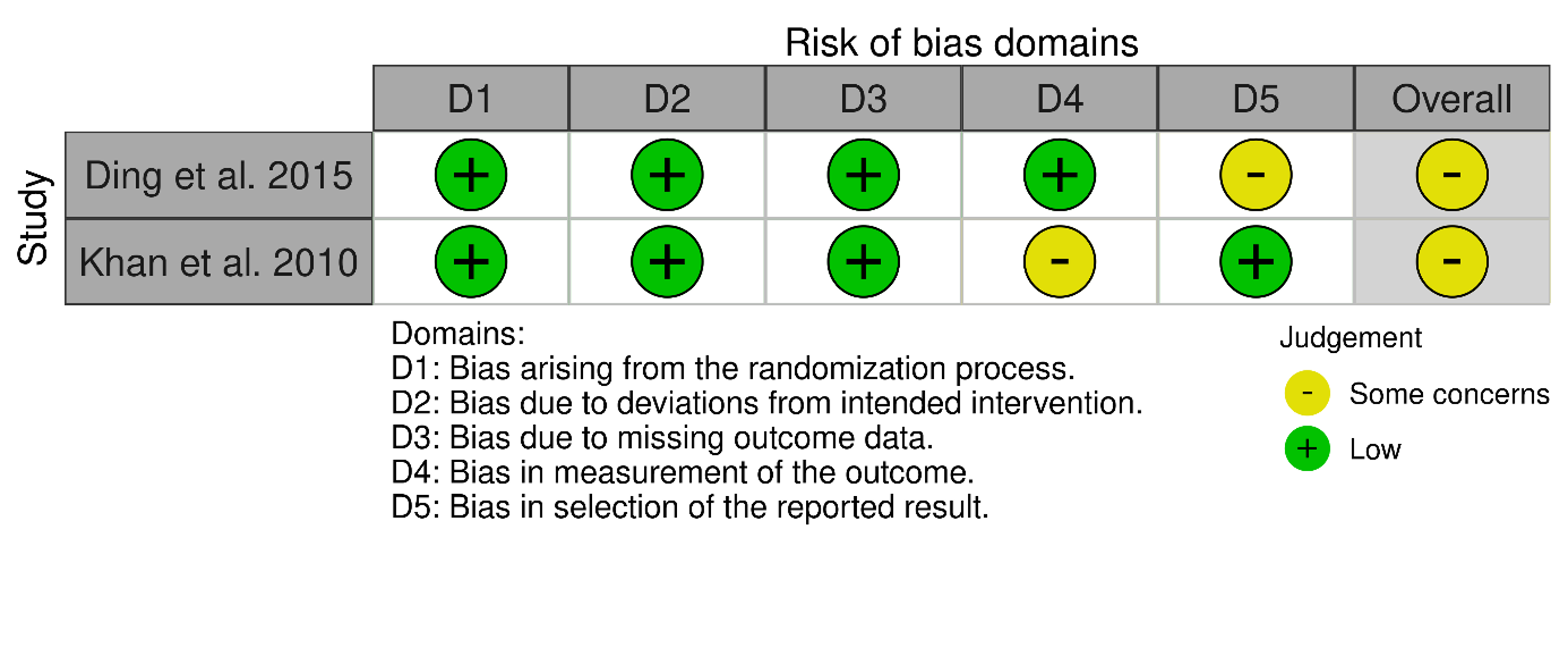

Supplement: Supplementary file 40 — Supplementary file40 (TIF 868 KB) [file 464_2022_9267_MOESM40_ESM.tif]

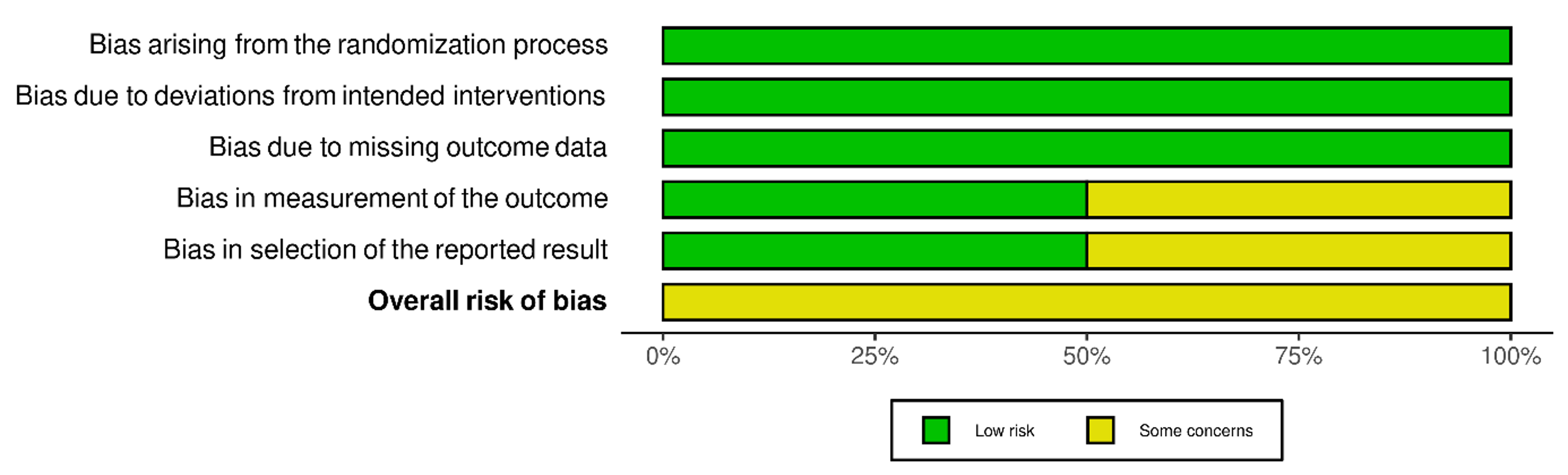

Supplement: Supplementary file 41 — Supplementary file41 (TIF 416 KB) [file 464_2022_9267_MOESM41_ESM.tif]

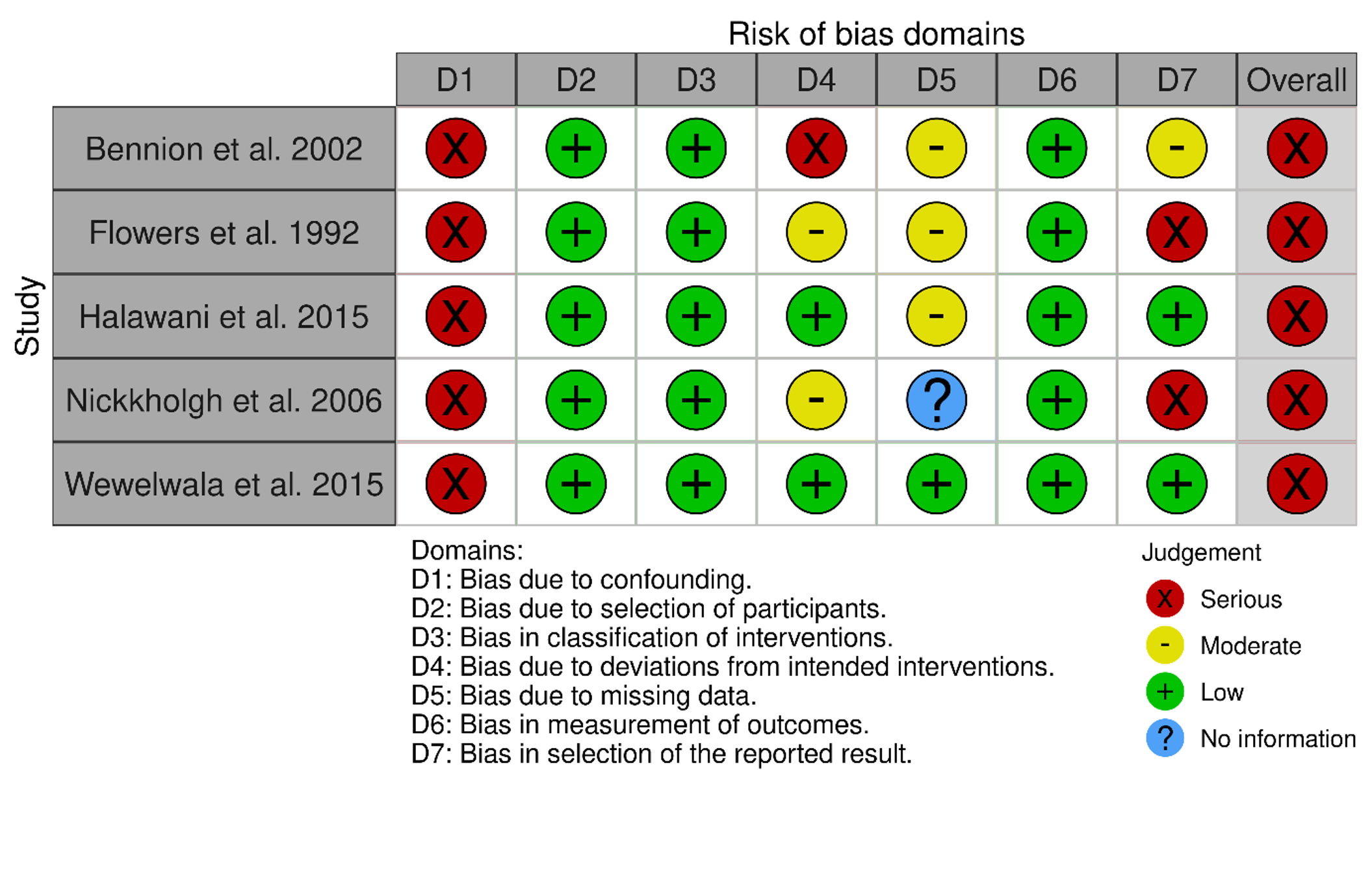

Supplement: Supplementary file 42 — Supplementary file42 (TIF 1138 KB) [file 464_2022_9267_MOESM42_ESM.tif]

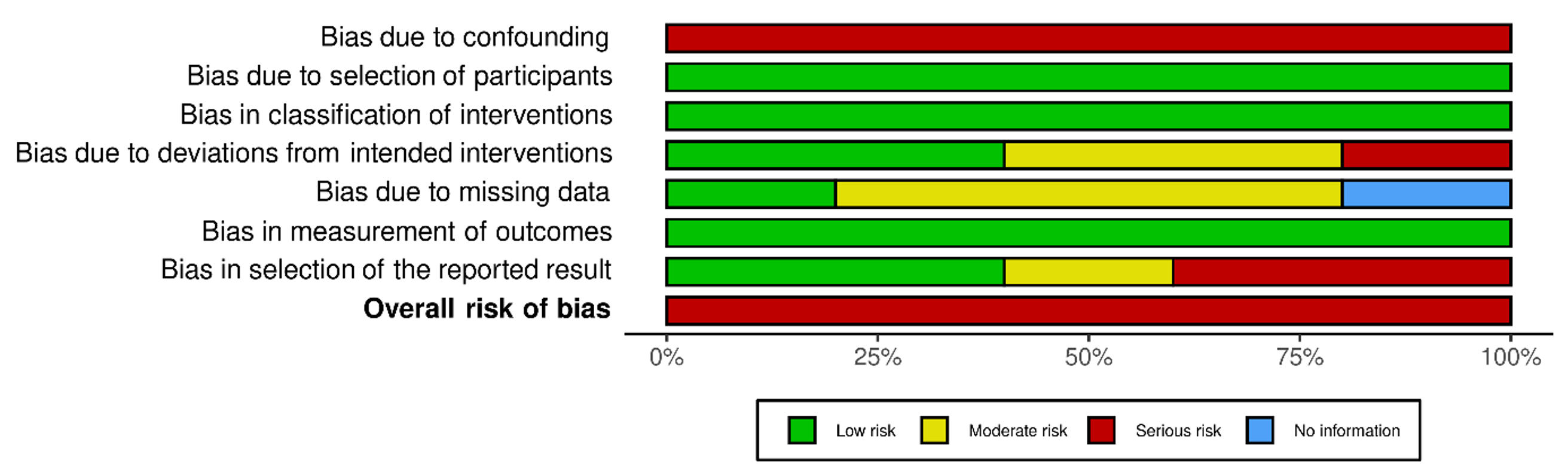

Supplement: Supplementary file 43 — Supplementary file43 (TIF 483 KB) [file 464_2022_9267_MOESM43_ESM.tif]

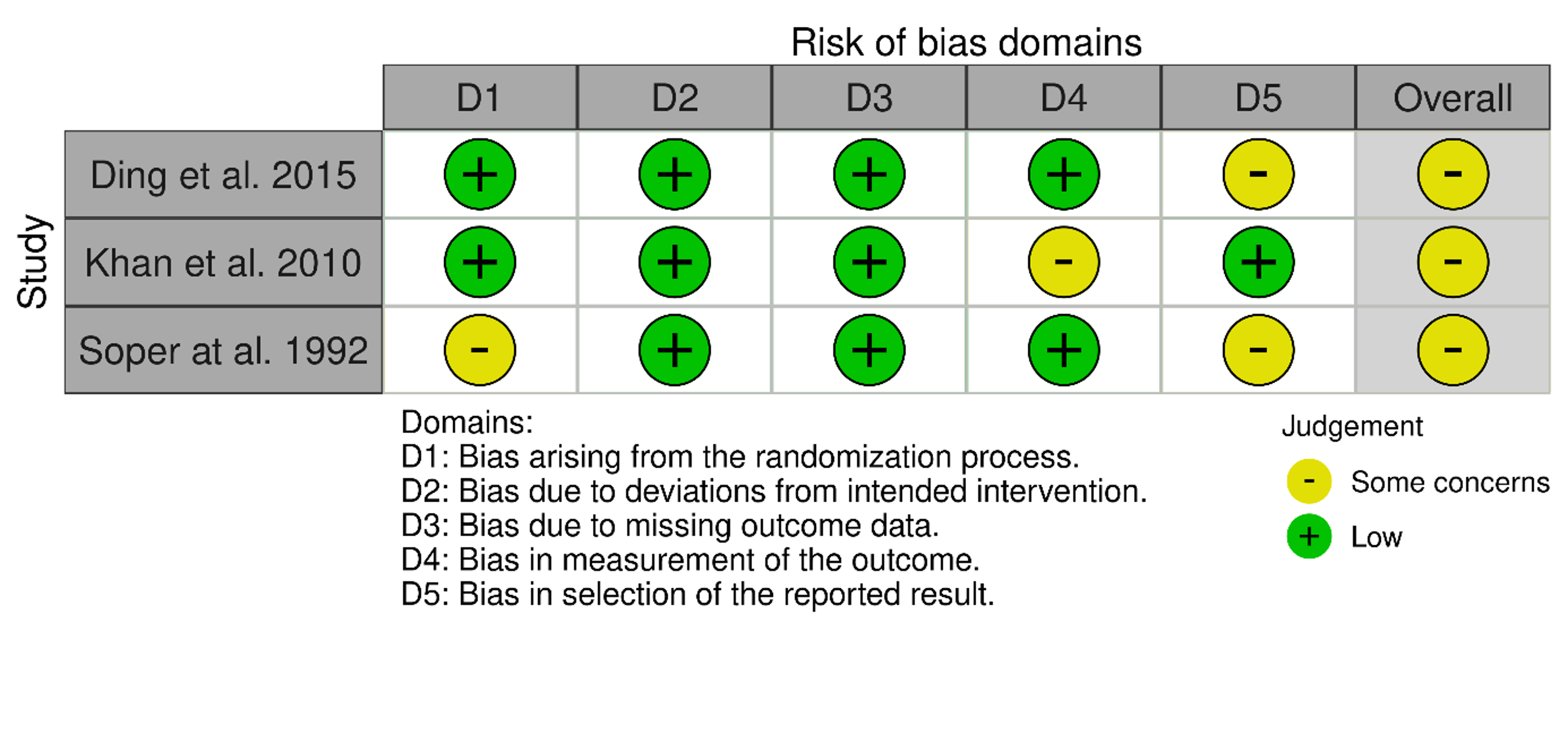

Supplement: Supplementary file 44 — Supplementary file44 (TIF 1016 KB) [file 464_2022_9267_MOESM44_ESM.tif]

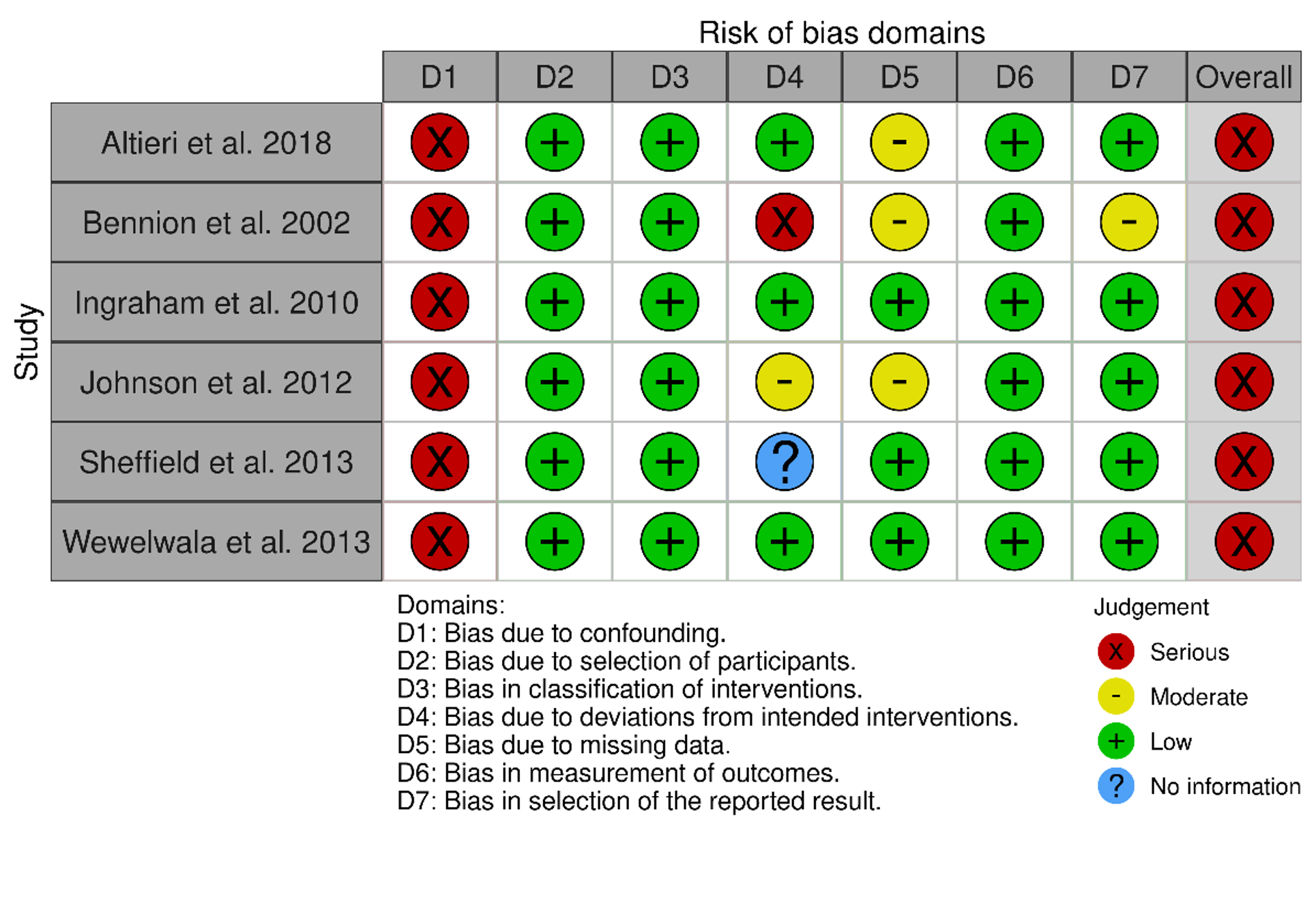

Supplement: Supplementary file 46 — Supplementary file46 (TIF 1162 KB) [file 464_2022_9267_MOESM46_ESM.tif]

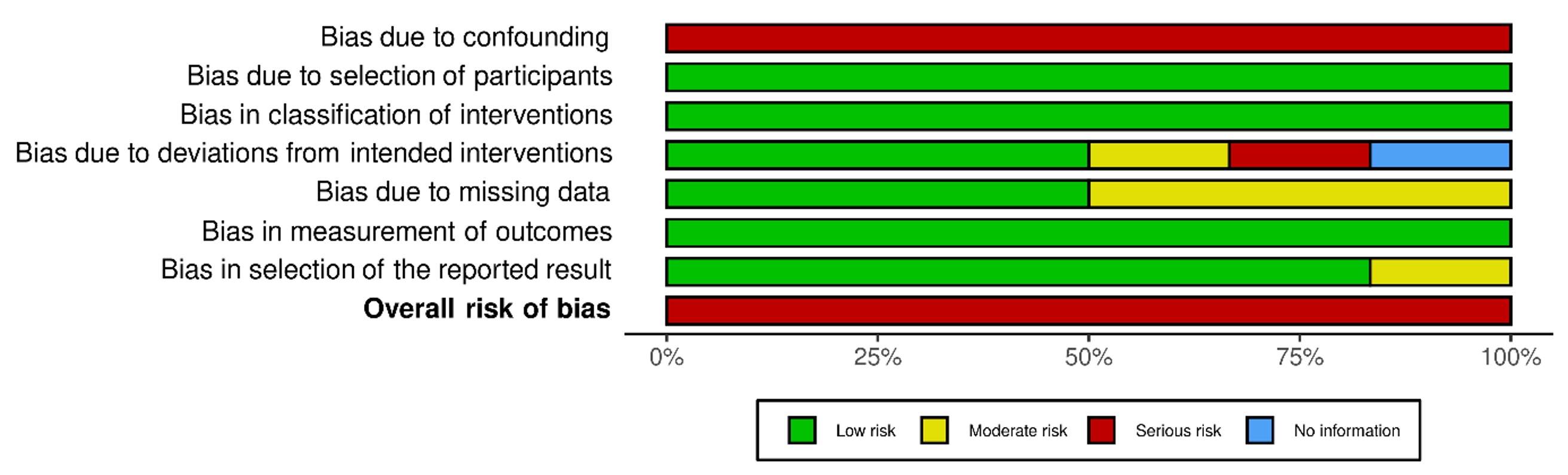

Supplement: Supplementary file 47 — Supplementary file47 (TIF 482 KB) [file 464_2022_9267_MOESM47_ESM.tif]
