# Supplementary material for: Selective intraoperative cholangiography should be considered over routine intraoperative cholangiography during cholecystectomy: a systematic review and meta-analysis
Source: Surg Endosc. 2022 Jul 7;36(10):7126–39. doi: 10.1007/s00464-022-09267-x (PMC9485186; doi:10.1007/s00464-022-09267-x)
Supplement: Supplementary file 50 — Supplementary file50 (DOCX 26 KB) [file 464_2022_9267_MOESM50_ESM.docx]

Supplementary Table 1: Characteristics of included studies (IOC vs no IOC)

| Study | Study design | Center(s) | Type of procedure | Comparison | Number of patients (female %, mean age±SD) | Outcomes | Follow up |
| --- | --- | --- | --- | --- | --- | --- | --- |
| Altieri et al. 2018 | Retrospective cohort | Multicentric (SPARCS) in USA | LC | IOC | 45873 | BDI, readmission rate, LOHS | 30-days |
|  |  |  |  | No IOC | 346612 |  |  |
| Bennion et al. 2002 | Retrospective cohort | Single center in USA | Cholecystectomy | IOC | 59 (83%, 38.2±16.3) | Retained stone rate, readmission rate, LOHS, operation time | Range (min:2 days, max: 10 months) |
|  |  |  |  | No IOC | 141 (86%, 36.3±12.6) |  |  |
| Borie et al. 2020 | Retrospective cohort | Multicentric (REX database) | Cholecystectomy | IOC | 623 (61%, 65±15) | BDI | N/A |
|  |  |  |  | No IOC | 538 (63%, 66±17) |  |  |
| Ding et al. 2015 | Randomized controlled trial | Single center in China | LC | IOC | 186 (54%, 57.43±7.15) | BDI, conversion rate to open surgery, success rate of IOC, operation time, LOHS | N/A |
|  |  |  |  | No IOC | 185 (52%, 58.22±8.41) |  |  |
| Fletcher et al. 1999 | Retrospective cohort | Multicentric (Health Department of Western Australia's Hospital Morbidity Data System) in Australia | Cholecystectomy | IOC | 11029 | BDI | N/A |
|  |  |  |  | No IOC | 8157 |  |  |
| Flowers et al. 1992 | Prospective cohort | Single center in USA | LC | IOC | 165 | Retained stone rate, operation time | max 15 months (ranged between 3-15 months) |
|  |  |  |  | No IOC | 199 |  |  |
| Flum et al. 2001 | Retrospective cohort | Multicenter (138 hospital, statewide, Washington State Comprehensive Hospital Abstract Reporting System database) | LC | IOC | 19514 | BDI | N/A |
|  |  |  |  | No IOC | 11116 |  |  |
| Flum et al. 2003 | Retrospective cohort | Multicenter (nationwide, Medicare) | Cholecystectomy | IOC | 613706 (62.6%, 71.7±10.3) | BDI | N/A |
|  |  |  |  | No IOC | 956655 (63.2%, 71.2±10.7) |  |  |
| Giger et al. 2010 | Prospective cohort | Multicenter (114) in Switzerland | LC | IOC | 11642 | BDI | N/A |
|  |  |  |  | No IOC | 20196 |  |  |
| Halawani et al. 2015 | Retrospective cohort | Multicenter (570 hospitals, ACS NSQIP) in USA and + 43 hospitals located elsewhere | LC | IOC | 11227 (71.91%, 50.18 ± 17.10) | Readmission rate, operation time | 30-day |
|  |  |  |  | No IOC | 41598 (73.75%, 48.63± 16.72) |  |  |
| Ingraham et al. 2010 | Retrospective cohort | Multicenter (221 hospitals, ACS NSQIP) in USA | LC | IOC | 15,480 | LOHS | N/A |
|  |  |  |  | No IOC | 42,777 |  |  |
| Johnson et al. 2012 | Retrospective cohort | Multicenter (12 hospitals) in Canada | Cholecystectomy | IOC | 119 (64.7%, 55, range: 19-94) | LOHS | N/A |
|  |  |  |  | No IOC | 213 (65.2%, 56, range: 18-91) |  |  |
| Khalili et al. 1997 | Retrospective cohort | Single center in USA | LC | IOC | 1207 (67%, 55, range: 11-99) | BDI, success rate of IOC | N/A |
|  |  |  |  | No IOC | 116 (74%, 51, range: 13-85) |  |  |
| Khan et al. 2010 | Randomized controlled trial | Single center in UK | LC | IOC | 91 (84%, 59, S.E.M: 2) | BDI, readmission rate, conversion rate to open surgery, | 12 months |
|  |  |  |  | No IOC | 99(76%, 53, S.E.M: 2) |  |  |
| Lilley et al. 2017 | Retrospective cohort | Multicentric (Medicare) in USA | Cholecystectomy | IOC | 165471 | BDI | N/A |
|  |  |  |  | No IOC | 306896 |  |  |
| Mangieri et al. 2019 | Retrospective cohort | Multicenter (700, ACS NSQIP) in USA and Canada | LC | IOC | 51673 | BDI | N/A |
|  |  |  |  | No IOC | 166102 |  |  |
| Rosero et al. 2017 | Retrospective cohort | Multicentric (890) in USA | LC | IOC | 48718 | Readmission rate | 30-day |
|  |  |  |  | No IOC | 181562 |  |  |
| Russel et al. 1996 | Retrospective cohort | Multicentric (30) in USA | LC | IOC | 3348 | BDI | N/A |
|  |  |  |  | No IOC | 11873 |  |  |
| Sheffield et al. 2013 | Retrospective cohort | Multi center (307 Texas hospitals, Medicare) | Cholecystectomy | IOC | 37533 (61.87%, 75.13± 6.61) | BDI, LOHS | N/A |
|  |  |  |  | No IOC | 55399 (61.61%, 75.17 ± 6.64) |  |  |
| Soper et al. 1992 | Randomized controlled trial | Single center in USA | LC | IOC | 56 (73%, 51± 2) | BDI, retained stone rate, LOHS, operation time | 2-12 month |
|  |  |  |  | No IOC | 59 (75%, 48± 2) |  |  |
| Tabone et al. 2011 | Retrospective cohort | Single center in USA | LC | IOC | 266 (66.9%, 49 ± 18.7) | Retained stone rate, readmission rate | mean: 22 days (range: 1-112) |
|  |  |  |  | No IOC | 1042 (78.6%, 46 ± 16.3) |  |  |
| Törnqvist et al. 2009 | Retrospective cohort | Multicentric (Swedish inpatient registry) in Sweden | Cholecystectomy | IOC | 256714 | BDI | N/A |
|  |  |  |  | No IOC | 117328 |  |  |
| Törnqvist et al. 2015 | Retrospective cohort | Multicentric (Gallriks) in Sweden | Cholecystectomy | IOC | 44401 | BDI | N/A |
|  |  |  |  | No IOC | 6253 |  |  |
| Verma et al. 2016 | Prospective cohort | Single center in Austria | LC | IOC | 38 | Retained stone rate, readmission rate, operation time | N/A |
|  |  |  |  | No IOC | 37 |  |  |
| Wewelwala et al. 2015 | Retrospective cohort | Multicentric (4) in Austria | LC | IOC | 147 (64%, 52 ± 18) | LOHS, operation time | N/A |
|  |  |  |  | No IOC | 30 (67%, 52 ± 18) |  |  |
| Z'graggen et al. 1998 | Prospective cohort | Multicentric (82) in Switzerland | LC | IOC | 2585 | BDI, conversion rate to open surgery, operation time | N/A |
|  |  |  |  | No IOC | 7589 |  |  |

^IOC= intraoperative cholangiography^

^LC= laparoscopic cholecystectomy^

^BDI= bile duct injury^

^LOHS= length of hospital stay^
