# Supplementary material for: Selective intraoperative cholangiography should be considered over routine intraoperative cholangiography during cholecystectomy: a systematic review and meta-analysis
Source: Surg Endosc. 2022 Jul 7;36(10):7126–39. doi: 10.1007/s00464-022-09267-x (PMC9485186; doi:10.1007/s00464-022-09267-x)
Supplement: Supplementary file 51 — Supplementary file51 (DOCX 17 KB) [file 464_2022_9267_MOESM51_ESM.docx]

Supplementary Table 2: Characteristics of included studies (selective IOC vs no IOC)

| Study | Study design | Center(s) | Type of procedure | Comparison | Number of patients (female %, mean age±SD) | Outcomes | Follow up |
| --- | --- | --- | --- | --- | --- | --- | --- |
| Misra et al. 2005 | Retrospective cohort | Single center in USA | LC | Selective IOC | 193 | BDI, retained stone rate | 1 month to 7 years |
|  |  |  |  | No IOC | 761 |  |  |
| Robinson et al. 1995 | Retrospective cohort | Single center in USA | LC | Selective IOC | 161 | Retained stone rate | N/A |
|  |  |  |  | No IOC | 334 |  |  |
| Zang et al. 2016 | Retrospective cohort | Single center in China | LC | Selective IOC | 213 (52%, 54.3 ± 9.1) | Retained stone rate | 1 year |
|  |  |  |  | No IOC | 1759 |  |  |

^IOC= intraoperative cholangiography^

^LC= laparoscopic cholecystectomy^
