# Supplementary material for: Selective intraoperative cholangiography should be considered over routine intraoperative cholangiography during cholecystectomy: a systematic review and meta-analysis
Source: Surg Endosc. 2022 Jul 7;36(10):7126–39. doi: 10.1007/s00464-022-09267-x (PMC9485186; doi:10.1007/s00464-022-09267-x)
Supplement: Supplementary file 52 — Supplementary file52 (DOCX 18 KB) [file 464_2022_9267_MOESM52_ESM.docx]

Supplementary Table 3: Indications for selective IOC

| Study | ROUTINE IOC vs SELECTIVE IOC | | | | |
| --- | --- | --- | --- | --- | --- |
|  | **Jaundice** | **Dilated CBD** | **Stone on imaging** | **Pancreatitis** | **Abnormal liver function** |
| Alkhaffaf et al. 2011 | history | UNS | MR | history of ABP | ALP >150 U/L and ALT > 35 U/L |
| Amott et al. 2005 | N/A | on US | N/A | N/A | d/iBi, AST, ALP |
| Buddingh et al. 2011 | N/A | on US | N/A | N/A | UNS |
| Carlson et al. 1993 | history | on US | US | N/A | Bi or ALP |
| Guerra-Filho et al. 2007 | history or presence | UNS | UNS | history | Bi or Amylase or ALP |
| Nickkholgh et al. 2006 | history | on US | US | history | LFT |
| Pham et al. 2016 | N/A | UNS | N/A | N/A | Bi |
| Ragulin-Coyne et al. 2013 | N/A | N/A | N/A | N/A | N/A |
| Snow et al. 2001 | history or presence | UNS | US | UNS | Bi or ALT or AST or LDH or ALP or Amylase |
|  | **SELECTIVE IOC vs NO IOC** | | | | |
| Misra et al. 2005 | N/A | N/A | N/A | presence of ABP | LFT |
| Robinson et al. 1995 | history | UNS | N/A | history | Bi or AST or ALP increased |
| Zang et al. 2016 | history | US | US | UNS | LFT |

^UNS= unspecified^

^N/A= not available^

^CBD= common bile duct^

^US= ultrasound^

^ALP= alkaline phosphatase^

^AST= aspartate aminotransferase^

^ALT= alanine aminotransferase^

^d/i Bi= direct/ indirect bilirubin^

^LFT= liver function tests^

^ABP= acute biliary pancreatitis^
