# Supplementary material for: Selective intraoperative cholangiography should be considered over routine intraoperative cholangiography during cholecystectomy: a systematic review and meta-analysis
Source: Surg Endosc. 2022 Jul 7;36(10):7126–39. doi: 10.1007/s00464-022-09267-x (PMC9485186; doi:10.1007/s00464-022-09267-x)
Supplement: Supplementary file 53 — Supplementary file53 (DOCX 21 KB) [file 464_2022_9267_MOESM53_ESM.docx]

Supplementary Table 4: Qualitative synthesis of the included publications (dichotomous variables)

| **Author** | **Number of patients** | **Event number (group 1)** | **Event number (group 2)** | **Follow-up** | **P-value** |
| --- | --- | --- | --- | --- | --- |
| **BDI, MBDI** | | | | | |
| *routine IOC vs selective IOC* | | | | | |
| Carlson et al. 1993 | **319** | **1** | **0** | **-** | **n/A** |
| *IOC vs no IOC* | | | | | |
| Khalili et al. 1997 | 1323 | 5 | 0 | - | 0.48 |
|  | | | | | |
| **RETAINED STONES RATE** | | | | | |
| *routine IOC vs selective IOC* | | | | | |
| Amott et al. 2005 | 315 | 3 | 5 | 12 months | 0,19 |
| Carlson et al. 1993 | 319 | 0 | 1 | A inst.: 9-28 months B inst.: 16-31 months | N/A |
| Nickkholgh et al. 2006 | 2130 | 2 | 9 | N/A | <0,01***** |
| Pham et al. 2016 | 520 | 0 | 2 | 30 days | 1 |
| Snow et al. 2001 | 2043 | 0 | 0 | <11 year | N/A |
| *IOC vs no IOC* | | | | | |
| Flowers et al. 1992 | 364 | 1 | 2 | Range: 3-15 months | N/A |
| *selective IOC vs no IOC* | | | | | |
| Misra et al. 2005 | 954 | 0 | 7 | >7 years | N/A |
| Robinson et al. 1995 | 495 | 3 | 4 | mean: 25 months | N/A |
| Zang et al. 2016 | 1972 | 3 | 8 | 12 months | N/A |
|  | | | | | |
| **READMISSION RATE** | | | | | |
| *IOC vs no IOC* | | | | | |
| Bennion et al. 2002 | 200 | 3 | 7 | 2 days- 10 months | N/A |
| Khan et al. 2010 | 190 | 0 | 4 | 12 months | 0,122 |
| Tabone et al. 2011 | 1308 | 0 | 31 | mean: 22 days (range: 1-112) | N/A |
| Verma et al. 2016 | 75 | 0 | 0 | median: 15 months | N/A |
|  | | | | | |
| **CONVERSION RATE TO OPERN SURGERY** | | | | | |
| *routine IOC vs selective IOC* | | | | | |
| Alkhaffaf et al. 2011 | 1630 | 10 | 83 | N/A | 0,001***** |
| Buddingh et al. 2011 | 835 | 65 | 57 | N/A | 0,527 |
|  | | | | | |
| **SUCCESS RATE OF IOC** | | | | | |
| *routine IOC vs selective IOC* | | | | | |
| Buddingh et al. 2011 | **835** | **225** | **23** | **N/A** | **N/A** |

^Inst= institution^

^*statistically significant result (p^ ^< 0.05)^
