# Supplementary material for: Selective intraoperative cholangiography should be considered over routine intraoperative cholangiography during cholecystectomy: a systematic review and meta-analysis
Source: Surg Endosc. 2022 Jul 7;36(10):7126–39. doi: 10.1007/s00464-022-09267-x (PMC9485186; doi:10.1007/s00464-022-09267-x)
Supplement: Supplementary file 54 — Supplementary file54 (DOCX 22 KB) [file 464_2022_9267_MOESM54_ESM.docx]

Supplementary Table 5: Qualitative synthesis of the included publications (continuous variables)

| **Author** | **Total number of patients** | **Group 1** | | | | | | | | **Group 2** | | | | | | | | **P-value** |
| --- | --- | --- | --- | --- | --- | --- | --- | --- | --- | --- | --- | --- | --- | --- | --- | --- | --- | --- |
|  |  | **Number of patients** | **Mean** | **Median** | **Standard deviation** | **Interquartile range min** | **Interquartile range max** | **Range min** | **Range max** | **Number of patients** | **Mean** | **Median** | **Standard deviation** | **Interquartile range min** | **Interquartile range max** | **Range min** | **Range max** |  |
| **OPERATION TIME** | | | | | | | | | | | | | | | | | | |
| *routine IOC vs selective IOC* | | | | | | | | | | | | | | | | | | |
| Buddingh et al. 2011 | 835 | 435 | 110 |  | 44 |  |  |  |  | 421 | 100 |  | 47 |  |  |  |  | 0,001* |
| *IOC vs no IOC* | | | | | | | | | | | | | | | | | | |
| Flowers et al. 1992 | 364 | 165 | 116 |  | 15 |  |  |  |  | 199 | 68.42 | 52 |  |  |  |  |  | N/A |
|  | | | | | | | | | | | | | | | | | | |
| **LENGTH OF HOSPITAL STAY** | | | | | | | | | | | | | | | | | | |
| *IOC vs no IOC* | | | | | | | | | | | | | | | | | | |
| Tabone et al. 2011 | 1042 |  | 3,4 |  |  |  |  |  |  |  | 2,6 |  |  |  |  |  |  | 0,01***** |
| *routine IOC vs selective IOC* | | | | | | | | | | | | | | | | | | |
| Alkhaffaf et al. 2011 | 1630 | 463 |  | 2 |  | 1 | 2 |  |  | 1159 |  | 2 |  | 1 | 3 |  |  | 0,54 |
| Pham et al. 2016 | 520 | 246 |  | 2 |  | 1 | 3 |  |  | 274 |  | 1 |  | 1 | 1 |  |  | 0,001***** |
| Ragulin-Coyne et al. 2013 | 111815 | 13025 |  | 2 |  |  |  |  |  | 98790 |  | 2 |  |  |  |  |  | 0,0008***** |

^*statistically significant result (p^ ^< 0.05)^
