# Supplementary material for: Selective intraoperative cholangiography should be considered over routine intraoperative cholangiography during cholecystectomy: a systematic review and meta-analysis
Source: Surg Endosc. 2022 Jul 7;36(10):7126–39. doi: 10.1007/s00464-022-09267-x (PMC9485186; doi:10.1007/s00464-022-09267-x)
Supplement: Supplementary file 55 — Supplementary file55 (DOCX 16 KB) [file 464_2022_9267_MOESM55_ESM.docx]

Supplementary Table 6: GRADE evidence profile – Comparison: IOC vs no IOC. Population: both type of cholecystectomy

| **Certainty assessment** | | | | | | | **№ of patients** | | **Effect** | | **Certainty** | **Importance** |
| --- | --- | --- | --- | --- | --- | --- | --- | --- | --- | --- | --- | --- |
| **№ of studies** | **Study design** | **Risk of bias** | **Inconsistency** | **Indirectness** | **Imprecision** | **Other considerations** | **IOC** | **no IOC** | **Relative (95% CI)** | **Absolute (95% CI)** |  |  |
| **BDI (both type of cholecystectomy) (assessed with: RR)** | | | | | | | | | | | | |
| 14 | observational studies | very serious^a^ | very serious^b^ | very serious^c^ | not serious | none | 4919/1207398 (0.4%) | 8000/1948542 (0.4%) | **RR 1.03** (0.77 to 1.37) | **0 fewer per 1 000** (from 1 fewer to 2 more) | ⨁◯◯◯ Very low | CRITICAL |
| **Major bile duct injury (both type of cholecystectomy) (assessed with: RR)** | | | | | | | | | | | | |
| 9 | observational studies | very serious^a^ | very serious^b^ | very serious^c^ | not serious | publication bias strongly suspected^d^ | 4013/1042780 (0.4%) | 7134/1401338 (0.5%) | **RR 1.01** (0.70 to 1.45) | **0 fewer per 1 000** (from 2 fewer to 2 more) | ⨁◯◯◯ Very low | CRITICAL |
| **Length of hospital stay (both type of cholecystectomy) (assessed with: WMD)** | | | | | | | | | | | | |
| 8 | observational studies | very serious^a^ | very serious^b^ | very serious^c^ | not serious | publication bias strongly suspected^d^ | 99719 | 446458 | - | WMD **0.03 day fewer** (0.26 fewer to 0.2 more) | ⨁◯◯◯ Very low | IMPORTANT |

**^CI:^** ^confidence interval;^ **^RR:^** ^risk ratio^

#### ^Explanations^

^a. Bias is likely due to the presence of confounding factors.^

^b. Inconsistency is likely due to the presence of statistically significant heterogeneity.^

^c. Indirect population is likely due to the variable inclusion and exclusion criteria.^

^d. Publication bias is likely due to funnel plot asymmetry.^
