# Supplementary material for: Selective intraoperative cholangiography should be considered over routine intraoperative cholangiography during cholecystectomy: a systematic review and meta-analysis
Source: Surg Endosc. 2022 Jul 7;36(10):7126–39. doi: 10.1007/s00464-022-09267-x (PMC9485186; doi:10.1007/s00464-022-09267-x)
Supplement: Supplementary file 56 — Supplementary file56 (DOCX 20 KB) [file 464_2022_9267_MOESM56_ESM.docx]

Supplementary Table 7: GRADE evidence profile – Comparison: IOC vs no IOC. Population: laparoscopic cholecystectomy

| **Certainty assessment** | | | | | | | **№ of patients** | | **Effect** | | **Certainty** | **Importance** |
| --- | --- | --- | --- | --- | --- | --- | --- | --- | --- | --- | --- | --- |
| **№ of studies** | **Study design** | **Risk of bias** | **Inconsistency** | **Indirectness** | **Imprecision** | **Other considerations** | **IOC** | **no IOC** | **Relative (95% CI)** | **Absolute (95% CI)** |  |  |
| **Bile duct injury (laparoscopic cholecystectomy) (assessed with: RR)** | | | | | | | | | | | | |
| 10 | observational studies | very serious^a^ | very serious^b^ | very serious^c^ | not serious | none | 340/138365 (0.2%) | 818/567971 (0.1%) | **RR 1.19** (0.79 to 1.79) | **0 fewer per 1 000** (from 0 fewer to 1 more) | ⨁◯◯◯ Very low | CRITICAL |
| **Bile duct injury (laparoscopic cholecystectom- only prospective studies pooled) (assessed with: RR)** | | | | | | | | | | | | |
| 5 | observational studies | very serious^a^ | not serious | serious^e^ | not serious | none | 49/14560 (0.3%) | 87/28128 (0.3%) | **RR 1.09** (0.77 to 1.54) | **0 fewer per 1 000** (from 1 fewer to 2 more) | ⨁◯◯◯ Very low | CRITICAL |
| **Major bile duct injury (laparoscopic cholecystectomy) (assessed with: RR)** | | | | | | | | | | | | |
| 5 | observational studies | very serious^a^ | serious^b^ | not serious | not serious | publication bias strongly suspected^d^ | 57/23195 (0.2%) | 60/23332 (0.3%) | **RR 1.09** (0.35 to 3.34) | **0 fewer per 1 000** (from 2 fewer to 6 more) | ⨁◯◯◯ Very low | CRITICAL |
| **Retained stone rate (laparoscopic cholecystectomy) (follow-up: range 1 days to 15 months; assessed with: RR)** | | | | | | | | | | | | |
| 5 | observational studies | very serious^a^ | not serious | serious^c^ | serious^f^ | publication bias strongly suspected^d^ | 2/605 (0.3%) | 35/1464 (2.4%) | **RR 0.51** (0.12 to 2.11) | **12 fewer per 1 000** (from 21 fewer to 27 more) | ⨁◯◯◯ Very low | CRITICAL |
| **Readmission rate (laparoscopic cholecystectomy (follow-up: range 1 days to 30 days; assessed with: RR)** | | | | | | | | | | | | |
| 4 | observational studies | very serious^a^ | very serious^b^ | not serious | serious^g^ | publication bias strongly suspected^d^ | 3177/105874 (3.0%) | 19736/569831 (3.5%) | **RR 0.92** (0.79 to 1.06) | **3 fewer per 1 000** (from 7 fewer to 2 more) | ⨁◯◯◯ Very low | IMPORTANT |
| **Conversion rate (laparoscopic cholecystectomy) (assessed with: RR)** | | | | | | | | | | | | |
| 3 | observational studies | very serious^a^ | not serious | not serious | not serious | publication bias strongly suspected^h^ | 119/2862 (4.2%) | 542/7873 (6.9%) | **RR 0.64** (0.51 to 0.78) | **25 fewer per 1 000** (from 34 fewer to 15 fewer) | ⨁◯◯◯ Very low | IMPORTANT |
| **Operation time (laparoscopic cholecystectomy) (assessed with: WMD)** | | | | | | | | | | | | |
| 7 | observational studies | very serious^a^ | very serious^b^ | very serious^c^ | not serious | publication bias strongly suspected^d^ | 13096 | 42912 | - | WMD **11.25 min more** (6.57 more to 15.93 more) | ⨁◯◯◯ Very low | IMPORTANT |
| **Lenght of hospital stay (laparoscopic cholecystectomy) (assessed with: WMD)** | | | | | | | | | | | | |
| 4 | observational studies | very serious^a^ | very serious^b^ | very serious^c^ | not serious | publication bias strongly suspected^h^ | 15869 | 43051 | - | WMD **0.04 day more** (0.12 fewer to 0.19 more) | ⨁◯◯◯ Very low | IMPORTANT |

**^CI:^** ^confidence interval;^ **^RR:^** ^risk ratio^

#### ^Explanations^

^a. Bias is likely due to the presence of confounding factors.^

^b. Inconsistency is likely due to the presence of statistically significant heterogeneity.^

^c. Indirect population is likely due to the variable inclusion and exclusion criteria.^

^d. Publication bias is likely due to funnel plot asymmetry.^

^e. The majority of the patients had acute or chronic cholecystitis as an indication for laparoscopic cholecystectomy.^

^f. Imprecision is likely because confidence intervals cross the benefit/harm line and 0-effect line.^

^g. Imprecision is likely due to the control event rate is less than 40%.^

^h. Publication bias was not assessed due to the low number of available publications.^
