# Supplementary material for: Selective intraoperative cholangiography should be considered over routine intraoperative cholangiography during cholecystectomy: a systematic review and meta-analysis
Source: Surg Endosc. 2022 Jul 7;36(10):7126–39. doi: 10.1007/s00464-022-09267-x (PMC9485186; doi:10.1007/s00464-022-09267-x)
Supplement: Supplementary file 57 — Supplementary file57 (PDF 89 KB) [file 464_2022_9267_MOESM57_ESM.pdf]

Selective intraoperative cholangiography should be considered over routine intraoperative cholangiography during cholecystectomy: A systematic review and meta-analysis

Aim: to review and analyse the available literature on the benefits of IOC during cholecystectomy

38 eligible articles

3,747,695 patients

Population:

Patients after cholecystectomy

- *Open*

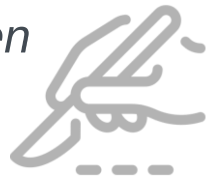

- *Laparoscopic*

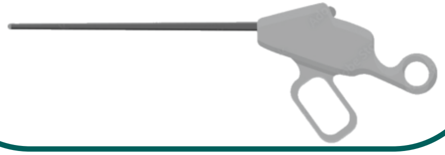

Main results

Bile duct injury

Routine IOC vs Selective IOC

RR= 0.91, 95% CI: 0.66; 1.24

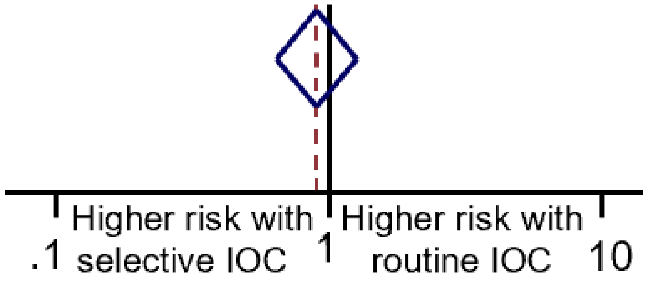

IOC vs non-IOC

RR= 1.03, 95% CI: 0.77; 1.37

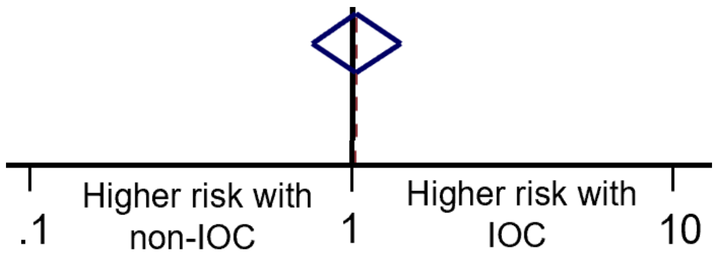

Conclusion: IOC might not be indicated in every case, and selective use may stand as an alternative to routine policy
